# Enzymatic Synthesis of 2-Chloropurine Arabinonucleosides with Chiral Amino Acid Amides at the C6 Position and an Evaluation of Antiproliferative Activity In Vitro

**DOI:** 10.3390/ijms24076223

**Published:** 2023-03-25

**Authors:** Barbara Z. Eletskaya, Maria Ya. Berzina, Ilya V. Fateev, Alexei L. Kayushin, Elena V. Dorofeeva, Olga I. Lutonina, Ekaterina A. Zorina, Konstantin V. Antonov, Alexander S. Paramonov, Inessa S. Muzyka, Olga S. Zhukova, Mikhail V. Kiselevskiy, Anatoly I. Miroshnikov, Roman S. Esipov, Irina D. Konstantinova

**Affiliations:** 1 Shemyakin and Ovchinnikov Institute of Bioorganic Chemistry, Russian Academy of Sciences, Miklukho-Maklaya St. 16/10, 117997 Moscow, Russia; 2 State N.N. Blokhin Russian Cancer Research Center, Kashirsky Highway, 24, 115478 Moscow, Russia

**Keywords:** arabinonucleosides, nucleoside phosphorylases, arsenolysis, adenosine deaminase, antiproliferative activity

## Abstract

A number of purine arabinosides containing chiral amino acid amides at the C6 position of the purine were synthesized using a transglycosylation reaction with recombinant *E. coli* nucleoside phosphorylases. Arsenolysis of 2-chloropurine ribosides with chiral amino acid amides at C6 was used for the enzymatic synthesis, and the reaction equilibrium shifted towards the synthesis of arabinonucleosides. The synthesized nucleosides were shown to be resistant to the action of *E. coli* adenosine deaminase. The antiproliferative activity of the synthesized nucleosides was studied on human acute myeloid leukemia cell line U937. Among all the compounds, the serine derivative exhibited an activity level (IC_50_ = 16 μM) close to that of Nelarabine (IC_50_ = 3 μM) and was evaluated as active.

## 1. Introduction

The involvement of adenosine and its nucleotides into numerous biological processes has led to many works on the synthesis of structural analogues of adenosine. Agonists and antagonists of adenosine receptors [1,2] represent the broadest group of adenosine analogs obtained. Compounds with antitumor activity have been found among A_3_-type adenosine receptor agonists [3,4]. N^6^-Allyl, N^6^-isopropyl, and N^6^-propargyl analogs have shown to significantly increase the lifespans of mice experiencing mammary carcinoma. The short-chain adenosine analogues are more active in the treatment of animal carcinomas than in leukemia or sarcoma tumor cell systems [5].

**Figure 1:**
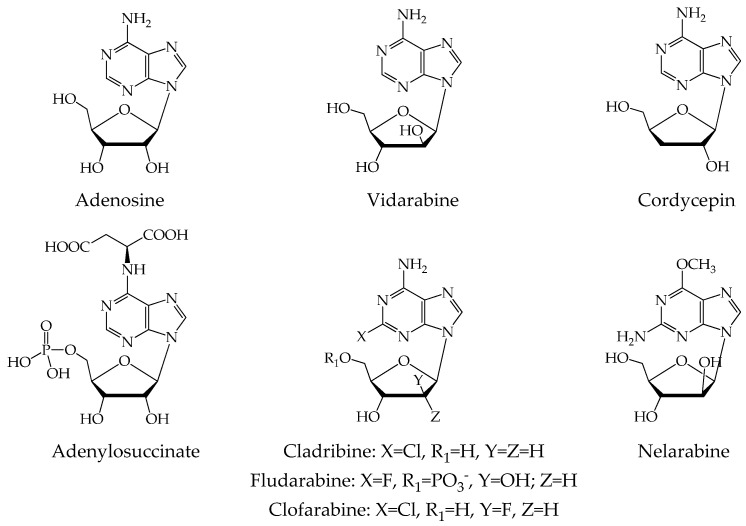
Biologically active purine nucleosides.

Establishment of the structure of the adenylosuccinate (Figure 1) involved in the purine nucleotide cycle prompted researchers to synthesize a series of purine bases substituted at C6 by chiral amino acid residues [6,7,8,9]. Conjugates were synthesized both with amino acids [10,11] and with corresponding methyl esters [10,12]. Conjugates with amino acids have antimycobacterial activity, but no antitumor activity [6,7]. Interestingly, the arabinoside analogue of natural N^6^-isopentenyladenosine did not show anti-proliferative capacity on T24 human bladder carcinoma cells [13], while the N^6^-isopentenyladenosine riboside did.

Adenosine analogs lose their biological activity due to rapid degradation by intracellular adenosine deaminase (ADA). For example, the antiviral D-arabinofuranosyladenine (vidarabine or Ara-A) is rapidly deaminated in vivo to the less active D-arabinofuranosylhypoxantine (Ara-H) [14]. Cordycepin (3′-deoxyadenosine) was found to be ineffective as an antibacterial agent due to metabolic degradation by ADA [15].

To enhance the biological activity of the compounds, it was necessary to increase the lifetime of the compound in the cell. Attempts have been made to synthesize ADA-resistant purine nucleosides. The introduction of a halogen atom in the C2 position of purine inhibits the action of intracellular adenosine deaminase or makes the nucleoside completely resistant to deamination [16]. The antitumor drugs Cladribine, Fludarabine, and Clofarabine (Figure 1) are examples of such compounds. They are used in the treatment of oncohematological diseases [17,18,19].

**Figure 2:**
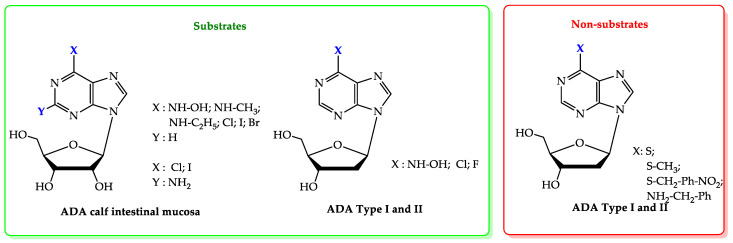
ADA substrates (green frame) and non-substrates (red frame). Data from ref. [20,21].

Another way to make nucleosides resistant to the catabolic action of ADA is introducing various substituents at the C6 position of adenosine. However, no unambiguous relationship between the substituent structure and ADA resistance was found. The rate of hydrolysis of adenosines decreased in a series of C6 substituents: hydroxylamino, chlorine, bromine, iodine, methylamino, and ethylamino (Figure 2). Deoxyadenosines with mercapto-, benzylamino-, and p-nitrobenzyl substituents at C6 were resistant to ADA [20,21,22].

On the other hand, ADA is a key intracellular enzyme that modifies the anticancer drug Nelarabine (9-β-D-arabinofuranosyl-6-O-methylguanine, Figure 1). A necessary step in the metabolism of Nelarabine is the demethoxylation of the purine base by ADA with the formation of 9-β-D-arabinofuranosyl guanine (Ara-G), which suppresses cell proliferation [23].

It is difficult to find a clear correlation between the antimetabolic activity of nucleosides and the modification of the C2 and C6 purine positions or the carbohydrate residue. We decided to synthesize a number of 2-chloro-6-substituted arabinonucleosides using enzymatic synthesis. The next step was to study both their resistance to ADA and their antiproliferative activity, in order to draw conclusions about the relationship between the structure of the nucleoside, its activity against ADA, and its antiproliferative activity.

## 2. Results and Discussion

### 2.1. Synthesis of Arabinonucleosides

Chemical modifications of purine are known to be more convenient to carry out in a nucleoside (where the N9 position of purine is protected by a carbohydrate residue) instead of a heterocyclic base [24,25]. The classical method for obtaining arabinonucleosides is the glycosylation of a modified purine base with a protected carbohydrate derivative [26,27,28,29] or complex ribose modifications [30,31]. Enzymatic methods for the synthesis of arabinosides [32,33,34] are very convenient due to the high selectivity and stereospecificity of enzymatic reactions.

**Scheme 1:**
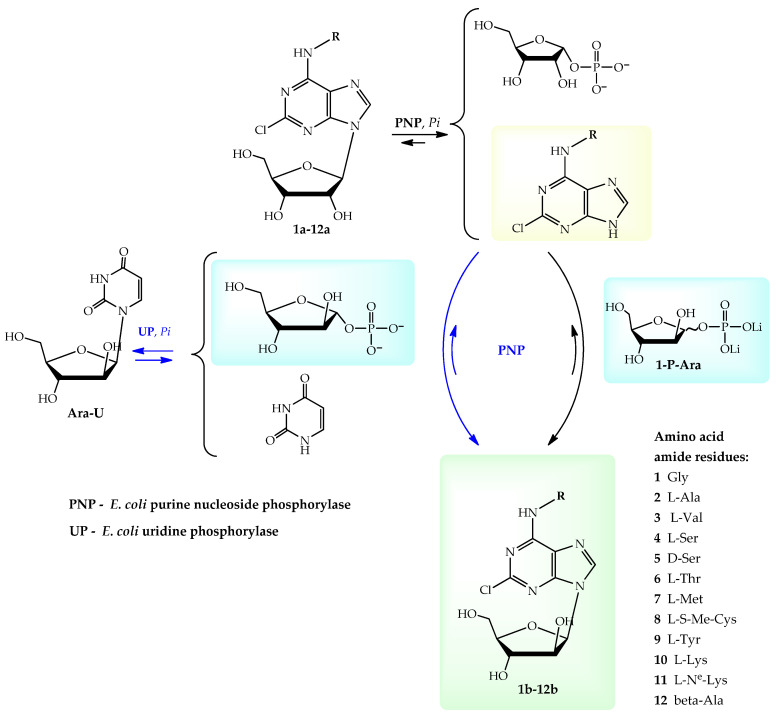
Possible pathways for the synthesis of arabinosides **1b**–**12b**. Two approaches to carrying out the transglycosylation reaction: using synthetic arabinose 1-phosphate (1-P-Ara) (black route) [34] or 1-b-D-arabinofuranosyluracil (Ara-U) as an arabinose donor (azure route).

We recently published an article describing the synthesis of purine ribosides **1a**–**12a** (Scheme 1) modified with chiral amino acid amides at the C6 position of the purine [35]. We showed that derivatives with tyrosine, valine, and serine residues exhibit the properties of A_1_ adenosine receptor partial agonists. The ribosides **1a**–**12a** became the starting compounds for the enzymatic synthesis of arabinonucleosides (Scheme 1).

The synthesis of arabinosides was carried out using enzymatic transglycosylation with recombinant *E. coli* nucleoside phosphorylases [34,36]. Nucleosides substituted at the C6 position of purine are known to be good substrates for *E. coli* purine nucleoside phosphorylase (PNP) [37].

The transglycosylation reaction can be carried out in two ways: using synthetic arabinose 1-phosphate (1-P-Ara) (Scheme 1) [34] or 1-b-D-arabinofuranosyluracil (Ara-U) as an arabinose donor (Scheme 1, Table 1). In the first case, PNP is used (Scheme 1, black route). In the second case, PNP and uridine phosphorylase (UP) are used [38] (Scheme 1, azure route). Ara-U can be easily synthesized from uridine via 2,2′-anhydrouridine according to the method of I. Wempen [39].

**Figure 3:**
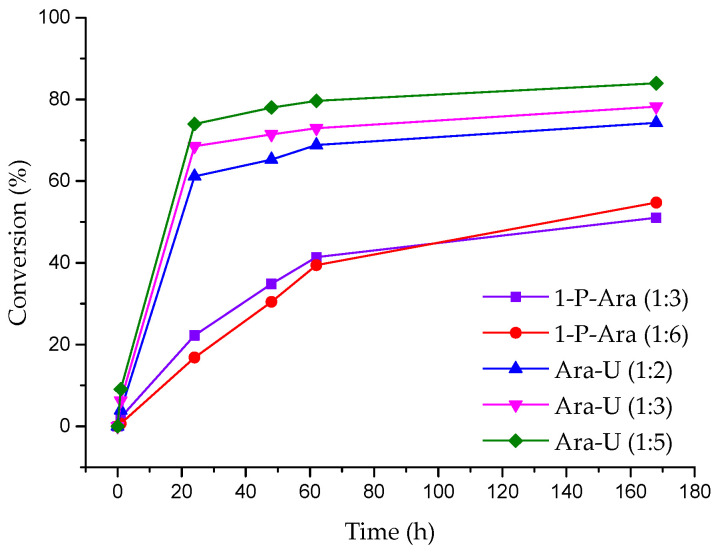
The dependence of the conversion of riboside **1a** to arabinoside **1b** on the type and amount of arabinose donor. The 1 mL reaction mixtures contained 1 mM modified riboside **1a**; 3 or 6 mM 1-P-Ara or 2, 3, or 5 mM Ara-U, 3.9 units PNP, 4.5 units UP in 2 mM potassium-phosphate buffer (pH 7.0).

Figure 3 shows the conversion of riboside **1a** to arabinoside **1b**. Ara-U turned out to be the best arabinose donor.

According to HPLC data, the concentration of the product in the reaction mixture reached 82%, with a fivefold excess of Ara-U (green trend line). The conversion of riboside to arabinoside was 90% in 5 days. Syntheses of nucleosides **1b**–**12b** were carried out using Ara-U at its fivefold molar excess.

As a result of the optimization of enzymatic synthesis, the following conditions for obtaining arabinosides were chosen: the ratio of riboside substrates to Ara-U was 1:5; 0.80 units PNP per 1 mmol of substrate and 0.18 units UP per 1 µmol Ara-U; pH 7.0, 52 °C.

**Figure 4:**
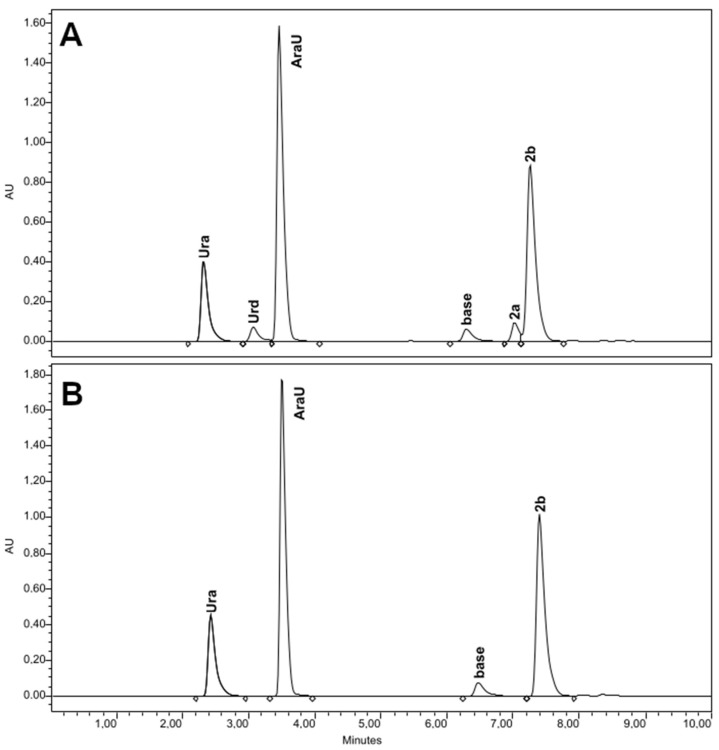
HPLC-profiles of the reaction mixture for the synthesis of an alanine derivative **2b** without arsenate (**A**) and with sodium arsenate (**B**).

During the experiments, we found that the retention times of ribosides and arabinosides were very close (the HPLC profile of the reaction mixture for the synthesis of 6-N-[L-alanylamido]-2-chloro-9-β-D-arabinofuranosylpurine **2b** is shown on Figure 4A, Table 2). In addition to the target arabinoside, the reaction mixture contains up to 8% of the starting riboside and a heterocyclic purine base. This made it difficult to isolate the target product.

To decrease the number of components in the reaction mixture, we decided to employ the arsenolysis of ribosides. It is known that PNP catalyzes the reversible riboside phosphorolysis with the formation of α-D-ribose-1-phosphate.

**Scheme 2:**
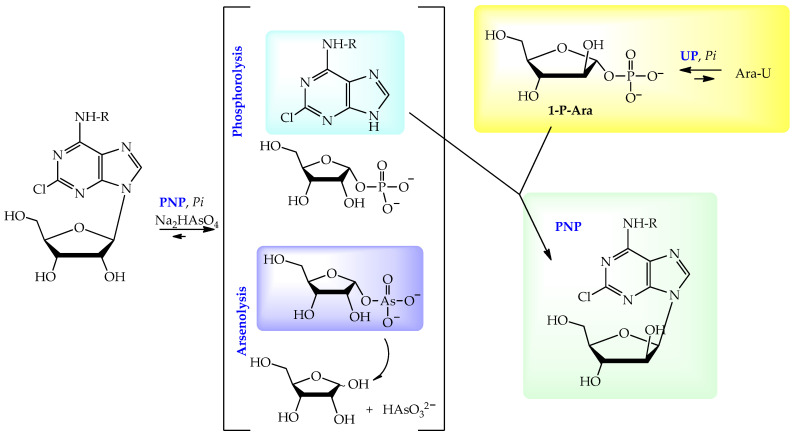
Arsenolysis in the synthesis of arabinosides.

In the presence of arsenates in the active site of PNP, the intermediate α-D-ribose-1-arsenate is formed from the riboside. Being extremely unstable, it rapidly hydrolyzes to ribose and inorganic arsenate, making the reaction irreversible (Scheme 2) [40,41,42].

According to the HPLC data, the number of components in the reaction mixture decreases from six to four when using arsenolysis (Figure 4B, Table 2), and the isolation of target products is facilitated to a significant degree. We used the usual column chromatography instead of preparative HPLC to isolate the target nucleosides.

**Figure 5:**
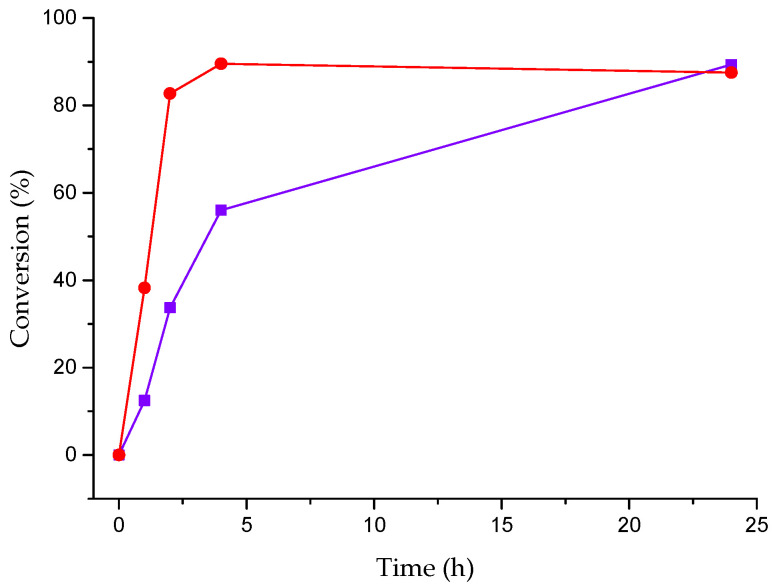
The dependence of the arabinoside **10b** synthesis rate on the moment of arsenate addition. Red line: arsenate was added to the reaction at the start of synthesis. Violet line: arsenate was added to the reaction 24 h after the start of synthesis.

The efficiency of arabinoside synthesis does not change depending on whether the arsenate was added at the beginning of the synthesis or upon reaching the equilibrium state. For example, when arsenate was added at the beginning of the synthesis of methionine arabinoside **10b** in the first 10 h, the equilibrium shifted towards the formation of arabinoside (Figure 5). However, over time (24 h), both reactions achieved the same equilibrium state. The use of arsenate helps to obtain a higher conversion of the riboside in a shorter period of time.

Previously, we showed that the competitive arsenolysis reaction removes ribose from the reaction sphere [43]. When a catalytic amount of sodium arsenate (up to 0.5 mM) is added, arabinose arsenate is essentially not formed in the reaction mixture. The modified arabinose nucleosides hardly undergo arsenolysis (<0.5% in 24 h at 50 °C). As a result, the formation of arabinosides in the reaction mixture prevails.

The conditions of enzymatic reactions for the production of nucleosides **1b**–**12b** are shown in Table 3. The physicochemical characteristics of all the synthesized arabinosides are given in Table 4. NMR spectra and HPLC-profiles are provided in the Supporting Information (Figures S1–S61).

To establish the structure–activity relationship in the biological experiments, we synthesized six nucleosides: 9-β-D-arabinofuranosyl-2-amino-6-(N^α^-glycinyl)-purine (**13**), 9-β-D-arabinofuranosyl-2-amino-6-(N^α^-glycinylamido)-purine (**14**), 2-chloradenosine (**15**), 2-chloro-arabinoadenosine (**16**), 2-chloro-6-O-methyl-(9-β-D-arabinofuranosyl)guanine (Cl-Nelarabine, **17**), and 2-fluoro-6-O-methyl-(9-β-D-arabinofuranosyl)guanine (F-Nelarabine, **18**).

Detailed procedures for the synthesis of **13** and **14** are provided in the Supporting Information. Detailed procedures for the synthesis of 2-aminopurine arabinosides **13** and **14** are provided in the Supporting Information. Nucleosides **15** [25] and **16** [44] (Scheme SI-1 in Supporting Information.), **17**, and **18** [45] were synthesized according to previously developed procedures. NMR spectra and HPLC-profiles are provided in the Supporting Information (Figures S62–S74).

### 2.2. The ADA Substrate Specificity

**Scheme 3:**
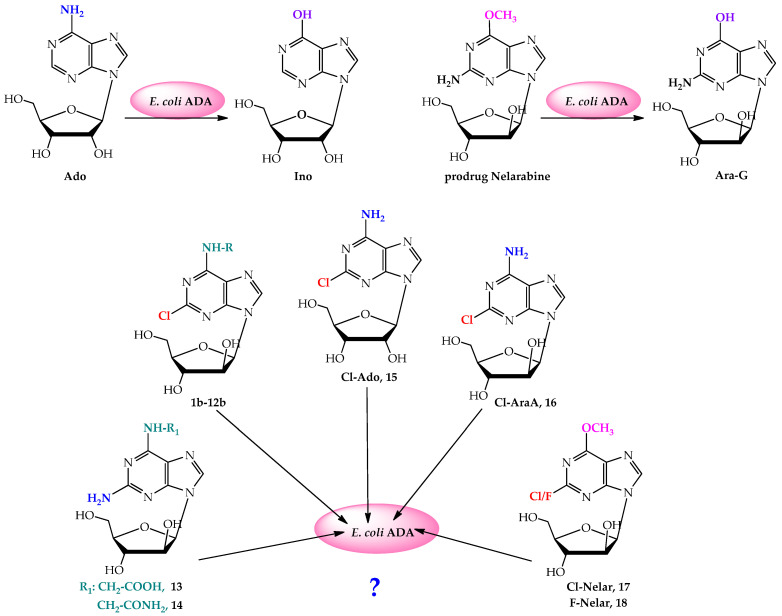
Testing the substrate specificity of *E. coli* ADA towards the synthesized nucleosides.

The resistance of the obtained compounds to the action of *E. coli* adenosine deaminase was evaluated (Scheme 3). All arabinonucleosides **1–18** were resistant to bacterial ADA.

**Figure 6:**
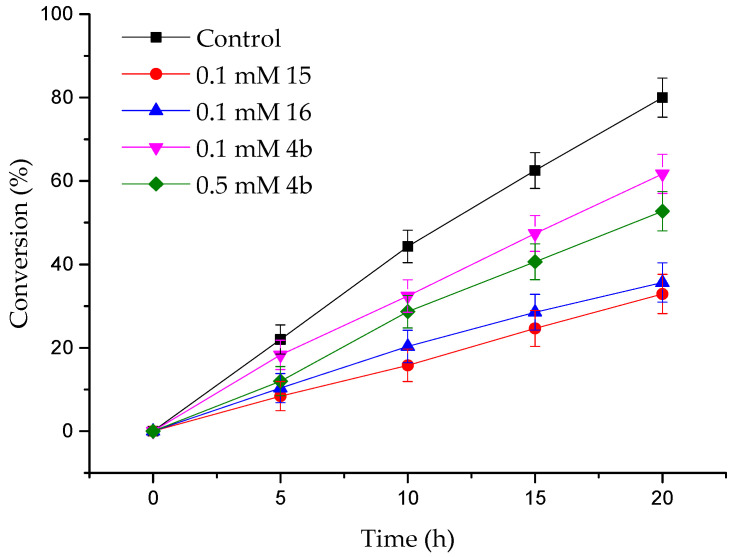
The rate of adenosine deamination in the presence of the nucleosides **15**, **16**, and **4b** (control—adenosine). All reactions were performed in 1 mL 20 mM potassium phosphate (pH 7.0) at 25 °C. The reaction progress was monitored by HPLC. Reaction mixtures contained adenosine (0.26 mg, 0.1 mmol), **15**, **16** (0.3 mg, 0.1 mmol), or **4b** (0.38 mg, 0.1 mmol) and 0.23 units of recombinant *E. coli* ADA. The measurement error did not exceed 5%.

The inhibition of adenosine deaminase by the obtained compounds was studied. Adenosine was used as a control compound for ADA testing. Then, adenosine was deaminated in the presence of synthesized nucleosides. The synthesized compounds at 0.1 mM concentration had little effect on the enzymatic activity of ADA. These reduced the rate of adenosine deamination by less than 10%. Nucleosides **4b**, **15**, and **16** reduced the rate of adenosine deamination under the action of ADA by 23%, 63%, and 54%, respectively. The effect of 2-chloradenosine (**15**), 2-chloro-9-β-D-arabinofuranosyladenine (**16**), and L-seryl arabinofuranoside **4b** on ADA activity is shown in Figure 6.

2-Cloroadenosine appeared to be the best inhibitor among the tested compounds. When ribose was replaced by arabinose (compound **16**), Ki increased by 1.7 times (Table 5). The introduction of the serine fragment at the C6 position of purine (compound **4b**) increased Ki 9.2 times. The compounds **15**, **16**, and **4b** are competitive inhibitors. Lineweaver–Burk plots for the adenosine deamination at various concentrations of **15**, **16,** and compound **4b** are presented in Figure S75. 

### 2.3. The Influence of the Synthesized Nucleosides on U937 Cell Survival

The activity of the synthesized compounds was tested on the U937 cell line. Atrians (9-β-D-arabinofuranosyl-6-O-methylguanine, Nelarabine, GlaxoSmithKline) was used as a reference drug. Atrians is used to treat recurrent and stable T-cell acute lymphoblastic leukemia.

The activity of the compounds was evaluated using IC_50_. The IC_50_ is commonly used to compare the effects of cytotoxic compounds on cell lines. This index determines both the effect on cell death and proliferation during incubation.

**Figure 7:**
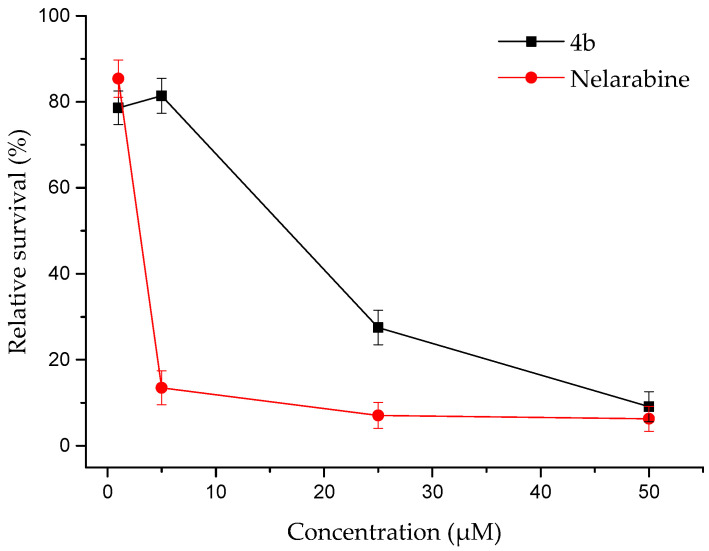
Dependence of U937 cell survival on the concentration of Nelarabine and **4b**.

9-β-D-Arabinofuranosyl-2-chloro-6-(N^α^-L-serinylamido)-purine **4b** has shown inhibitory activity comparable to Nelarabine. Dose–response curves for Nelarabine (IC_50_ = 3 μM) and compound **4b** (IC_50_ = 16 μM) are shown in Figure 7. For both compounds, the effect depended on the concentration in the range of 0.1–50 μM.

Among all the studied compounds, only nucleoside **4b** and, to a lesser extent, 2-chloroadenosine **15** exhibited antiproliferative activity. The mechanism of antiproliferative activity is not entirely clear, since compound **4b** is not a substrate for bacterial ADA, while compound **15** is a competitive ADA inhibitor.

In addition, the chlorine/fluorine atom at the C2 position of purine prevents nucleosides from binding to the ADA active site. Testing of Cl-Nelarabine and F-Nelarabine showed the absence of both the ADA-substrate and antitumor properties (IC_50_ > 50 μM).

Both glycine arabinofuranosides of 2-chloroadenine **1b** and 2-aminoadenine **14** are non-substrates of bacterial ADA. The introduction of an amino acid amide at the C6 position of purine makes nucleosides resistant to adenosine deaminase.

A number of purine arabinosides containing chiral amino acid amides at the C6 position of the purine **1b**–**12b** did not exhibit antiproliferative activity (IC_50_ over 50 μM) (Table 6). A correlation was found between the lack of ADA substrate properties of the arabinosides synthesized and low anti-tumor activity against human T-lymphoblastic leukemia cells. ADA cannot replace the amino acid residue at position C6 with a hydroxyl to form an analog of the active Ara-G molecule. It can be concluded that the antiproliferative effect of compound **4b** does not depend on the action of intracellular ADA. Thus, the mechanism of antiproliferative activity of the serine analog **4b** differs from that of Nelarabine.

The antiproliferative activity of the compounds synthesized was tested on other tumor cell lines: LS174T human Caucasian colon adenocarcinoma, SKOV3 ovarian cancer cells, MCF7 breast cancer cells, and A549 non-small-cell lung cancer cells. None of the compounds tested affected cell viability: survival was >80% (within concentrations up to 50.00 μM).

## 3. Materials and Methods

### 3.1. General Procedures

All chemicals and solvents were of laboratory grade, were obtained from commercial suppliers, and were used without further purification. The method of producing of recombinant *E. coli* phosphorylases was described above. The following recombinant *E. coli* [36] enzymes were used in the present study: UP with specific activity 100 units per mg of protein, 9 mg per mL; PNP 52 units per mg, 15 mg per mL. Ara-U was synthesized as described in [34]. 1-P-Ara was synthesized as described in [46]. Analytical HPLC was performed using the Waters system (Waters 1525, Waters 2489, Breeze 2, (Waters Inc., Milford, MA, USA); (a) Nova Pak C18, 4.6 × 150 mm, eluent A—0.1% TFA/H_2_O, eluent B—70% acetonitrile in 0.1% TFA/H_2_O, flow rate 1 mL/min, detection at 280 nm. Gradient 0–100% B, 20 min; (b) Ascentis^®^ Express C18, 2.7 μm 7.5 × 3.0 mm, eluent A—0.1% TFA/H_2_O, eluent B—70% acetonitrile in 0.1% TFA/H_2_O, flow rate 0.5 mL/min, detection at 280 nm. Gradient 0–100% B, 20 min.

NMR spectra were recorded using a Bruker Avance II 700 spectrometer (Bruker BioSpin, Rheinstetten, Germany) in DMSO-*d*_6_ at 30 °C. Chemical shifts in ppm (δ) were measured relative to the residual solvent signals as internal standards (2.50). Coupling constants (*J*) were measured in Hz.

Liquid chromatography mass spectrometry was performed using an Agilent 6210 TOF LC/MS system (Agilent Technologies, Santa Clara, CA, USA). UV-Spectra were recorded using a Hitachi U-2900 spectrophotometer (Tokyo, Japan).

The optical rotations were measured for purified samples using the digital polarimeter JASCO model DIP-370 (cylindrical glass cell 3.5 mm D × 50 mm) (Tokyo, Japan).

### 3.2. Enzymatic Reactions

The reaction conditions of arabinosides synthesis (ratio of reagents, PNP amount, reaction time, and conversion of the base into nucleoside) are provided in Table 1. Riboside (**1a**–**12a**), KH_2_PO_4_, and Ara-U were dissolved in water at 40–50 °C, the pH was adjusted up to 7.0, and Na_2_HAsO_4_ was added to the reaction mixture.

The enzymes (PNP, UP) were added and the reaction mixtures were incubated at 50 °C. The reaction progress was monitored by HPLC. When conversion reached the highest value, the reaction was terminated by addition of ethanol (50%, *v*/*v*). The reaction mixtures were evaporated up to 5 mL, and the desired products **1b**–**12b** were isolated by reversed-phase column chromatography (silica gel C18, Merck), column 100 × 20 mm (the elution conditions are shown in Table 1). The physicochemical and spectral properties of nucleosides are reported in Table 3. The NMR spectra and HPLC of nucleosides are provided in the Supporting Information.

### 3.3. The ADA Substrate Specificity

The substrate specificity of *E. coli* adenosine deaminase toward arabinosides **1b**–**12b** was determined using a previously published method [47]. According to the HPLC data, the obtained nucleosides do not undergo deamination in vitro.

### 3.4. Inhibition of E. coli ADA

The reaction mixtures contained 0.04 mM adenosine (Serva), 5 mM potassium phosphate (pH 7.0), and 0.1 mM inhibitor: 6-Amino-2-chloropurine riboside (2-chloroadenosine), 6-Amino-2-chloro-9-beta-D-arabinofuranosyl-9H-purine (2-Cl-AraA), or compound **4b**. ADA (0.008 units) was added and the reaction mixtures were incubated at 25 °C for 20 min. The conversion of adenosine into inosine was monitored every 5 min by HPLC.

### 3.5. Biological Assay

For the experiments, all compounds were dissolved in DMSO and then brought to the desired concentration with an RPMI 1640 nutrient medium. The final concentration of DMSO in the samples did not exceed 0.1% and did not affect cell growth.

The antiproliferative activity of the compounds synthesized was tested on tumor cell lines: U937, LS174T, SKOV3, MCF7, and A549 cancer cells. The cells lines were kindly provided by the Tumor Strain Bank of the N. N. Blokhin Cancer Research Center. The cells were grown in RPMI1640 medium containing 2 mM glutamine and 10% fetal calf (FCS) serum at 37 °C and 5% CO_2_.

Influence on cell growth was evaluated according to the cell survival rate in the presence of compounds in the studied concentrations. Cells were seeded in 96-well flat-bottom microplates at a seeding density of 8000 cells/well. The test compounds were added to the wells in a volume of 20 µL. The total incubation volume was 200 μL. The incubation time with nucleosides was 48 h (96 h for U937). At the end of the incubation, the MTT reagent was added to the cells and the cells were incubated for 2 h.

The formed formazan crystals were dissolved in 100 µL of DMSO at 37 °C for 20 min. The absorbance of DMSO solutions was measured on an optical counter for multiwell plates at λ = 540 nm.

Cell survival for the corresponding concentration was calculated using the following formula: Ti/C × 100%, where Ti is the OD after incubation with the test agents and C is the OD of parallel control samples (without test compounds). A compound was considered active if the concentration of it that caused growth inhibition by 50% (IC_50_ calculated from the dose–response curve) was equal to or less than IC_50_ for Nelarabine. The measurement error did not exceed 5%.

The results were expressed as averages for 4 parallel measurements in 2–3 experiments as cell survival in % (experiment/control) ×100.

Statistical processing of the obtained results was carried out using the Microsoft Excel 2010 program with the method of variational statistics with the determination of the arithmetic mean and the error of the mean (M ± m). The differences between values were considered significant at *p* < 0.05.

## 4. Conclusions

A series of purine arabinosides (12 compounds) containing chiral amino acid amides at the C6 position of the purine was synthesized using a transglycosylation reaction. To establish the structure–activity relationship in the biological experiments, six nucleosides were additionally synthesized: 9-β-D-ribofuranosyl-2-amino-6-(N^α^-glycinyl)-purine, 9-β-D-ribofuranosyl-2-amino-6-(N^α^-glycinylamido)-purine, 2-chloradenosine, 2-chloro-arabinoadenosine, 2-chloro-6-O-methyl-(9-β-D-arabinofuranosyl)guanine (Cl-Nelarabine), and 2-fluoro-6-O-methyl-(9-β-D-arabinofuranosyl)guanine (F-Nelarabine).

Arsenolysis of 2-chloropurine ribosides with chiral amino acid amides at C6 was used in the enzymatic synthesis to simplify the composition of the reaction mixtures. In the presence of arsenates in the active site of PNP, the unstable intermediate α-D-ribose-1-arsenate was formed from the riboside, but arabinose arsenate was essentially not formed in the reaction mixture. The modified arabinose nucleosides hardly undergo arsenolysis, and the formation of arabinosides in the reaction mixture prevails.

The synthesized nucleosides were shown to be resistant to the action of *E. coli* adenosine deaminase. The antiproliferative activity of synthesized nucleosides was studied on human acute myeloid leukemia cell line U937. Among all compounds, the serine derivative exhibited an activity level (IC_50_ = 16 μM) close to that of Nelarabine (IC_50_ = 3 μM) and was evaluated as active.

## Figures and Tables

**Table 1:** Overall conversion and yield.

Compound	Amino Acid Amide Residue	R	Conversion into Nucleoside, % (HPLC), Azure Route	Nucleoside Yield (%), Azure Route
**1b**	Gly	-CH-CONH_2_	82.88	63
**2b**	L-Ala	-CH(CH_3_)-CONH_2_	81.44	50
**3b**	L-Val	-CH(CH_3_)_2_-CONH_2_	69.15	46
**4b**	L-Ser	-CH(CH_2_OH)-CONH_2_	90.60	81
**5b**	D-Ser	-CH(CH_2_OH)-CONH_2_	78.10	77
**6b**	L-Thr	-CH(CH(OH)CH_3_)-CONH_2_	80.57	60
**7b**	L-Met	-CH(CH_2_-CH_2_-S-CH_3_)-CONH_2_	91.51	88
**8b**	L-S-Me-Cys	-CH(CH_2_-S-CH_3_)-CONH_2_	80.39	75
**9b**	L-Tyr	-CH(CH_2_-C_6_H_4_-OH)-CONH_2_	94.83	68
**10b**	L-Lys	-CH(CH_2_-CH_2_-CH_2_-CH_2_-NH_2_)-CONH_2_	96.85	92
**11b**	N^ε^-Lys	-CH_2_-CH_2_-CH_2_-CH_2_-CH(NH_2_)-CONH_2_	71.76	52
**12b**	β-Ala	-CH_2_-CH_2_-CONH_2_	70.02	63

**Table 2:** Retention time (RT) and peak area of compounds.

Peak Name	Ura	Urd	Ara-U	base	2a	2b
RT (min)	2.312	3.062	3.454	6.285	7.014	7.248
% Area before arsenate adding	13.25	2.84	44.72	3.00	2.87	33.32
% Area after arsenate adding	15.09	-	44.00	3.57	-	37.34

**Table 3:** Experimental data for the enzymatic synthesis of arabinosides **1b**–**12b** ^a^.

Compound	Acceptor mol wt.	Donor mol wt.	Substrates		Reaction Volume, mL	PNP, Units (A) ^b^	UP, Units (B) ^b^	Volume, ml (20 mM Na_2_HAsO_4_)	Reaction Time, h	Conversion of Base into Nucleoside (HPLC Data), %	Eluent ^c^ %
			Acceptor mg (mmol)	Donor mg (mmol)							
**1b**	**1a** 359.09	Ara-U 244.2	75 (0.21)	260 (1.06)	104	162 (776)	94 (88)	0.208	196	82.88	50
**2b**	**2a** 373.10		80 (0.21)	260 (1.06)	106	165 (770)	95 (89)	0.212	196	81.44	50
**3b**	**3a** 401.13		80 (0.20)	244 (1.00)	100	156 (782)	90 (90)	0.200	336	69.15	70
**4b**	**4a** 389.10		80 (0.21)	251 (1.03)	103	161 (783)	93 (90)	0.206	216	90.60	50
**5b**	**5a** 389.10		100 (0.26)	313 (1.28)	128	200 (778)	115 (90)	0.256	504	78.10	50
**6b**	**6a** 403.11		100 (0.25)	300 (1.23)	75	115 (464)	70 (57)	0.150	196	80.57	70
**7b**	**7a** 433.11		34 (0.08)	90 (0.37)	20	133 (1694)	90 (244)	0.040	168	91.51	70
**8b**	**8a** 419.09		40 (0.10)	120 (0.49)	40	61 (639)	41 (83)	0.080	196	80.39	70
**9b**	**9a** 465.13		100 (0.21)	260 (1.06)	60	90 (419)	54 (51)	0.120	196	94.83	70
**10b**	**10a** 430.16		15 (0.03)	41 (0.17)	50	76 (2179)	45 (268)	0.100	168	96.85	70
**11b**	**11a** 430.16		100 (0.23)	286 (1.17)	117	91 (391)	166 (142)	0.234	196	71.76	70
**12b**	**12a** 373.10		80 (0.21)	261 (1.07)	107	167 (779)	96 (90)	0.214	168	70.02	50

^a^ All enzymatic reactions were performed in 5 mM potassium phosphate buffer (pH 7.0) at 50 °C. Ratio Acceptor:Donor—1:5. The reaction progress was monitored by HPLC. The reactions were stopped when the riboside was transformed into a nucleoside, as in the test reactions. Solutions of recombinant *E. coli* UP and PNP in 5 mM potassium phosphate buffer (pH 7.0) with activities of 1700 and 1400 units per mL, respectively, were used [36]. Target products were isolated by column chromatography (C18, 20 × 100 mm). ^b^ A—ratio of UP (units) per Ara-U (1 mmol). B—ratio of PNP (units) per nucleoside **1b**–**12b** (1 mmol). ^c^ Nucleosides were eluted from the C18 column with a water–methanol mixture. Eluent—% (vol).

**Table 4:** Physicochemical properties of arabinonucleosides **1b–12b**.

Compound	Yield (%), (mg)	Purity (%)	RT, min	[α]D^25^	UV λ_max_, nm (ε)	HRMS: m/z Calcd [M+H]^+^	HRMS: m/z Found [M+H]^+^
**1b**	63 (43)	98.69	9.50 ^a^	16.8 (c 0.25, H_2_O/DMSO 1:1)	268 (14,900), 212 (18,800)	227.0442 (base) 359.0865	227.0458 359.0894
**2b**	50 (40)	99.72	7.47 ^a^	40.4 (c 0.5, H_2_O)	270 (15,200) 213 (18,600)	241.0599 (base) 373.1021	241.0592 373.1015
**3b**	46 (37)	96.63	9.24 ^a^	20.8 (c 0.5, H_2_O)	270 (16,800) 213 (18,700)	269.0912 (base) 401.1334	269.0889 401.1335
**4b**	81 (58)	99.27	6.15 ^a^	43.6 (c 0.5, H_2_O)	270 (17,900) 212 (23,600)	257.0548 (base) 389.0971	257.0541 389.1007
**5b**	77 (77)	95.48	5.76 ^a^	−20.4 (c 0.5, H_2_O)	268 (16,600) 212 (20,400)	257.0548 (base) 389.0971	257.0541 389.1007
**6b**	60 (60)	95.75	5.68 ^b^	76.0 (c 1.0, H_2_O/DMSO)	269 (21,000) 213 (24,800)	271.0704 (base) 403.1127	271.0695 403.1107
**7b**	88 (30)	98.20	7.35 ^b^	54.0 (c 1.0, H_2_O/DMSO)	268 (19,400) 212 (23,200)	301.0632 (base) 433.1055	301.0636 433.1061
**8b**	75 (21)	91.20	7.01 ^b^	80.0 (c 1.0, H_2_O/DMSO)	270 (16,700) 212 (18,700)	287.0476 (base) 419.0898	287.0457 419.08703
**9b**	68 (68)	94.38	6.91 ^b^	46.0 (c 1.0, H_2_O/DMSO)	271 (19,200) 213 (25,400)	333.0861 (base) 465.1284	333.0851 465.1269
**10b**	92 (10)	98.42	6.63 ^b^	-	270 (15,300) 213 (17,800)	298.1177 (base) 430.1600	298.1161 430.1581
**11b**	52 (52)	95.60	5.35 ^b^	4.8 (c 1.0, H_2_O/DMSO)	270 (15,400) 213 (17,800)	298.1177 (base) 430.1600	298.1161 430.1581
**12b**	63 (50)	99.13	7.24 ^b^	−4.8 (c 0.25, H_2_O/DMSO 1:1)	270 (16,500) 213 (19,400)	241.0599 base 373.1021	241.0603 373.1043

All nucleosides were obtained as a white amorphous powder. The UV spectra were recorded using a Hitachi U-2900 spectrophotometer. The mass spectra were recorded using an Agilent 6224, ESI-TOF, LC/MS. The optical rotations [α]D^25^ were measured for purified samples using the digital polarimeter JASCO model DIP-370 (cylindrical glass cell 3.5 mm D × 50 mm). HPLC was performed using the Waters system (Waters, Breeze 2); HPLC methods for each compound are given in the Supporting Information. ^a^ Nova Pak C18, 4.6 × 150 mm column, eluent A—0.1% TFA/ H_2_O, eluent B—70% acetonitrile in 0.1% TFA/H_2_O, flow rate 1 mL/min, detection at 280 nm. Gradient 0–100% B, 20 min; ^b^ Ascentis^®^ Express C18. 2.7 μm 7.5 × 3.0 mm, eluent A—0.1% TFA/H_2_O, eluent B—70% acetonitrile in 0.1% TFA/H_2_O, flow rate 0.5 mL/min, detection at 280 nm. Gradient 0–100% B, 20 min.

**Table 5:** Inhibition of *E. coli* adenosine deaminase.

Inhibitor	Ki, mM
2-chloradenosine **15**	0.078 ± 0.012
2-chloro-arabinoadenosine **16**	0.13 ± 0.02
**4b**	1.2 ± 0.2

**Table 6:** Effect of nucleosides on U937 cell survival.

Compound	U937 Cell Survival, %				IC_50_, µM
	1.00 µM	5.00 µM	25.00 µM	50.00 µM	
**1b**	79 ± 2.3	83 ± 2.4	64 ± 3.2	60 ± 3.0	>50
**2b**	73 ± 3.6	90 ± 3.6	71 ± 2.8	68 ± 2.0	>50
**3b**	81 ± 3.2	85 ± 2.5	69 ± 2.7	65 ± 1.9	>50
**4b**	78 ± 3.1	81 ± 4.0	27 ± 1.1	9 ± 0.2	16.0
**5b**	80 ± 4.0	76 ± 3.8	58 ± 2.3	48 ± 1.4	50
**6b**	98 ± 4.9	90 ± 2.7	85 ± 2.5	79 ± 2.3	>50
**7b**	100 ± 4.0	105 ± 5.2	90 ± 3.6	76 ± 2.2	>50
**8b**	85 ± 3.4	83 ± 2.4	70 ± 2.1	56 ± 1.7	≈50
**9b**	88 ± 4.4	82 ± 4.1	76 ± 2.3	70 ± 2.8	>50
**10b**	74 ± 2.2	71 ± 2.1	69 ± 3.4	61 ± 1.8	>50
**11b**	84 ± 4.2	89 ± 4.4	84 ± 4.2	79 ± 2.3	>50
**12b**	100 ± 4.0	82 ± 3.2	71 ± 2.1	69 ± 2.0	>50
**13**	98 ± 4.0	87 ± 2.6	75 ± 3.0	63 ± 1.8	>50
**14**	94 ± 2.8	77 ± 2.3	69 ± 3.4	50 ± 2.0	50
**15**	83 ± 3.3	80 ± 2.4	47 ± 1.9	27 ± 1.1	22.0
**17**	92 ± 3.6	88 ± 4.4	80 ± 4.0	73 ± 2.1	>50
**18**	98 ± 3.9	93 ± 3.7	84 ± 2.5	76 ± 3.8	>50
Nelarabine	85 ± 2.5	13 ± 0.6	7 ± 0.3	6 ± 0.1	3.3

## Data Availability

Data are contained within the article or in the Supplementary Materials.

## Supplementary Materials

The following supporting information can be downloaded at: https://www.mdpi.com/article/10.3390/ijms24076223/s1.
