# Supplementary material for: Enzymatic Synthesis of 2-Chloropurine Arabinonucleosides with Chiral Amino Acid Amides at the C6 Position and an Evaluation of Antiproliferative Activity In Vitro
Source: Int J Mol Sci. 2023 Mar 25;24(7):6223. doi: 10.3390/ijms24076223 (PMC10094600; doi:10.3390/ijms24076223)
Supplement: Supplementary file 1 [file ijms-24-06223-s001.zip › ijms-2279884-supplementary.pdf]

Supporting Information

Enzymatic Synthesis of 2-Chloropurine Arabinonucleosides with Chiral Amino Acid Amides at the C6 Position and an Evaluation of Antiproliferative Activity In Vitro

Barbara Z. Eletskaya [1,*], Maria Ya. Berzina [1], Ilya V. Fateev[1], Alexei L. Kayushin [1], Elena V. Dorofeeva [1], Olga I. Lutonina [1], Ekaterina A. Zorina [1], Konstantin V. Antonov [1], Alexander S. Paramonov [1], Inessa S. Muzyka [1], Olga S. Zhukova [2], Mikhail V. Kiselevskiy [2], Anatoly I. Miroshnikov [1], Roman S. Esipov [1], and Irina D. Konstantinova [1,*]

1 Shemyakin and Ovchinnikov Institute of Bioorganic Chemistry, Russian Academy of Sciences, Miklukho-Maklaya St. 16/10, 117997 Moscow, Russia
2 State N.N. Blokhin Russian Cancer Research Center, Kashirsky Highway, 24, 115478, Moscow, Russia

* Correspondence: fraubarusya@gmail.com (B.Z.E.); kid1968@yandex.ru (I.D.K.)

Content	Page number
NMR spectra and HPLC data of compounds **1b-12b**.	2-47
Synthesis and NMR spectra and HPLC data of compounds **15** and **16**	48-52
Synthesis and NMR spectra and HPLC data of arabinosides **13** and **14**	53-59
Lineweaver–Burk plots for the adenosine deamination	60

NMR spectra and HPLC data of compounds 1b-12b.

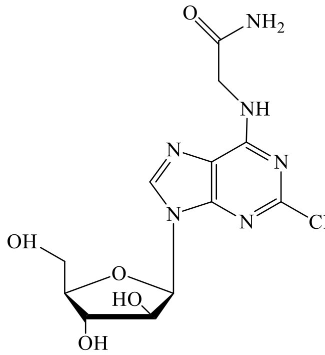

1b

9-β-D-arabinofuranosyl-2-chloro-6-(N$^{\alpha}$-glycinylamido)-purine (1b)

^{1}H NMR (700 MHz, DMSO-d_6, 30 °C): δ= 8.25 and 8.22 (2 s, 0.60H and 0.16H, H-8), 8.12 and 8.01 (2 s, 0.78H and 0.17H, C6-NH), 7.41 and 7.05 (2 sign., 1H, CO-NH), 7.41 and 7.01 (2 sign., 1H, CO-NH), 6.17 (d, J = 5.2 Hz, 0.81H, H-1'), 5.64 (br.s., 0.90H, 2'-OH), 5.52 (s, 1H, 3'-OH), 5.05 (s, 0.89H, 5'-OH), 4.19 (m, 1H, H-2'), 4.13 (m, 1H, H-3'), 3.99 (br.sign., 1.65H, NH-C$\underline{H}_2$), 3.79 (m, 1H, H-4'), 3.68 (m, 1H, H-5'a), 3.65 (m, 1H, H-5'b) ppm.

^{13}C NMR (176 MHz, DMSO-d_6, 30 °C): δ= 140.39 (C8), 83.36 (C1'), 83.75 (C4'), 75.22 (C2'), 74.29 (C3'), 60.30 (C5'), 42.43 (NH-CH_2) ppm.

^{15}N NMR (71 MHz, DMSO-d_6, 30 °C): δ= 239.30 (N7), 169.16 (N9), 103.67 (CO-NH_2), 85.17 (C6-NH) ppm.

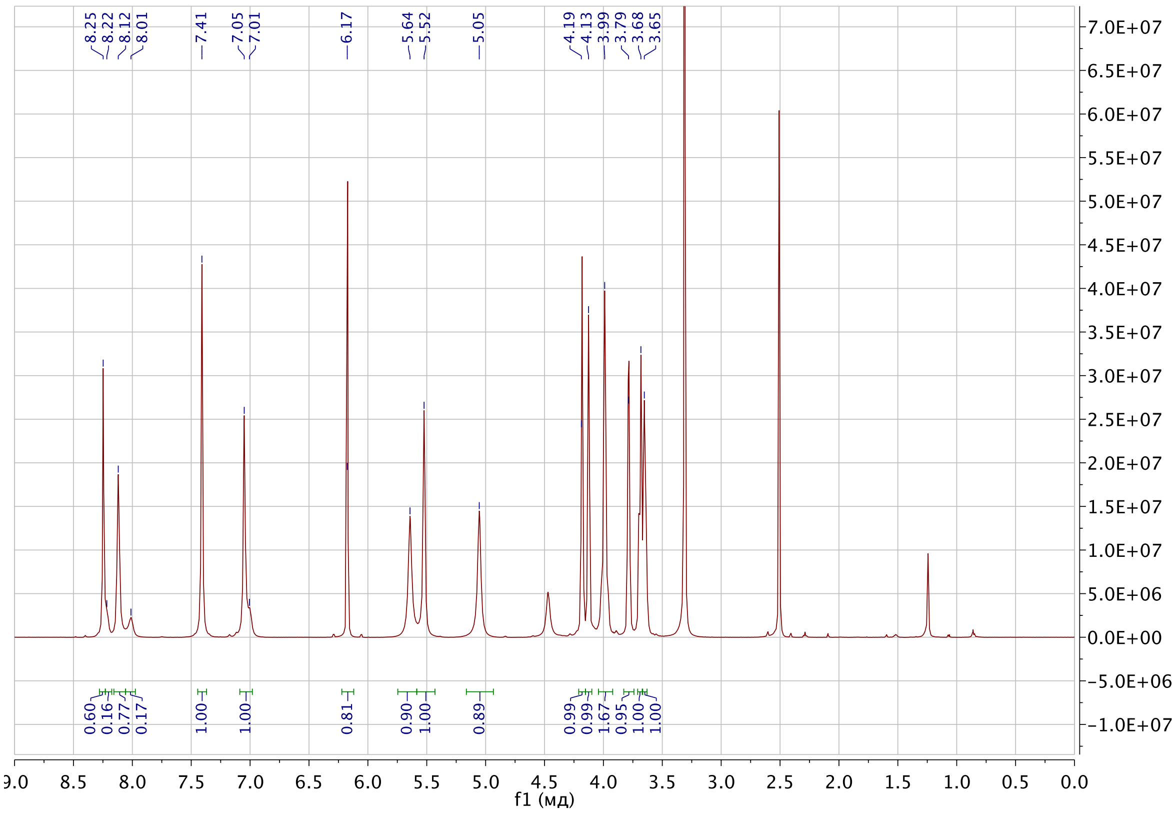

Figure SI-1. The 1H NMR spectrum of 9-β-D-arabinofuranosyl-2-chloro-6-(N$^{\alpha}$-glycinylamido)-purine (1b)

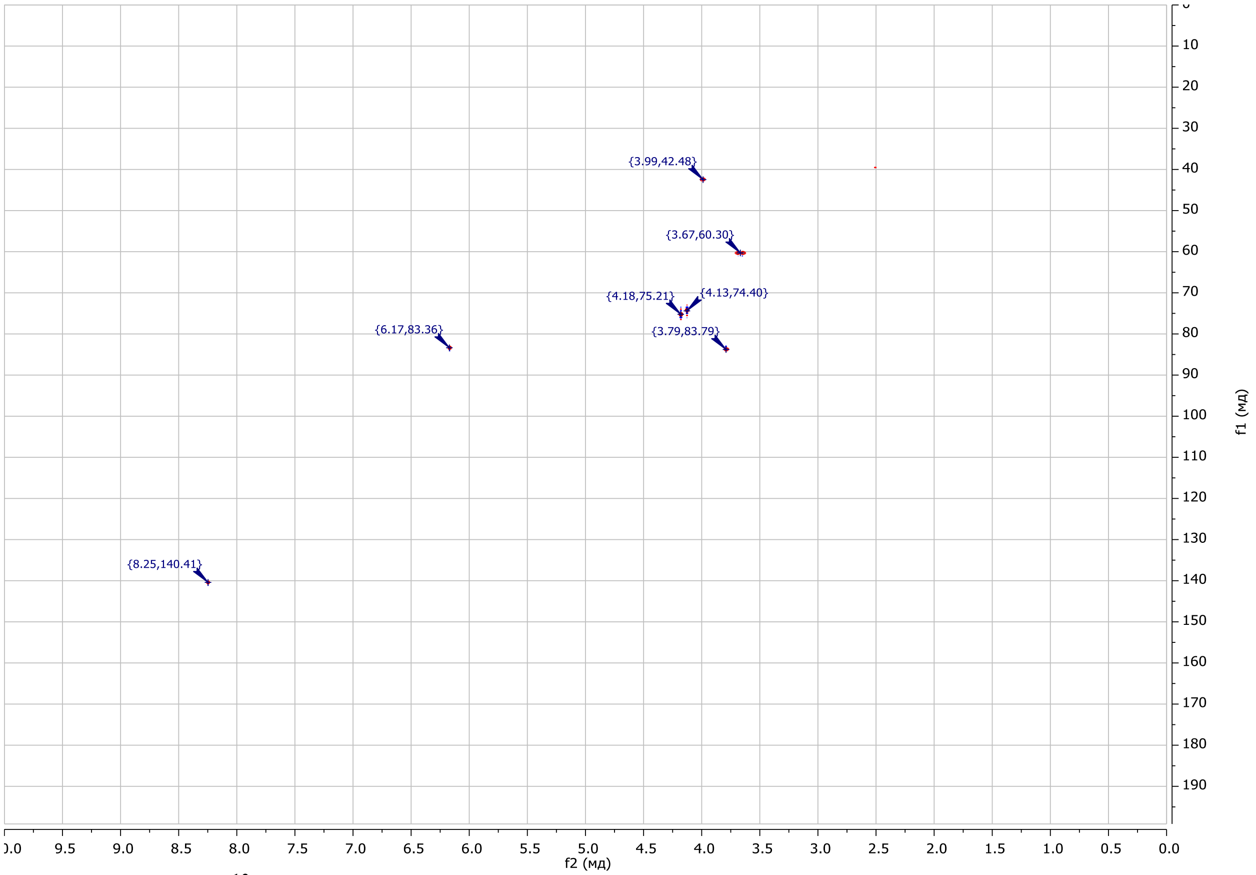

Figure SI-2. The ^{13}C HSQC NMR spectrum of 9-*β*-*D*-arabinofuranosyl-2-chloro-6-(N$^{\alpha}$-glycinylamido)-purine (**1b**)

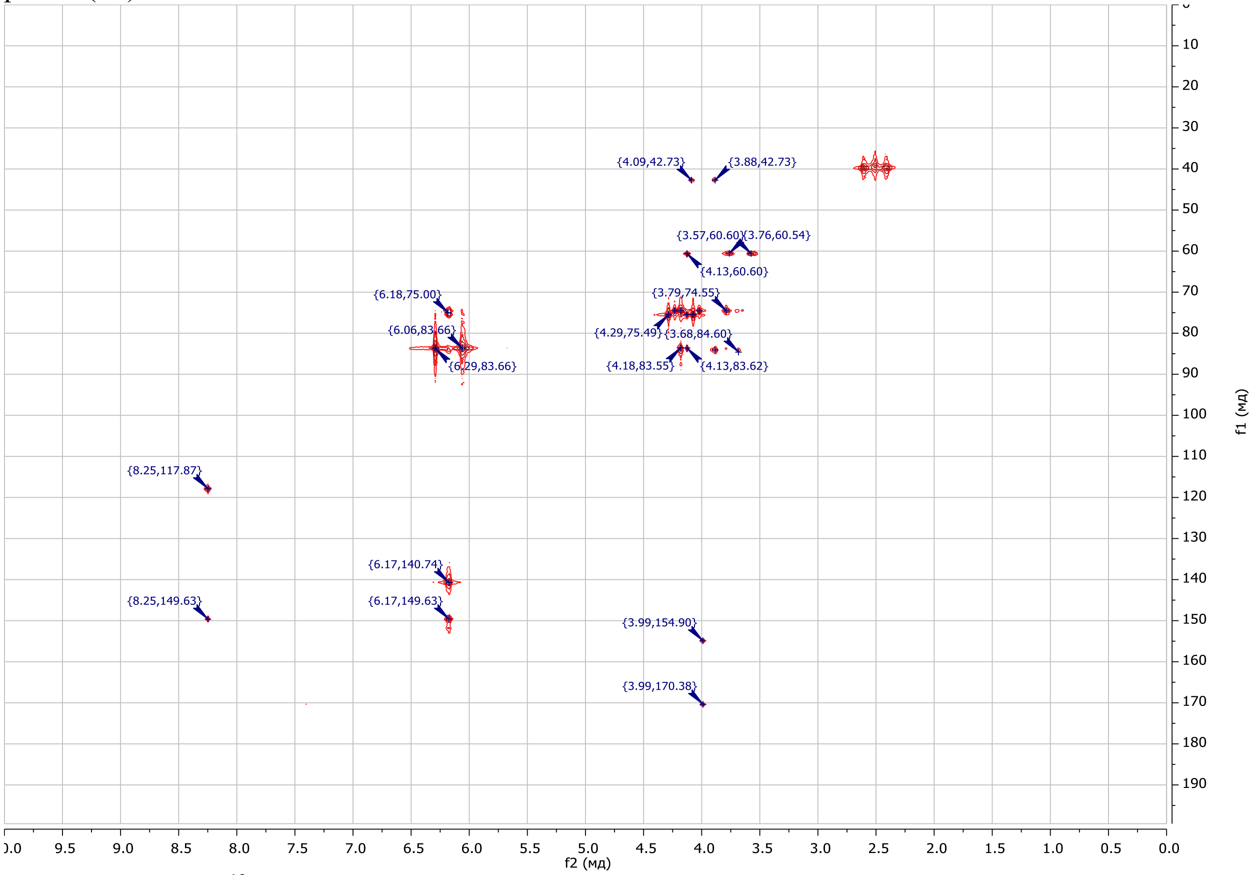

Figure SI-3. The ^{13}C HMBC NMR spectrum of 9-*β*-*D*-arabinofuranosyl-2-chloro-6-(N$^{\alpha}$-glycinylamido)-purine (**1b**)

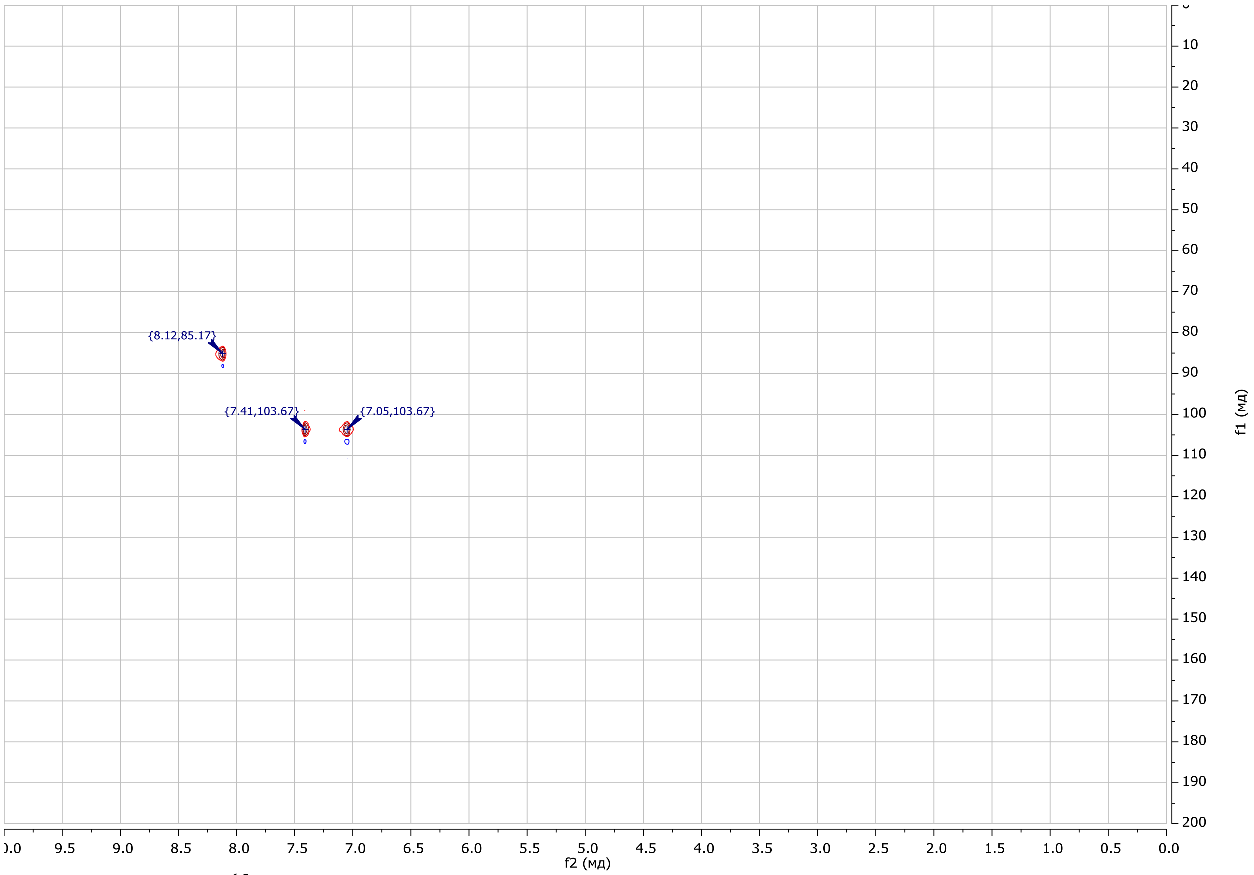

Figure SI-4. The ^{15}N HSQC NMR spectrum of 9-*β*-*D*-arabinofuranosyl-2-chloro-6-(N$^{\alpha}$-glycinylamido)-purine (**1b**)

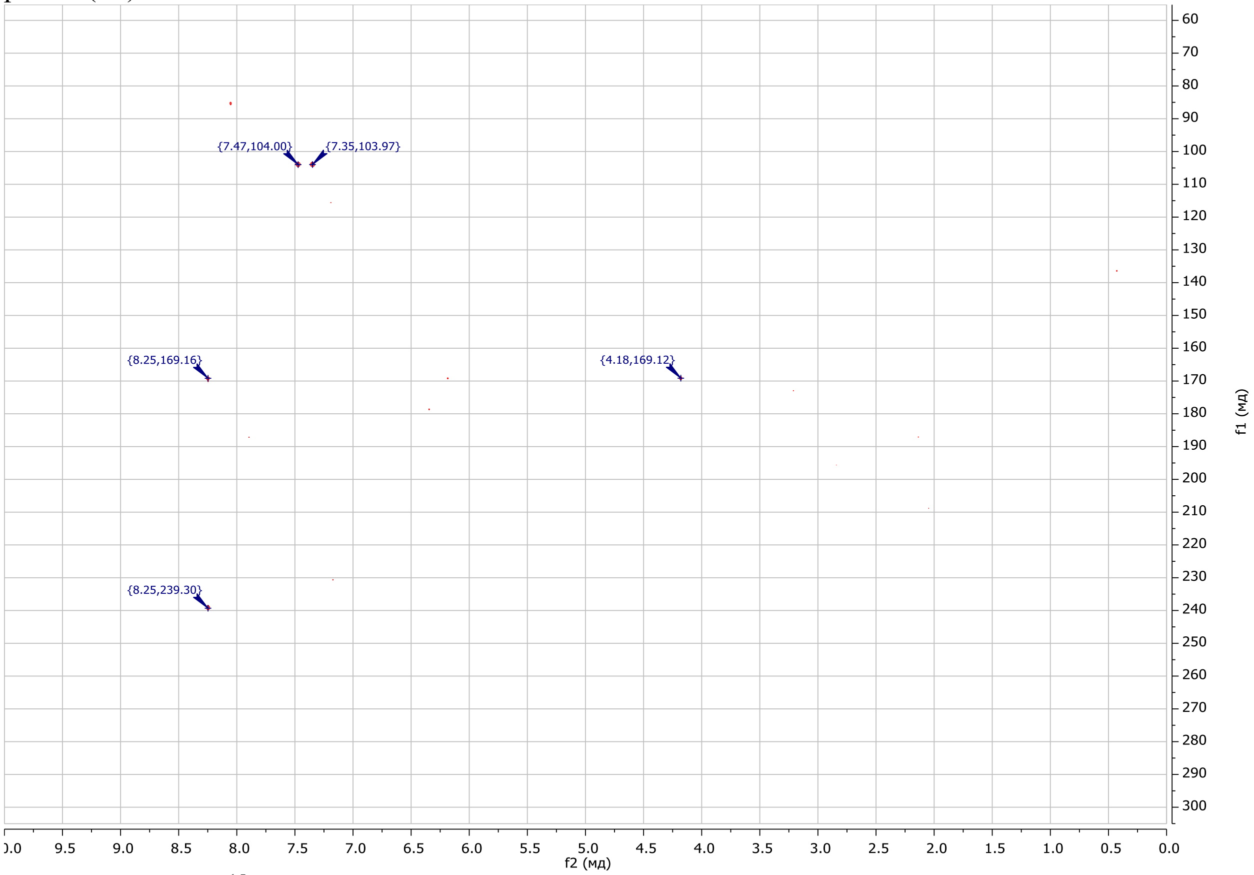

Figure SI-5. The ^{15}N HMBC NMR spectrum of 9-*β*-*D*-arabinofuranosyl-2-chloro-6-(N$^{\alpha}$-glycinylamido)-purine (**1b**)

BT

Project Name: TESTLast_1

Reported by User: System

SAMPLE INFORMATION

Sample Name:	1b	Acquired By:	System
Sample Type:	Unknown	Date Acquired:	12.05.2014 11:01:08
Vial:	1	Acq. Method:	100B_dual_280
Injection #:	1	Date Processed:	19.05.2014 12:44:26
Injection Volume:	5,00 ul	Channel Name:	2487Channel 2
Run Time:	20,00 Minutes	Sample Set Name:	
Column Type:			

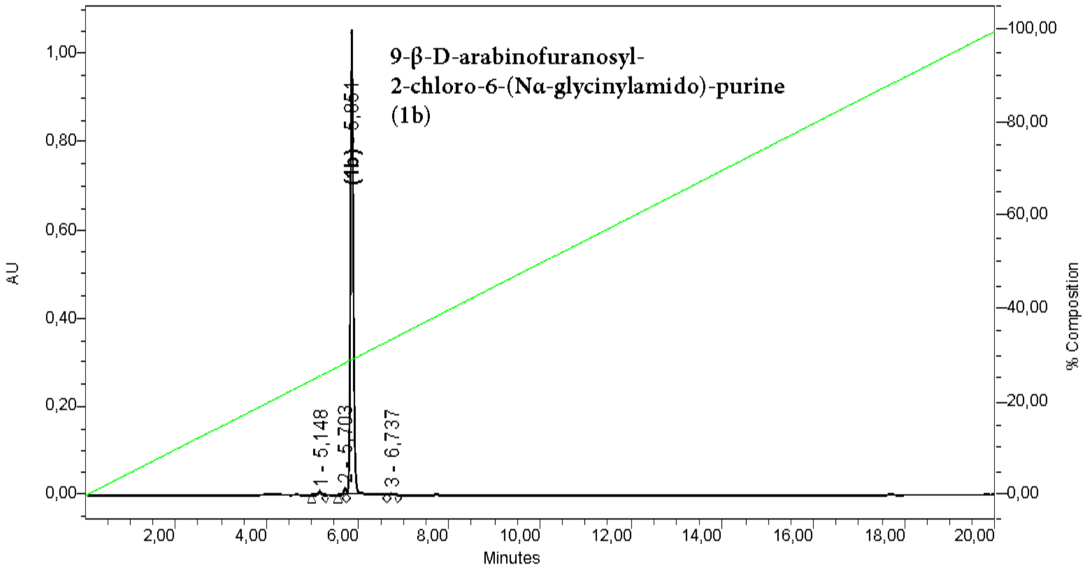

	Peak Name	RT (min)	Area (µV*sec)	% Area	Height (µV)	% Height
1	1	5,148	41892	0,88	7514	0,72
2	2	5,703	68452	1,44	15749	1,50
3	**1b**	5,851	4623441	97,23	1021351	97,45
4	3	6,737	21212	0,45	3447	0,33

Report Method: Gradient Overlay Report ASC Printed 12:44:33 19.05.2014 Page: 1 of 1

Figure SI-6. The chromatogram of **1b**

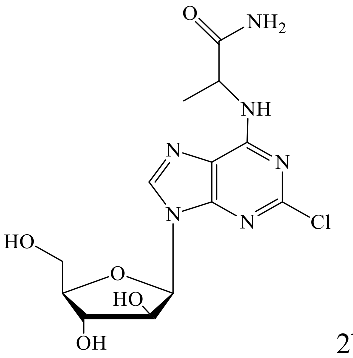

2b

9-β-D-arabinofuranosyl-2-chloro-6-(N$^{\alpha}$-L-alaninylamido)-purine (2b)

^{1}H NMR (700 MHz, DMSO-d_6, 30 °C): δ= 8.25(1 s, 1H, H-8), 7.87 and 7.86 (br.d., J = 6.0 Hz, 1 H, C6-NH), 7.44 and 7.08 (2 sign., 1H, CO-NH), 7.44 and 7.05 (2 sign., 1H, CO-NH), 6.17 (d, J = 4.7 Hz, 1H, H-1'), 5.63 (d., J = 4.7 Hz, 1H, 2'-OH), 5.52 d., J = 3.9 Hz, 1H, 3'-OH), 5.05 (br.sign, 1H, 5'-OH), 4.61 (br.sign., 1H, NH-C<u>H</u>), 4.18 (m, 1H, H-2'), 4.12 (m, 1H, H-3'), 3.79 (m, 1H, H-4'), 3.68 and (m, 1H, H-5'a), 3.66 (m, 1H, H-5'b), 1.42 (d., J = 6.9 Hz, 3H, CH_3) ppm.

13C NMR (176 MHz, DMSO-d6, 30 °C): δ= 140.48 (C8) 83.78 (C4'), 83.41 (C1'), 75.21 (C2'), 74.29 (C3'), 60.30 (C5'), 48.88 (NH-CH), 18.07 (CH_3) ppm.

^{15}N NMR (71 MHz, DMSO-d_6, 30 °C) δ= 239.17 (N7), 169.09 (N9), 105.94 (CO-NH_2), 97.99 (C6-NH) ppm.

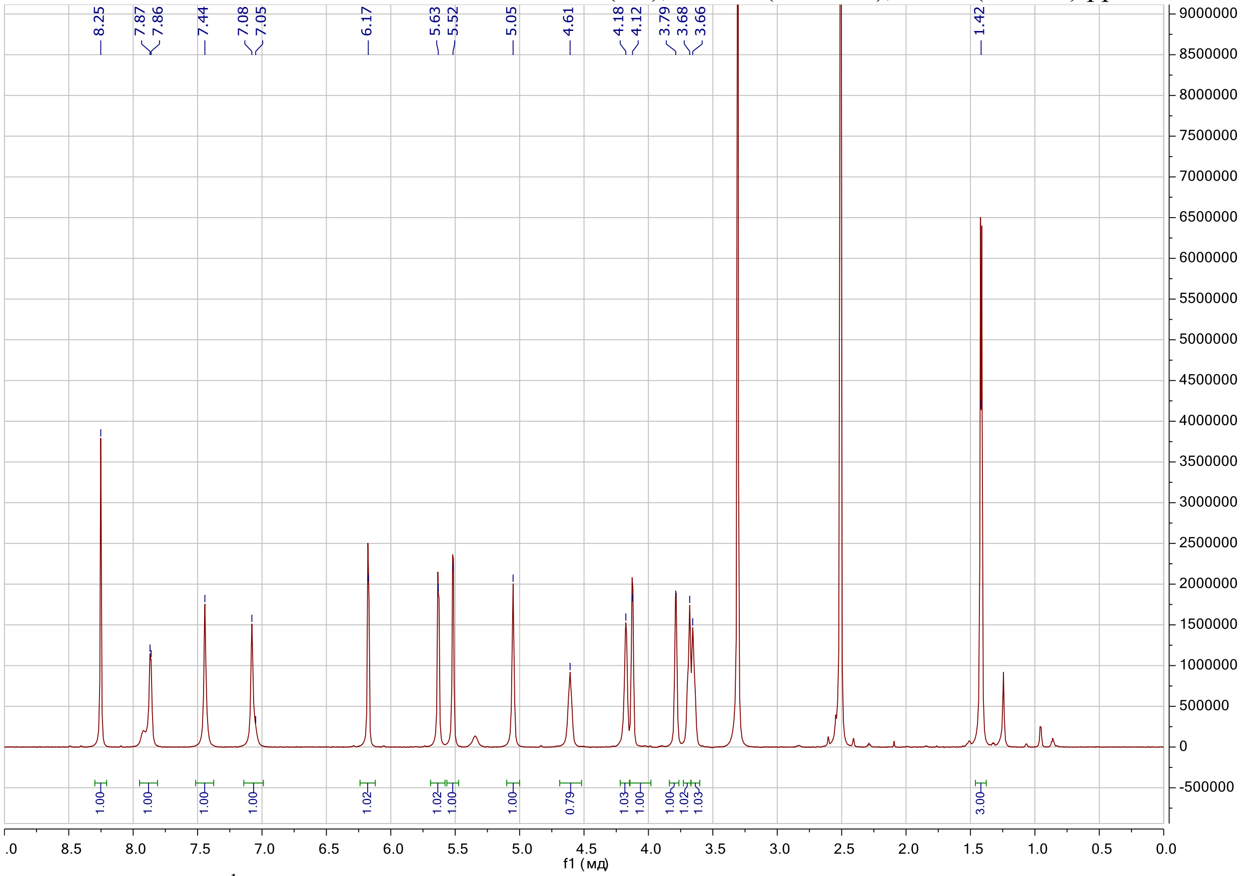

Figure SI-7. The ^{1}H NMR spectrum of 9-*β*-*D*-arabinofuranosyl-2-chloro-6-(N$^{\alpha}$-L-alaninylamido)-purine **(2b)**

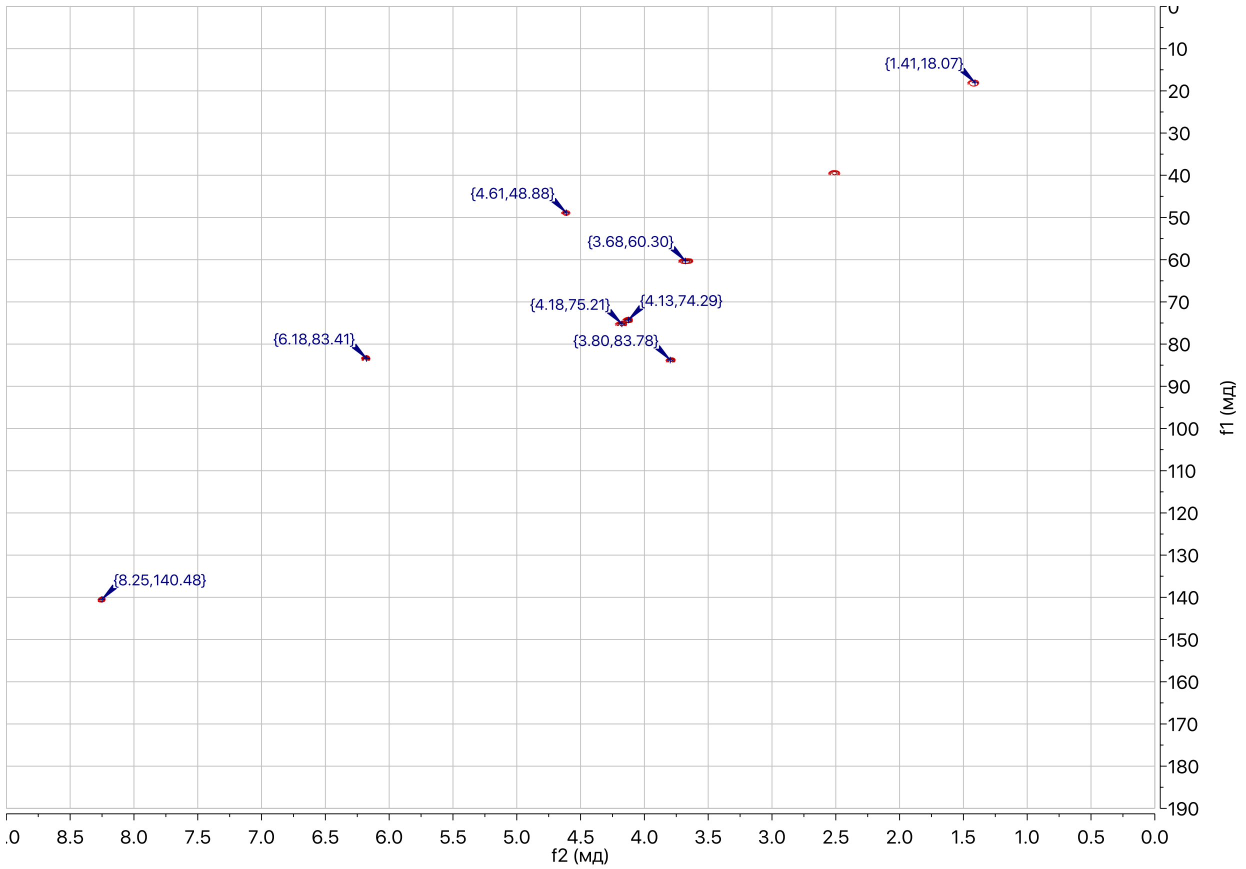

Figure SI-8. The ^{13}C HSQC NMR spectrum of 9-*β*-*D*-arabinofuranosyl-2-chloro-6-(N^{α}-L-alaninylamido)-purine **(2b)**

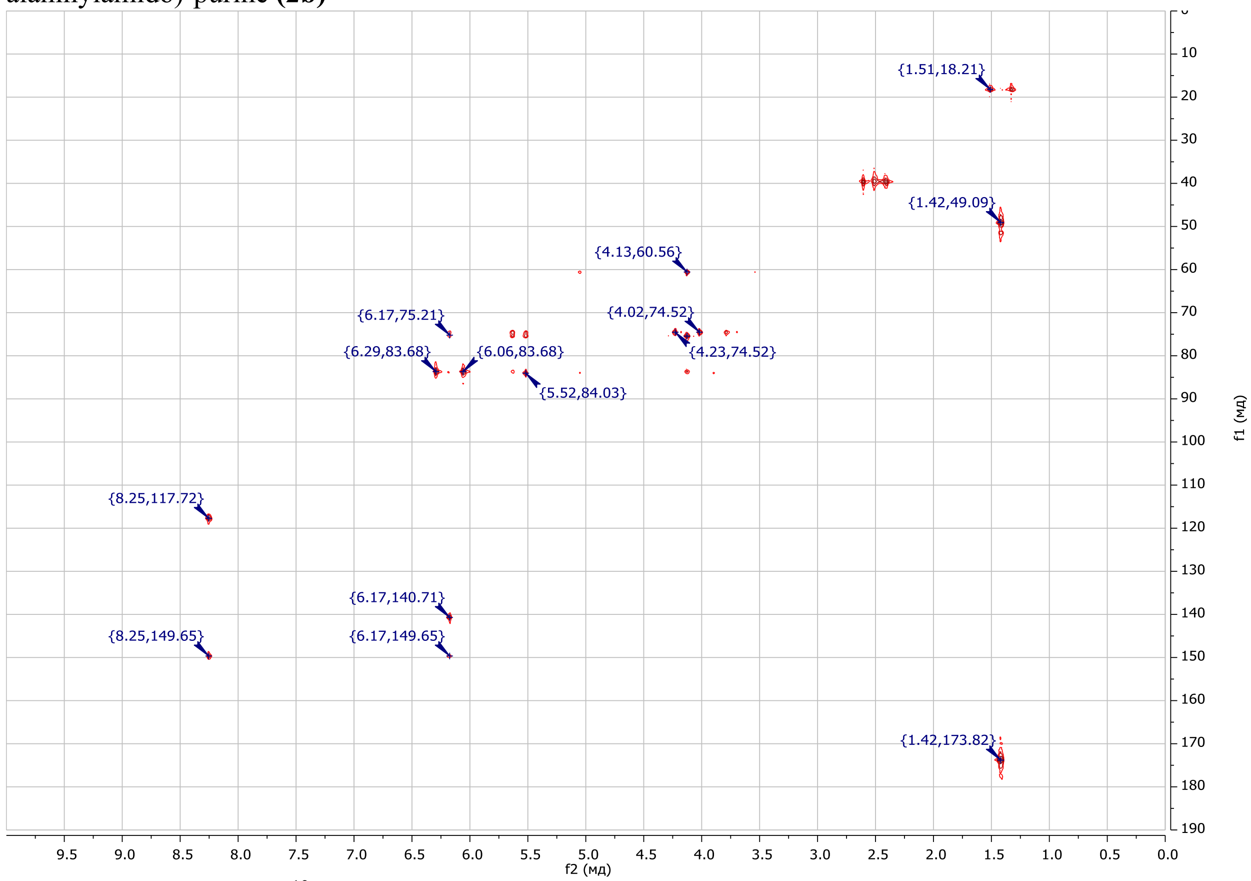

Figure SI-9. The ^{13}C HMBC NMR spectrum of 9-*β*-*D*-arabinofuranosyl-2-chloro-6-(N^{α}-L-alaninylamido)-purine **(2b)**

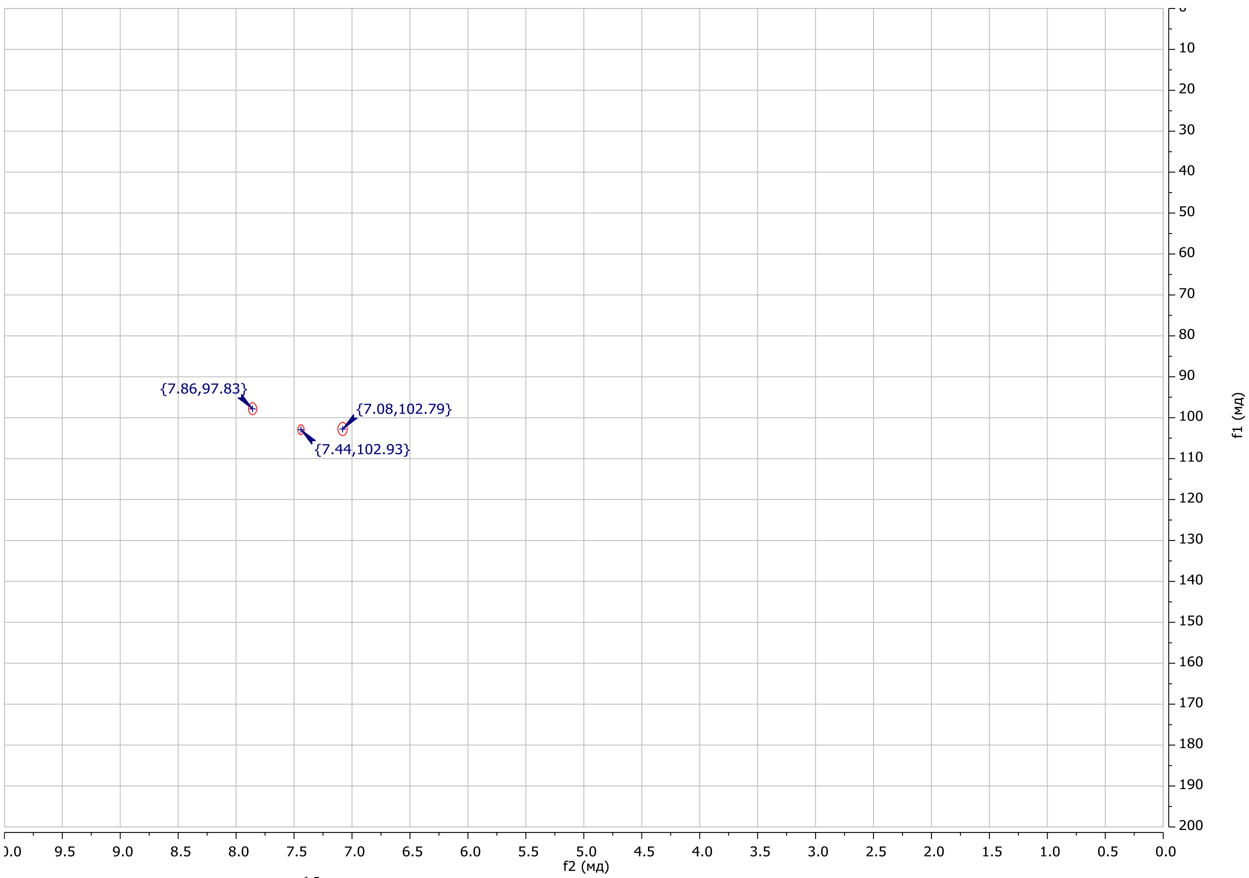

Figure SI-10. The ^{15}N HSQC NMR spectrum of 9-*β*-*D*-arabinofuranosyl-2-chloro-6-(N$^{\alpha}$-L-alaninylamido)-purine **(2b)**

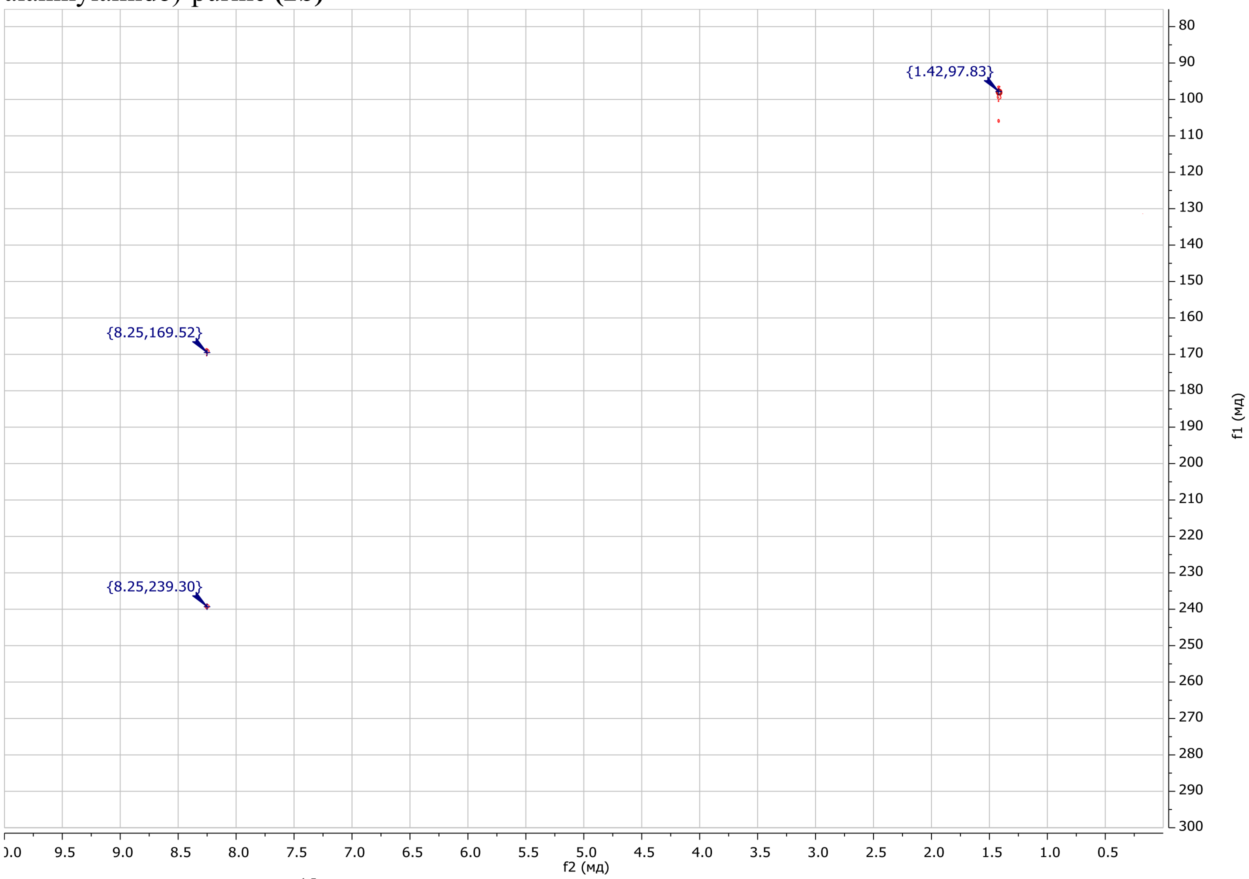

Figure SI-11. The ^{15}N HMBC NMR spectrum of 9-*β*-*D*-arabinofuranosyl-2-chloro-6-(N$^{\alpha}$-L-alaninylamido)-purine **(2b)**

IBCH

Project Name: TESTLast

Reported by User: System

Breeze

SAMPLE INFORMATION

Sample Name:	**2b**	Acquired By:	System
Sample Type:	Unknown	Date Acquired:	13.05.2013 17:42:38
Vial:	1	Acq. Method:	100B_dual_280
Injection #:	1	Date Processed:	13.05.2013 19:03:34
Injection Volume:	15,00 ul	Channel Name:	2487Channel 2
Run Time:	20,00 Minutes	Sample Set Name:	
Column Type:			

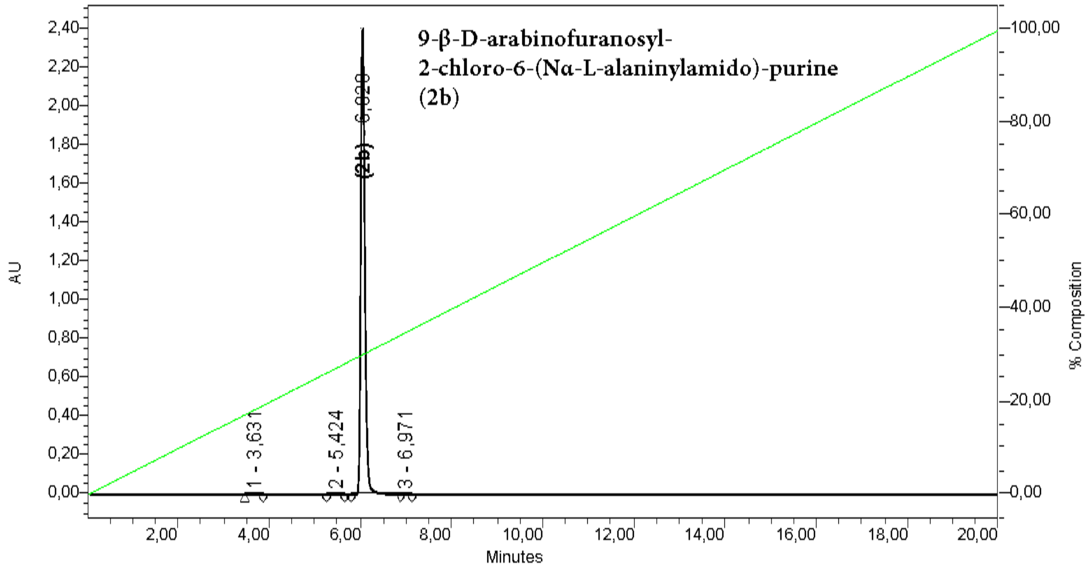

	Peak Name	RT (min)	Area (V*sec)	% Area	Height (V)	% Height
1	1	3,631	15291	0,10	1632	0,07
2	2	5,424	17430	0,12	1846	0,08
3	**2b**	6,028	15112678	99,72	2416720	99,81
4	3	6,971	10050	0,07	1043	0,04

Report Method: Gradient Overlay Report ASC   Printed 19:04:17   13.05.2013   Page: 1 of 1

Figure SI-12. The chromatogram of **2b**

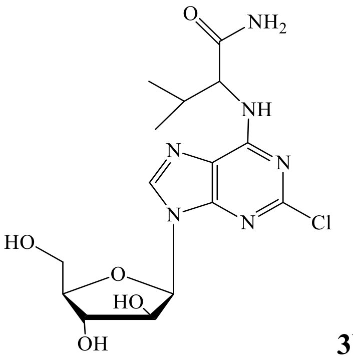

3b

9-*β*-*D*-arabinofuranosyl-2-chloro-6-(N^{α}-L-valinylamido)-purine (3b)

^{1}H NMR (700 MHz, DMSO-d_6, 30 °C): δ= 8.27 (s, 1H, H-8), 7.77 and 7.43 (2 sign, 0.2H and *J* = 7.65Hz, 0.78H, C6-NH), CO-NH, 7.54 and 7.14 (2 sign, 0.8H and 0.23H, CO-NH), 7.37 and 7.18 (2 sign, 0.22H and 0.77H, CO-NH), 6.18 (d, J =7.65 Hz, 1H, H-1'), 5.63 (br.sign, 1H, 2'-OH), 5.54 (br.sign, 1H, 3'-OH), 5.32 and 5.06 (2 sign, 0.2H and 0.8H, 5'-OH), 4.63 (m, 0.8H, CβH) 4.18 (m, 1H, H-2'), 4.13 (m, 1H, H-3') 3.79 (m, 1H, H-4') 3.69 (m, 1H, H-5'a), 3.65 (m, 1H, H-5'b), 2.18 (m, 1H, CαH) 0.96 (m, 3H, CH_3) 0.94 (m, 3H, CH_3) ppm.

^{13}C NMR (176 MHz, DMSO-d_6, 30 °C): δ= 172.40 (CO-NH_2), 154.57 (C6), 152.62 (C2), 149.69 (C4), 140.90 (C8), 117.68 (C5), 84.06 (C4'), 83.74 (C1'), 75.47 (C2'), 74.52, (C3'), 60.57 (C5'), 58.86 (Cα), 30.54 (Cβ), 19.14 (CH_3), 18.29 (CH_3) ppm.

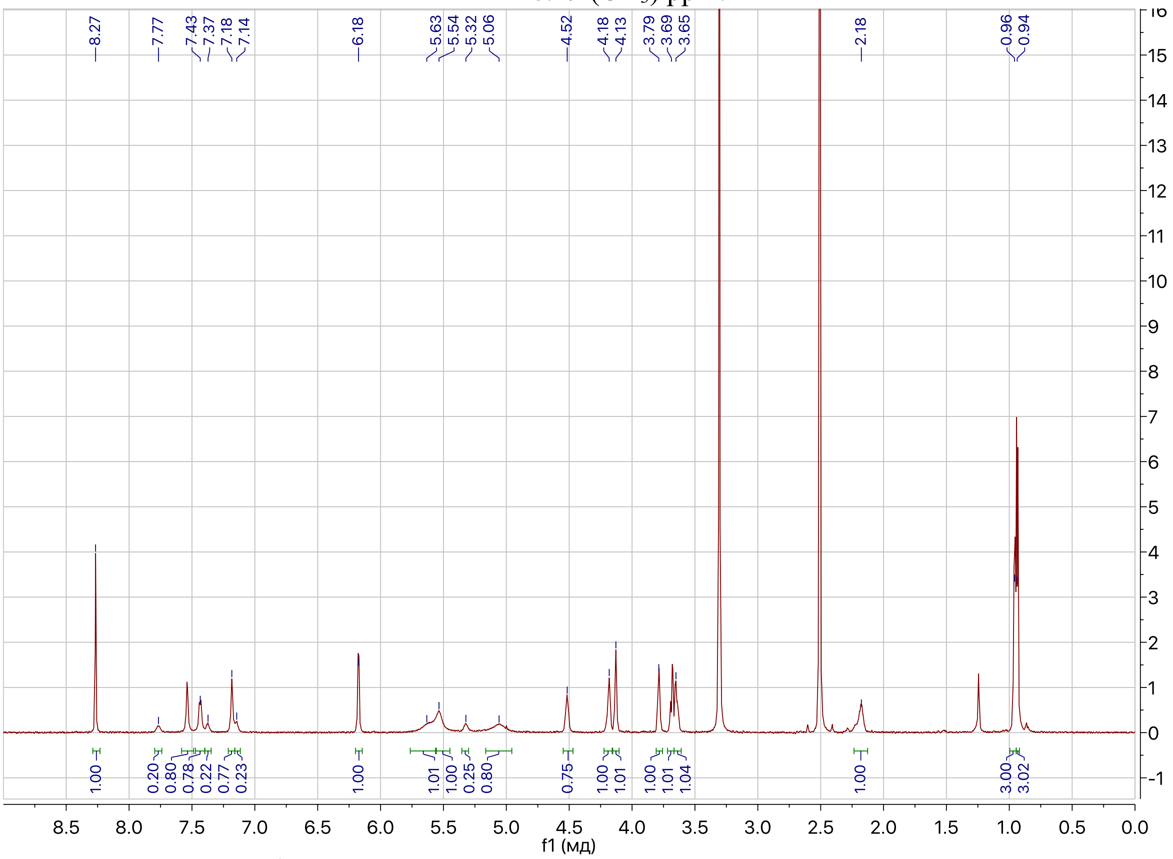

Figure SI-13. The ^{1}H NMR spectrum of 9-*β*-*D*-arabinofuranosyl-2-chloro-6-(N^{α}-L-valinylamido)-purine **(3b)**

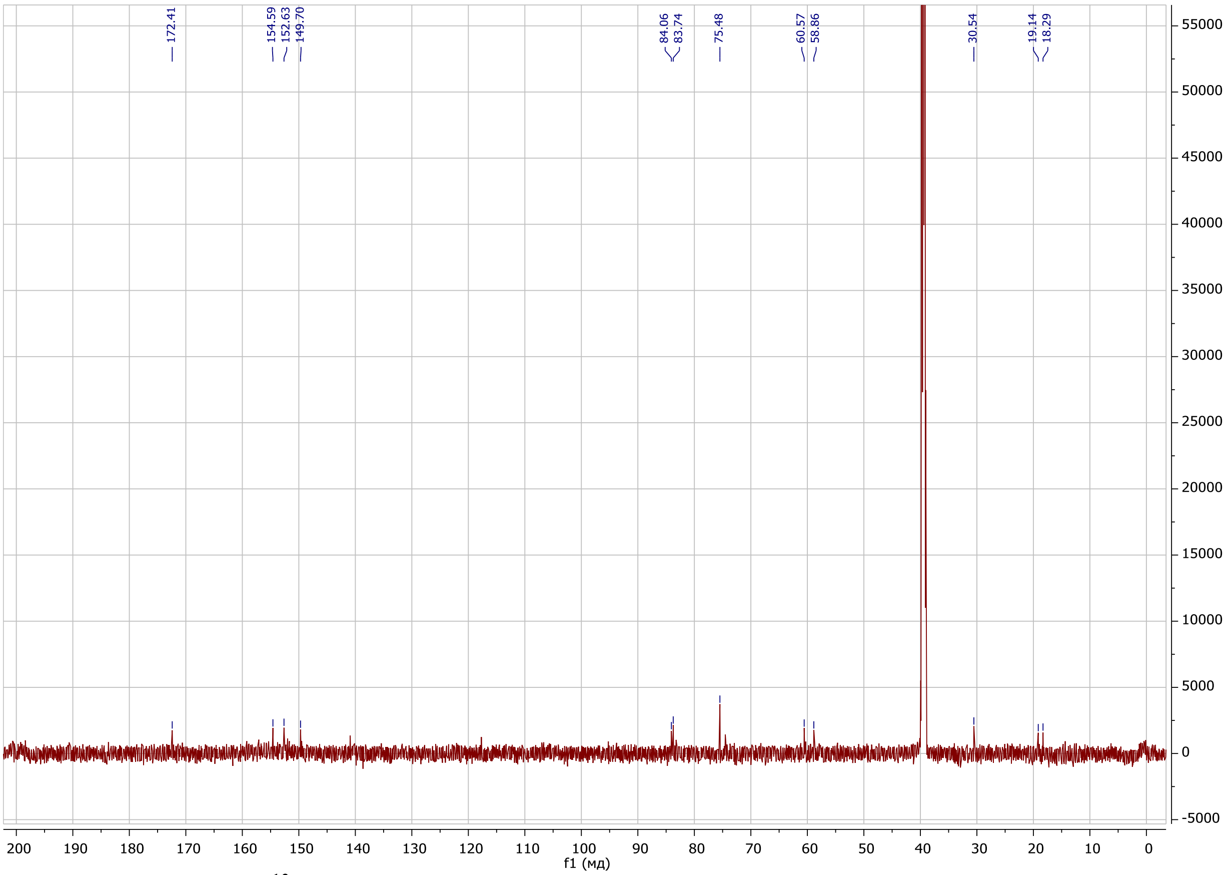

Figure SI-14. The ^{13}C NMR spectrum of 9-*β*-*D*-arabinofuranosyl-2-chloro-6-(N$^{\alpha}$-L-valinylamido)-purine **(3b)**

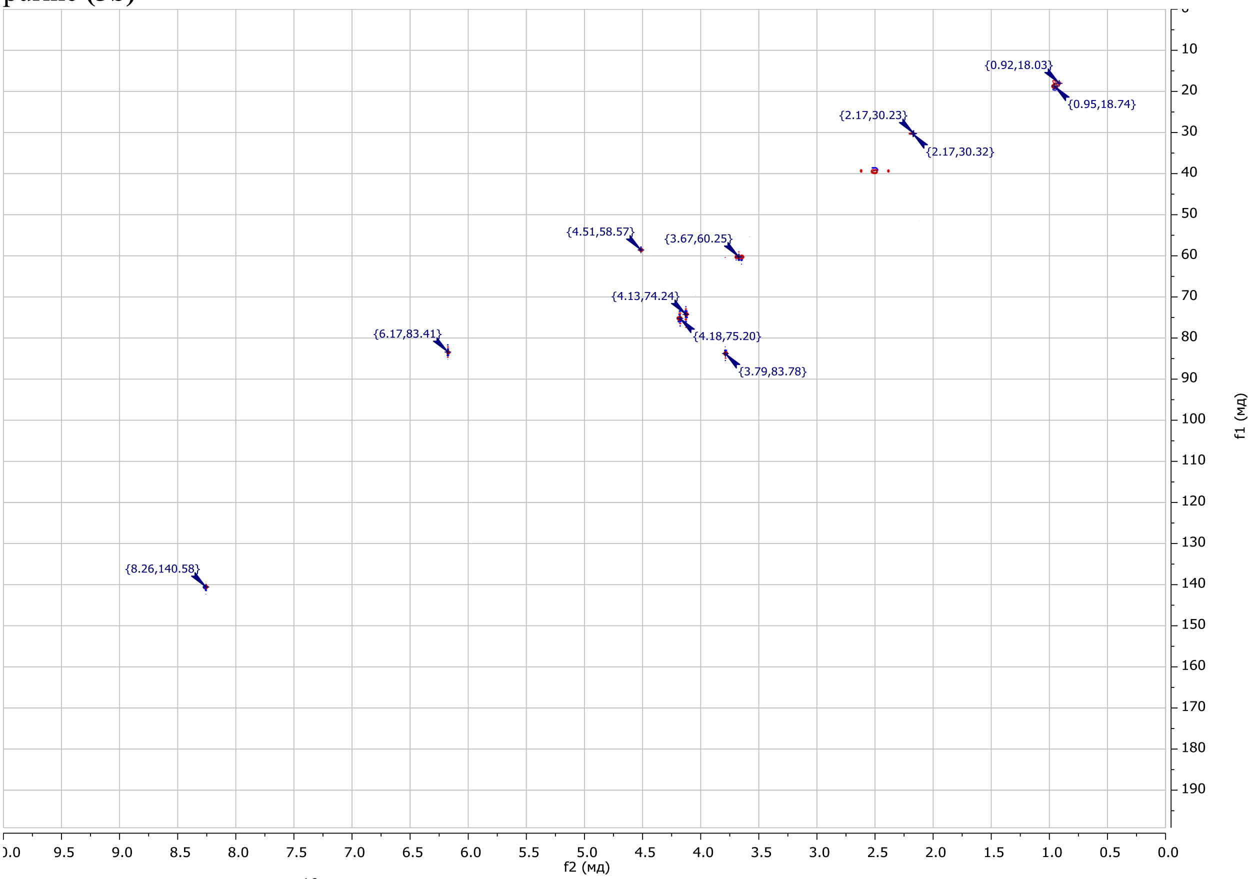

Figure SI-15. The ^{13}C HSQC NMR spectrum of 9-*β*-*D*-arabinofuranosyl-2-chloro-6-(N$^{\alpha}$-L-valinylamido)-purine **(3b)**

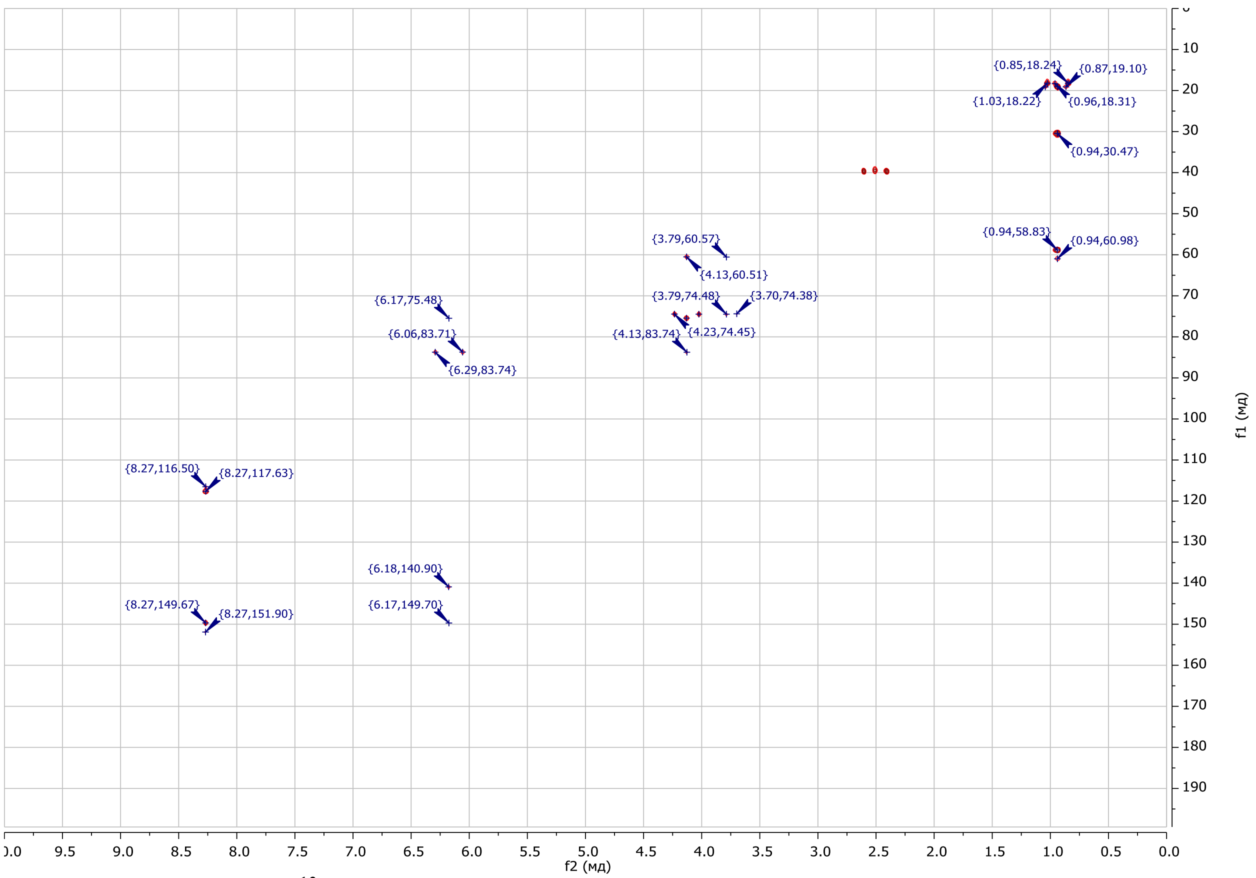

Figure SI-16. The ^{13}C HMBC NMR spectrum of 9-*β*-*D*-arabinofuranosyl-2-chloro-6-(N^{α}-L-valinylamido)-purine **(3b)**

BT
Project Name: TESTLast_1
Reported by User: System

SAMPLE INFORMATION

Sample Name:	**3b**	Acquired By:	System
Sample Type:	Unknown	Date Acquired:	29.04.2014 13:56:04
Vial:	1	Acq. Method:	100B_dual_280
Injection #:	1	Date Processed:	19.05.2014 12:47:21
Injection Volume:	7,50 ul	Channel Name:	2487Channel 2
Run Time:	20,00 Minutes	Sample Set Name:	
Column Type:			

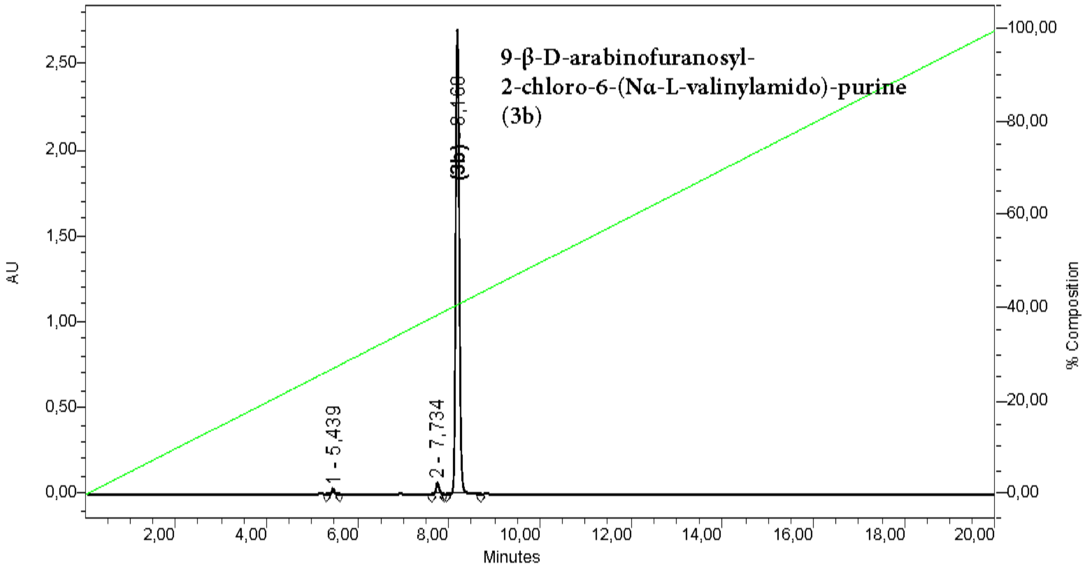

	Peak Name	RT (min)	Area (µV*sec)	% Area	Height (µV)	% Height
1	1	5,439	142119	0,92	32728	1,16
2	2	7,734	331536	2,14	65046	2,30
3	**3b**	8,168	15052791	96,95	2725743	96,54

Report Method: Gradient Overlay Report ASC Printed 12:47:41 19.05.2014 Page: 1 of 1

Figure SI-17. The chromatogram of **3b**

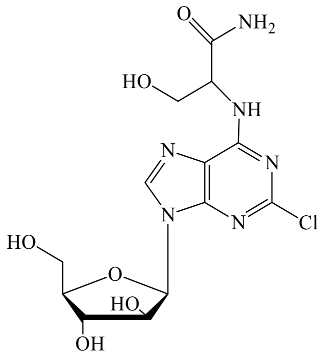

4b

9-*β*-*D*-arabinofuranosyl-2-chloro-6-(N$^{\alpha}$-L-serinylamido)-purine (4b)

^{1}H NMR (700 MHz, DMSO-d_6, 30 °C): δ= 8.27 and 8.25 (2 sign., 0.84H and 0.14, H-8), 7.60 and 7.55 (br.sign, 0.18H, and br.d., *J* = 6.2 Hz, 0.82H, C6-NH), 7.47 and 7.45 (2 sign., 0.78H and 0.24H, CO-NH), 7.16 (s, 1H, CO-NH), 6.18 (d, *J* = 5.0 Hz, 1H, H-1'), 5.64 (br.sign, 1H, 2'-OH), 5.52 (br.d., *J* = 3.4 Hz, 1H, 3'-OH), 5.05 (br.sign, 1H, 5'-OH), 5.04 and 4.93 (2 sign, 0.80H and 0.2H, OH-Ser), 4.63 (m, 0.80H, CαH) 4.18 (m, 1H, H-2'), 4.13 (m, 1H, H-3') 3.81 (m, 2H, C<u>H</u>$_2$OH) 3.79 (m, 1H, H-4') 3.68 (m, 1H, H-5'a), 3.66 (m, 1H, H-5'b) ppm.

^{13}C NMR (176 MHz, DMSO-d_6, 30 °C): δ= 140.57 (C8), 83.79 (C4'), 83.43 (C1'), 75.21 (C2'), 74.30 (C3'), 61.30 (CH_2OH), 60.33 (C5'), 55.74 (Cα) ppm.

^{15}N NMR (71 MHz, DMSO-d_6, 30 °C) δ= 238.71 (N7), 169.31 (N9), 104.81 (CO-NH_2), 90.85 (C6-NH) ppm.

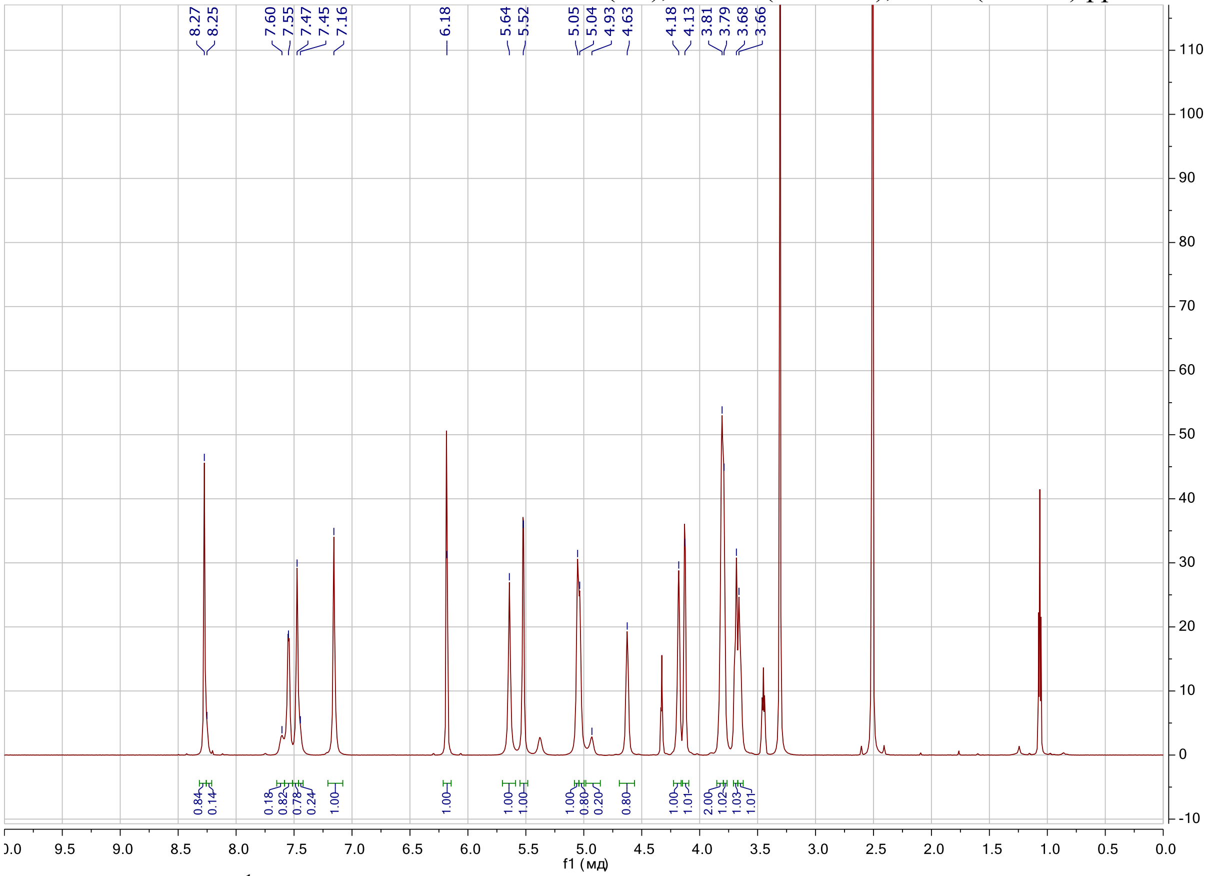

Figure SI-18. The ^{1}H NMR spectrum of 9-*β*-*D*-arabinofuranosyl-2-chloro-6-(N$^{\alpha}$-L-serinylamido)-purine **(4b)**

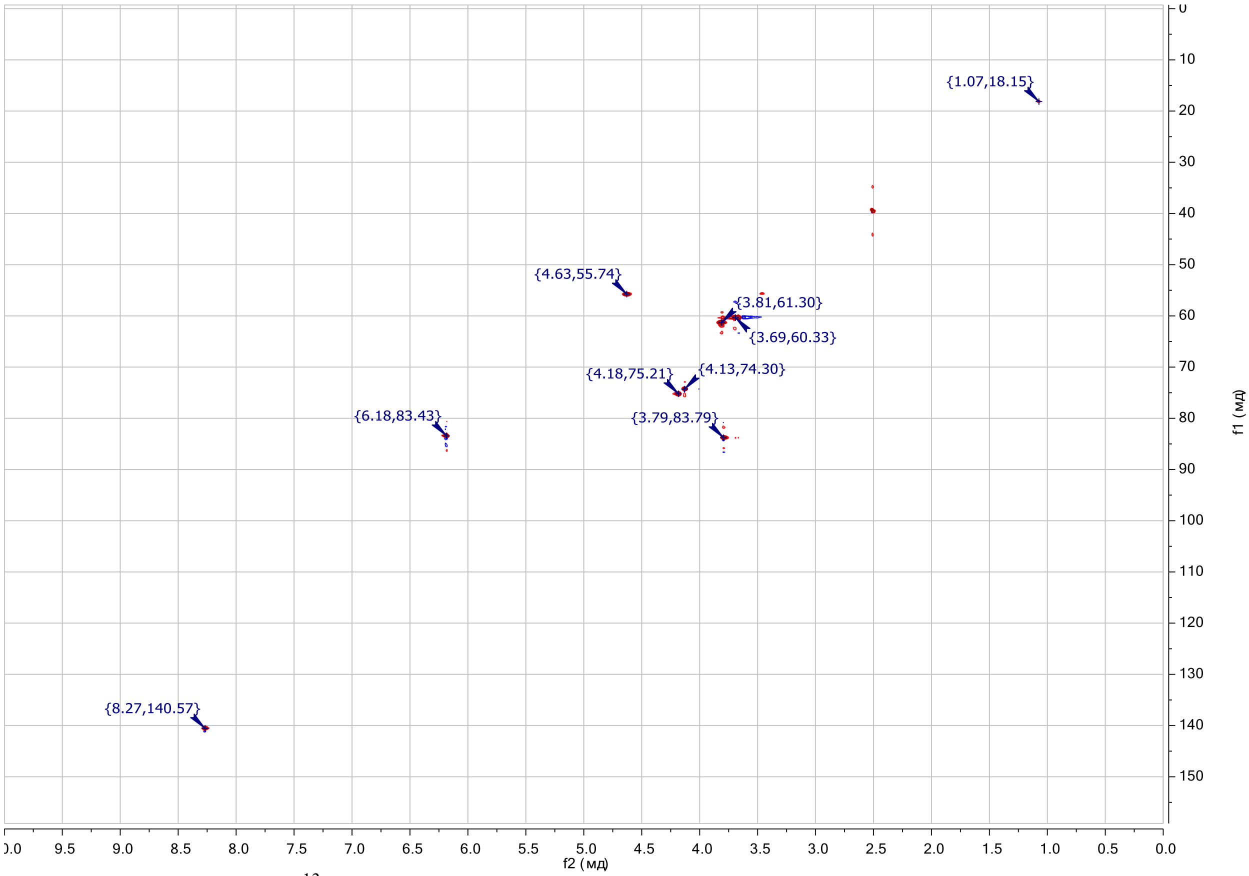

Figure SI-19. The ^{13}C HSQC NMR spectrum of 9-*β*-*D*-arabinofuranosyl-2-chloro-6-(N$^{\alpha}$-L-serinylamido)-purine **(4b)**

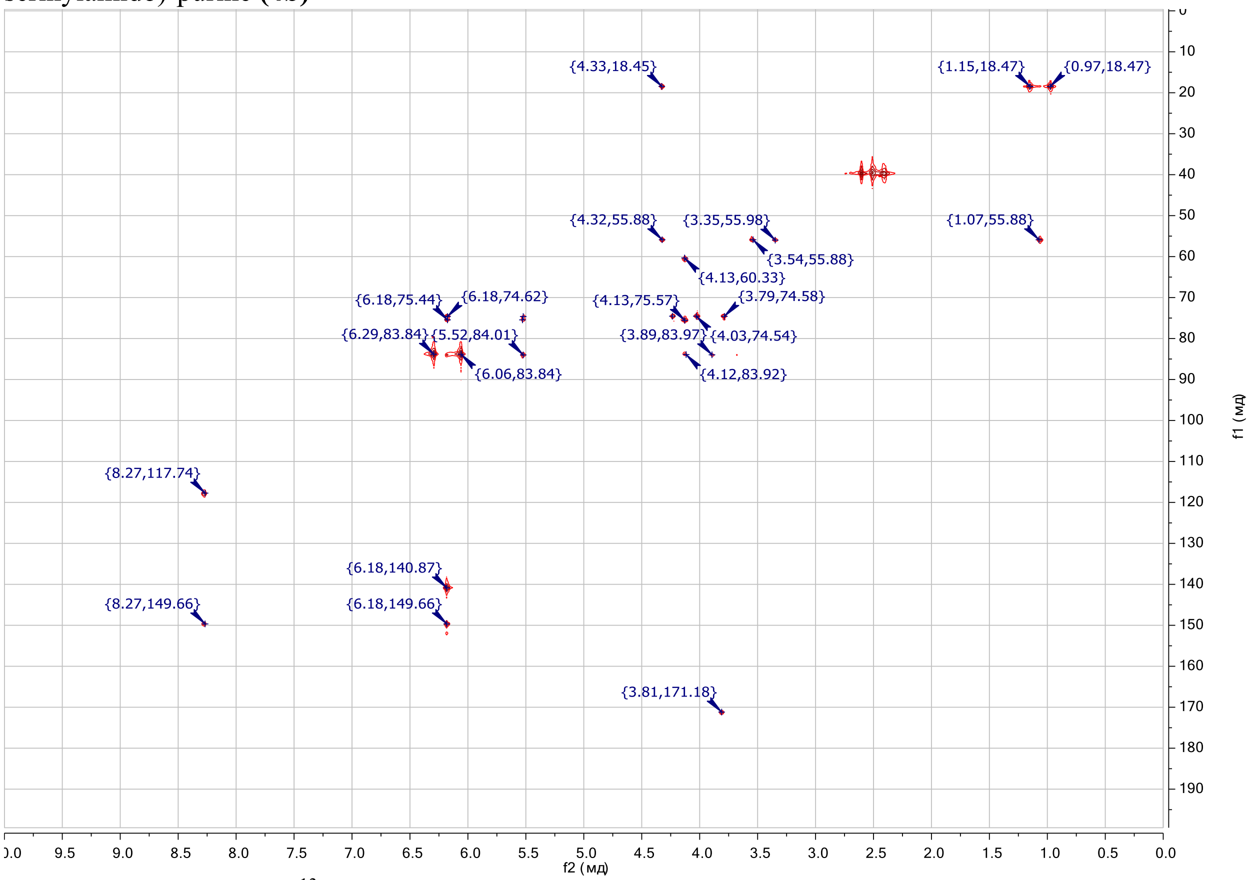

Figure SI-20. The ^{13}C HMBC NMR spectrum of 9-*β*-*D*-arabinofuranosyl-2-chloro-6-(N$^{\alpha}$-L-serinylamido)-purine **(4b)**

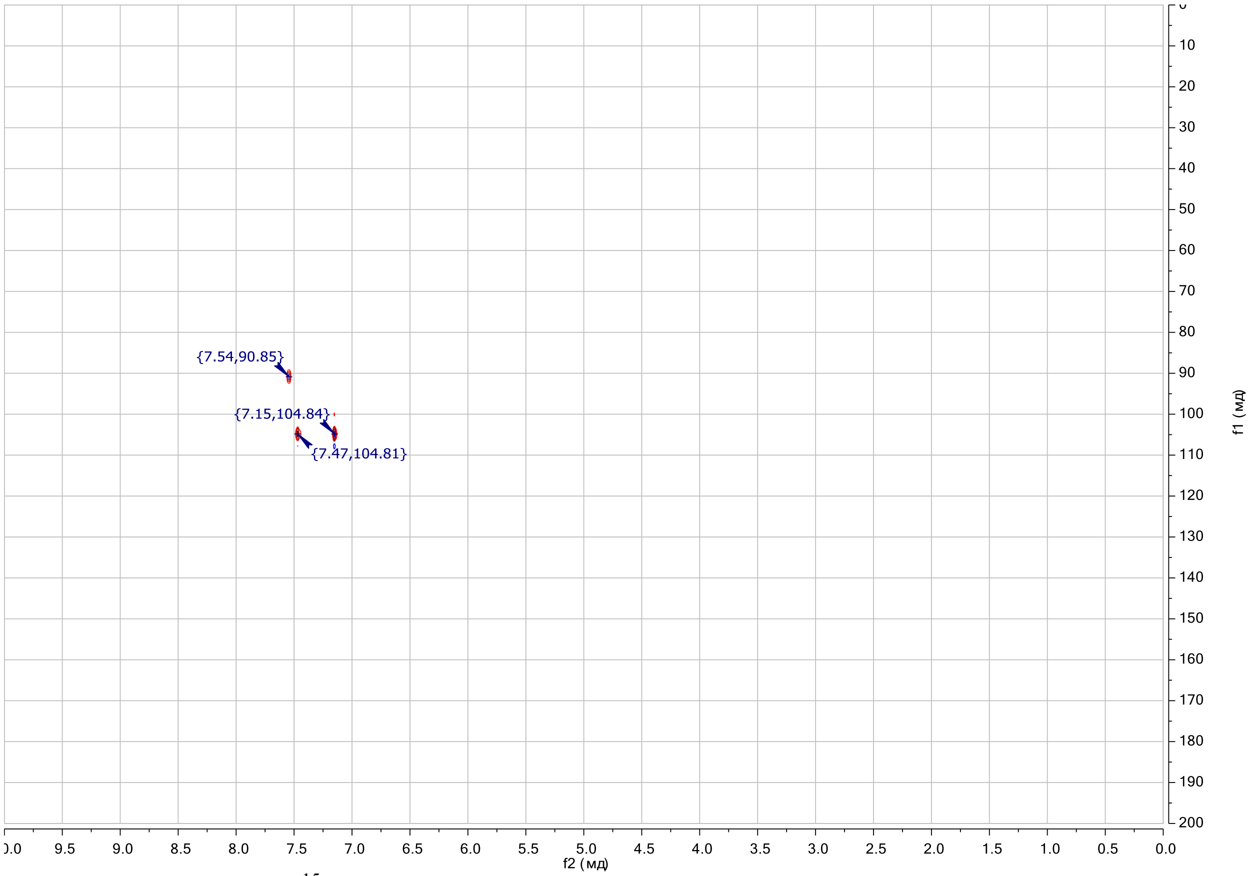

Figure SI-21. The ^{15}N HSQC NMR spectrum of 9-*β*-*D*-arabinofuranosyl-2-chloro-6-(N$^{\alpha}$-L-serinylamido)-purine **(4b)**

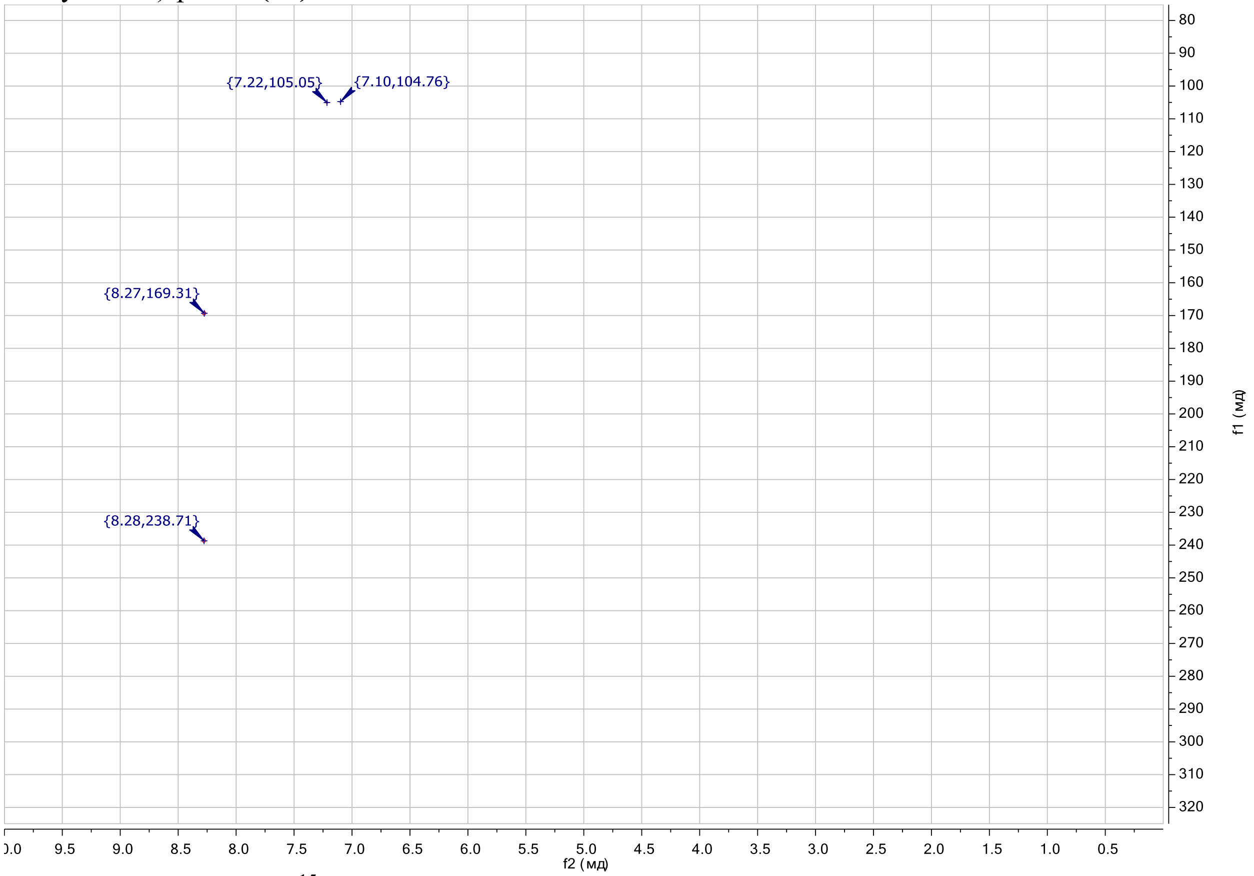

Figure SI-22. The ^{15}N HMBC NMR spectrum of 9-*β*-*D*-arabinofuranosyl-2-chloro-6-(N$^{\alpha}$-L-serinylamido)-purine **(4b)**

IBCH
Project Name: TESTLast
Reported by User: System

SAMPLE INFORMATION

Sample Name:	**4b**	Acquired By:	System
Sample Type:	Unknown	Date Acquired:	13.05.2013 18:18:45
Vial:	1	Acq. Method:	100B_dual_280
Injection #:	2	Date Processed:	13.05.2013 19:08:08
Injection Volume:	15,00 ul	Channel Name:	2487Channel 2
Run Time:	20,00 Minutes	Sample Set Name:	
Column Type:			

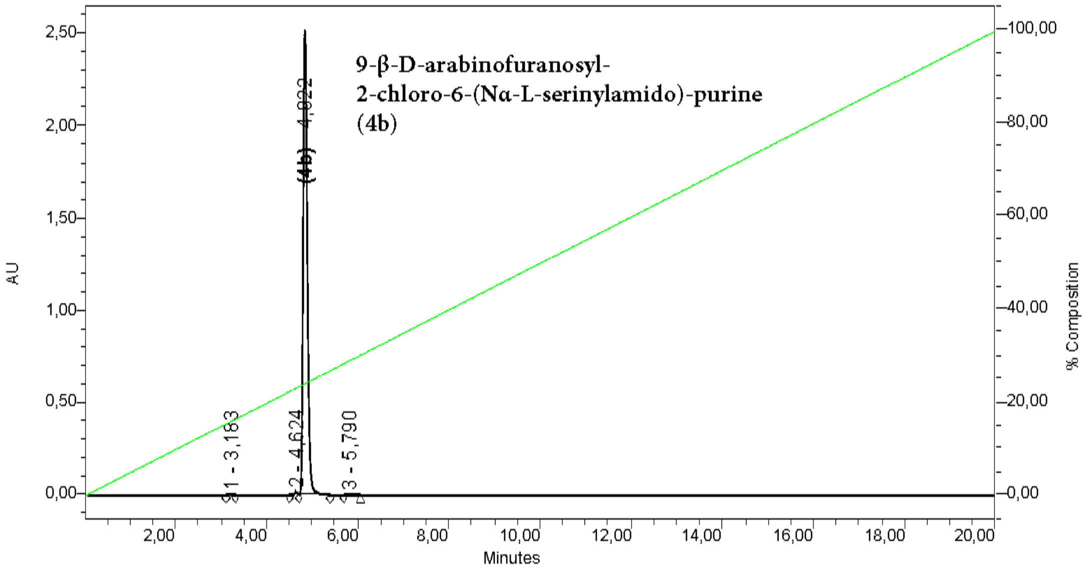

	Peak Name	RT (min)	Area (µV*sec)	% Area	Height (µV)	% Height
1	1	3,183	15970	0,10	3406	0,13
2	2	4,624	88626	0,54	18371	0,71
3	**4b**	4,822	16167205	99,27	2560363	99,09
4	3	5,790	14099	0,09	1856	0,07

Report Method: Gradient Overlay Report ASC Printed 19:08:24 13.05.2013 Page: 1 of 1

Figure SI-23. The chromatogram of **4b**

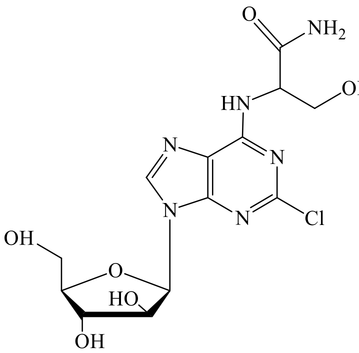

5b

9-*β*-*D*-arabinofuranosyl-2-chloro-6-(N$^{\alpha}$-D-serinylamido)-purine (5b)

^{1}H NMR (700 MHz, DMSO-d_6, 30 °C): δ= 8.28 and 8.25 (2 sign., 0.85H and 0.25H, H-8), 7.60 and 7.53 (2 sign., 0.05H and 0.83H, C6-NH), 7.49 and 7.14 (2br.sign, 0.88H, 0.22H, CO-NH), 7.45 and 7.18 (2 sign., 0.16 and 0.91H, CO-NH), 6.18 (d, *J* = 5.2 Hz, 1H, H-1'), 5.64 (br.sign, 1H, 2'-OH), 5.54 (br.sign, 1H, 3'-OH), 5.06 (br.sign, 1H, 5'-OH), 5.02 (br.sign, 1H, OH-Ser), 4.63 (m, 1H, CαH) 4.19 (m, 1H, H-2'), 4.13 (m, 1H, H-3') 3.80 (m, 2H, C<u>H</u>$_2$OH) 3.79 (m, 1H, H-4') 3.68 (m, 1H, H-5'a), 3.66 (m, 1H, H-5'b) ppm.

^{13}C NMR (176 MHz, DMSO-d_6, 30 °C): δ= 171.29 (CO-NH$_2$), 154.36 (C6), 152.59 (C2) , 149.65 (C4), 140.85 (C8), 117.87 (C5), 84.00 (C4'), 83.68 (C1'), 75.48 (C2'), 74.46 (C3'), 61.56 (CH$_2$OH), 60.54 (C5'), 55.99 (Cα) ppm.

^{15}N NMR (71 MHz, DMSO-d_6, 30 °C) δ= 238.70 (N7), 169.71(N9), 105.13 (CO-NH$_2$), 90.85 (C6-NH) ppm.

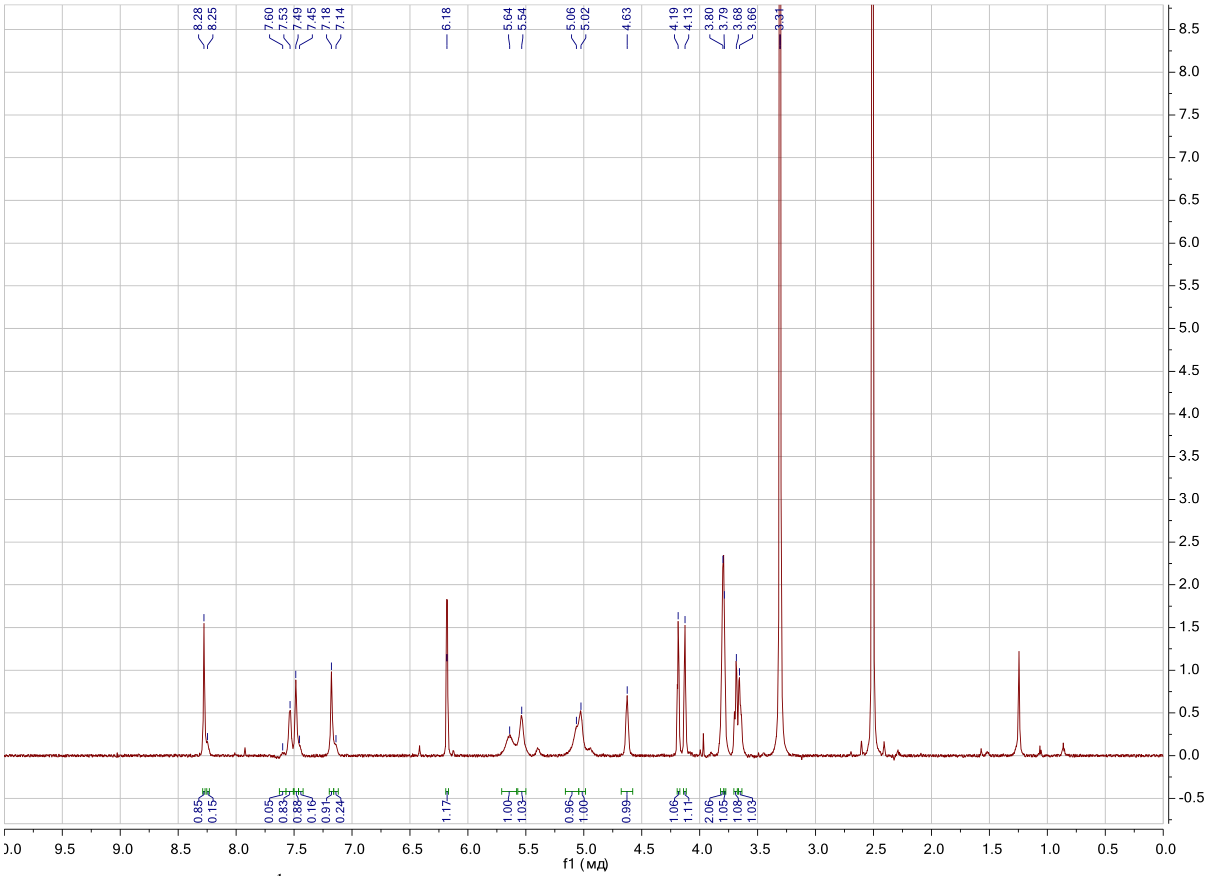

Figure SI-24. The ^{1}H NMR spectrum of 9-*β*-*D*-arabinofuranosyl-2-chloro-6-(N$^{\alpha}$-D-serinylamido)-purine **(5b)**

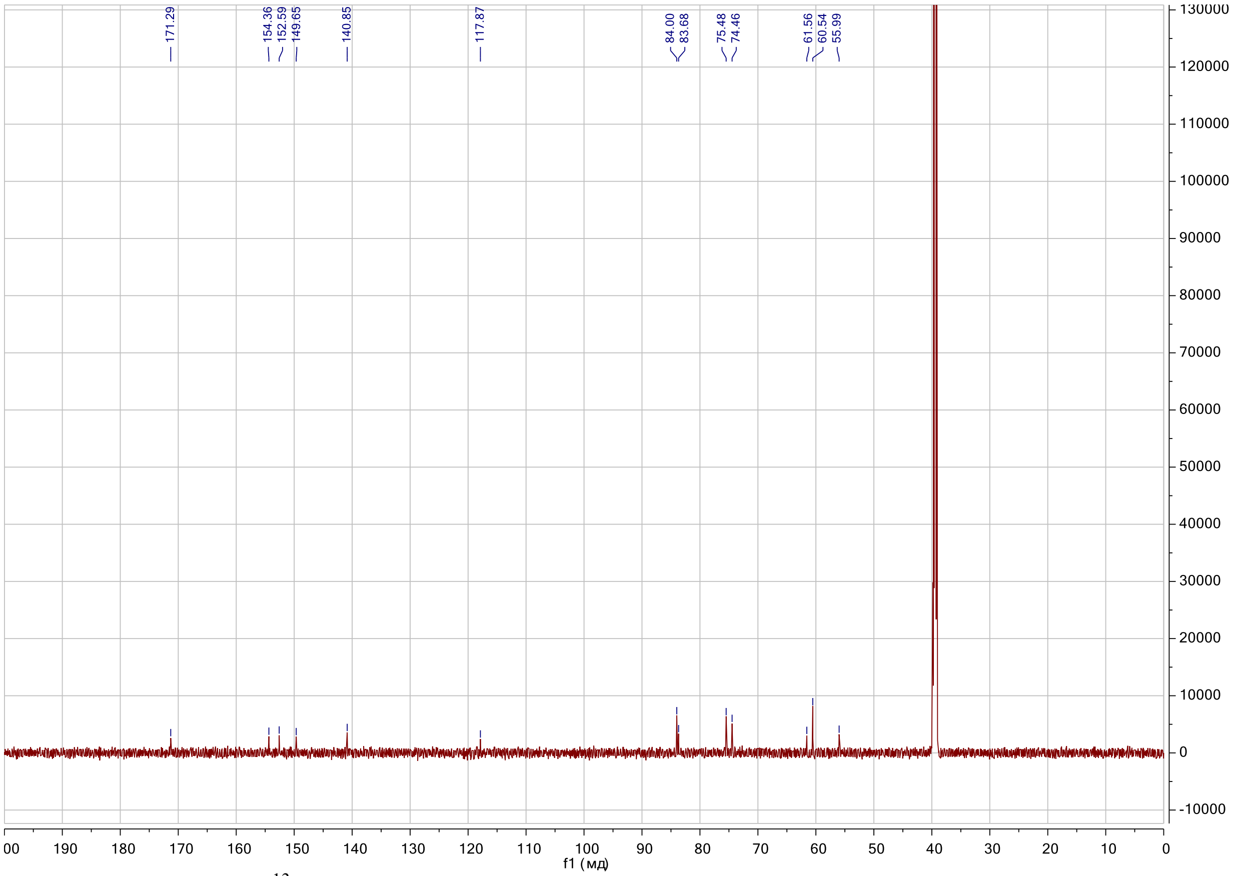

Figure SI-25. The ^{13}C NMR spectrum of 9-*β*-*D*-arabinofuranosyl-2-chloro-6-(N^{α}-D-serinylamido)-purine **(5b)**

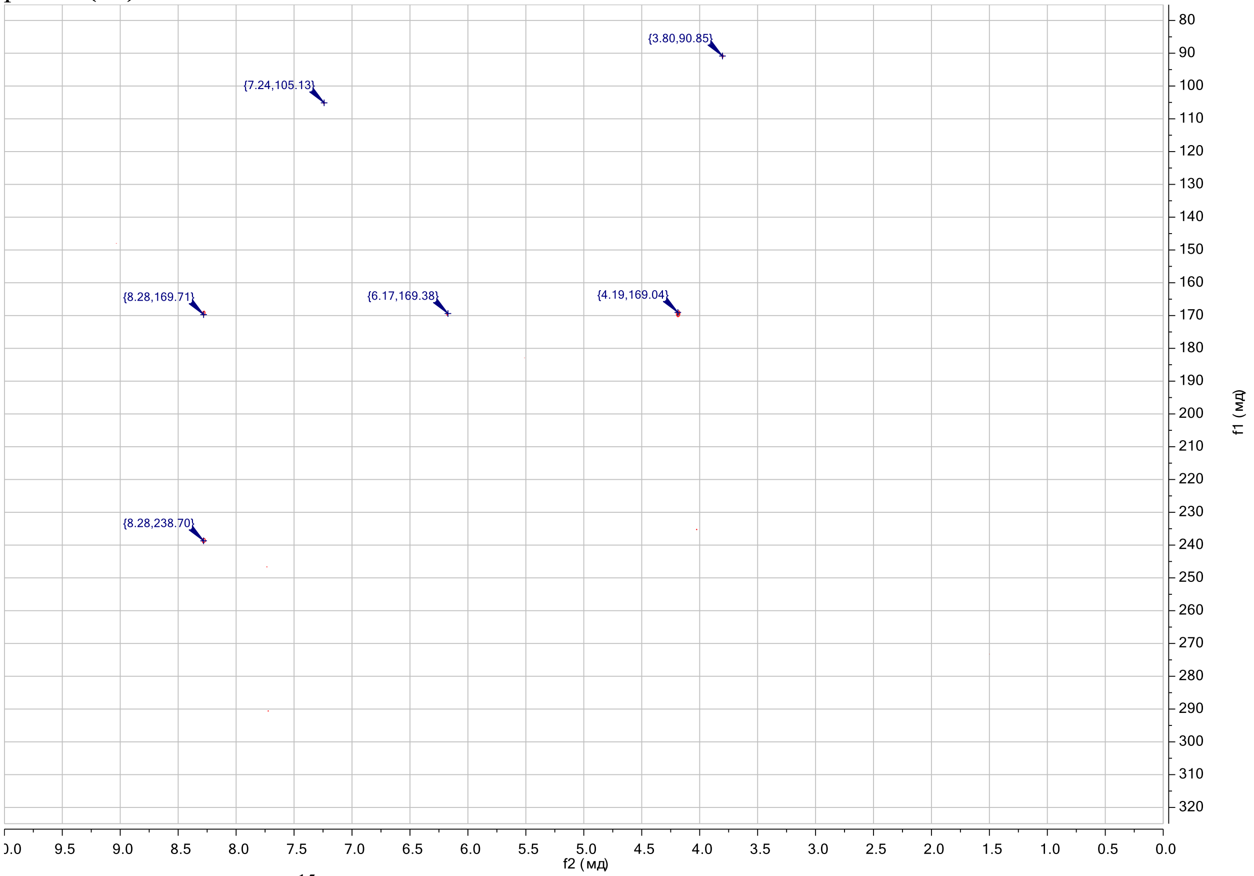

Figure SI-26. The ^{15}N HMBC NMR spectrum of 9-*β*-*D*-arabinofuranosyl-2-chloro-6-(N^{α}-D-serinylamido)-purine **(5b)**

BT

Project Name: TESTLast_1
Reported by User: System

SAMPLE INFORMATION

Sample Name:	**5b**	Acquired By:	System
Sample Type:	Unknown	Date Acquired:	29.04.2014 14:51:15
Vial:	1	Acq. Method:	100B_dual_280
Injection #:	1	Date Processed:	19.05.2014 12:46:34
Injection Volume:	7,50 ul	Channel Name:	2487Channel 2
Run Time:	20,00 Minutes	Sample Set Name:	
Column Type:			

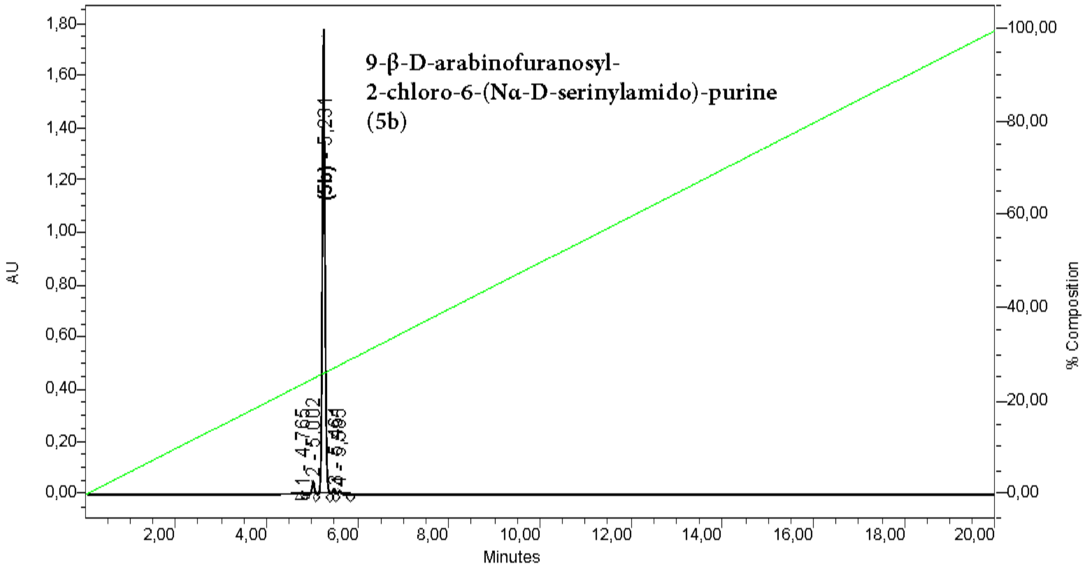

	Peak Name	RT (min)	Area (µV*sec)	% Area	Height (µV)	% Height
1	1	4,765	19107	0,25	5603	0,31
2	2	5,002	178936	2,37	44540	2,48
3	**5b**	5,231	7233525	95,69	1717182	95,58
4	3	5,461	75283	1,00	17226	0,96
5	4	5,585	52282	0,69	11999	0,67

Report Method: Gradient Overlay Report ASC Printed 12:46:41 19.05.2014 Page: 1 of 1

Figure SI-27. The chromatogram of **5b**

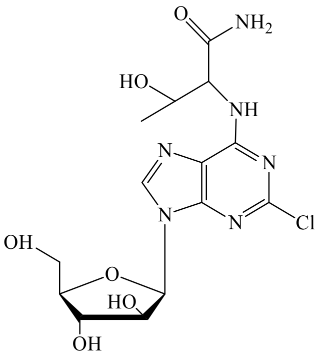

6b

9-*β*-*D*-arabinofuranosyl-2-chloro-6-(N$^{\alpha}$-L-threoninylamido)-purine (6b)

^{1}H NMR (700 MHz, DMSO-d_6, 30 °C): δ= 8.28 and 8.26 (2 s, 0.78H and 0.22H, H-8), 7.49 and 7.43, (2 sign., 0.97H, CO-NH), 7.20 (d, J = 8.5 Hz, 0.78H, C6-NH) 7.13 and 7.11 (2 sign., 0.98H, CO-NH), 6.18 (d, J = 5.2Hz, 1H, H-1'), 5.64 (d., J = 5.6 Hz, 1H, 2'-OH), 5.52 (d., J = 5.0 Hz, 1H, 3'-OH), 5.11 (br.d, J = 6.0 Hz, 0.76H, OH), 5.05 (t, J = 5.48 Hz, 1H, 5'-OH), 4.53 (br.sign., 0.78H, NH-C<u>H</u>), 4.19 (m, 2H, H-2'), 4.13 (m, 1H, H-3'), 3.79 (m, 1H, H-4'), 3.68 (m, 1H, H-5'a), 3.66 (m, 1H, H-5'b), 1.13 (d, J = 6.6 Hz, 3H, CH_3) ppm.

^{13}C NMR (176 MHz, DMSO-d_6, 30 °C) δ = 171.58 (CO-NH_2), 154.81 (C6), 152.62 (C2), 149.64 (C4), 140.98 (C8), 117.84 (C5), 84.06 (C4'), 83.73 (C1'), 75.47 (C3'), 74.53 (C2'), 66.65 (CH-OH), 60.57 (C5'), 59.12 (NH-CH), 20.40 (CH_3) ppm.

^{15}N NMR (71 MHz, DMSO-d_6, 30 °C) δ= 238.49 (N7), 227.23 (N1), 169.27 (N9), 105.15 (CO-NH_2), 87.62 (C6-NH) ppm.

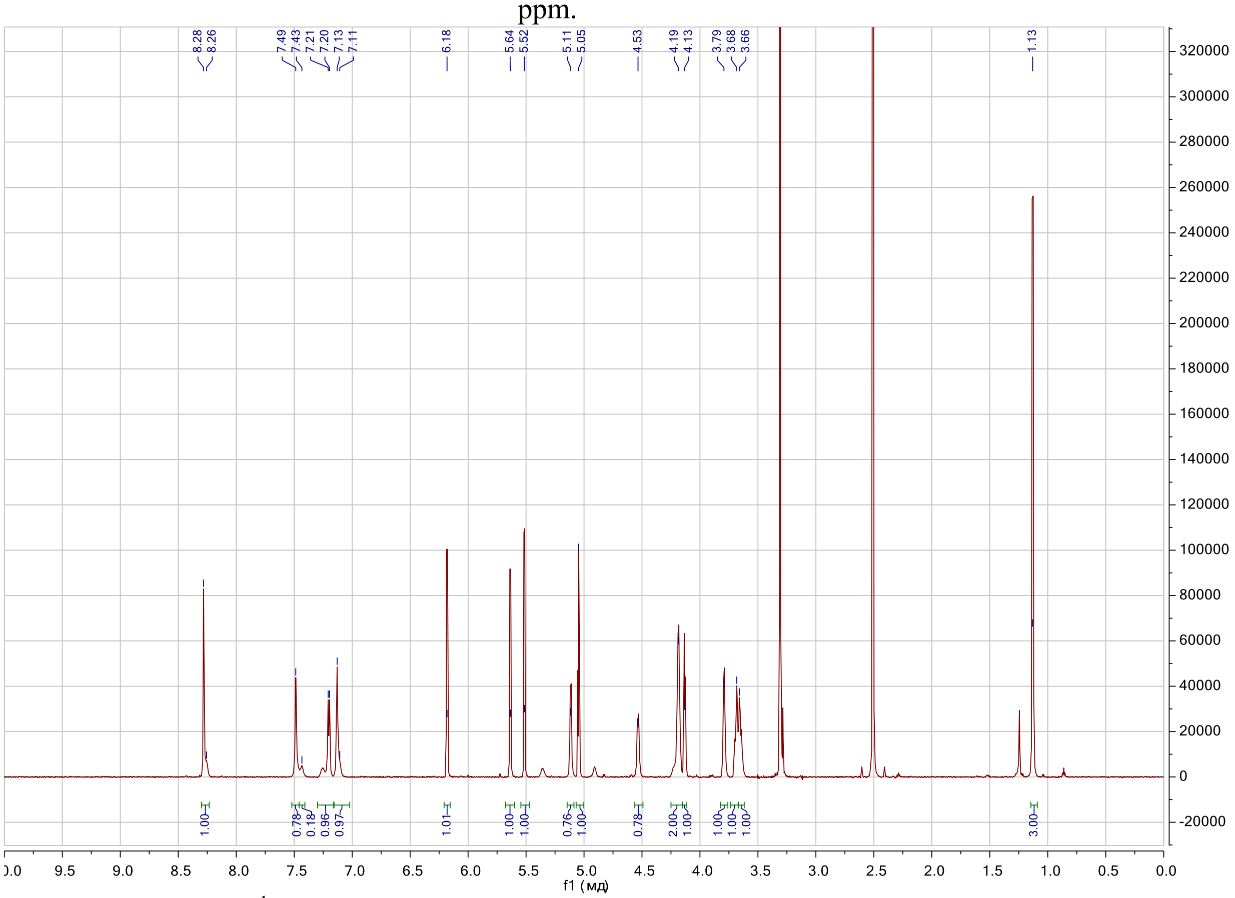

Figure SI-28. The ^{1}H NMR spectrum of 9-*β*-*D*-arabinofuranosyl-2-chloro-6-(N$^{\alpha}$-L-threoninylamido)-purine **(6b)**

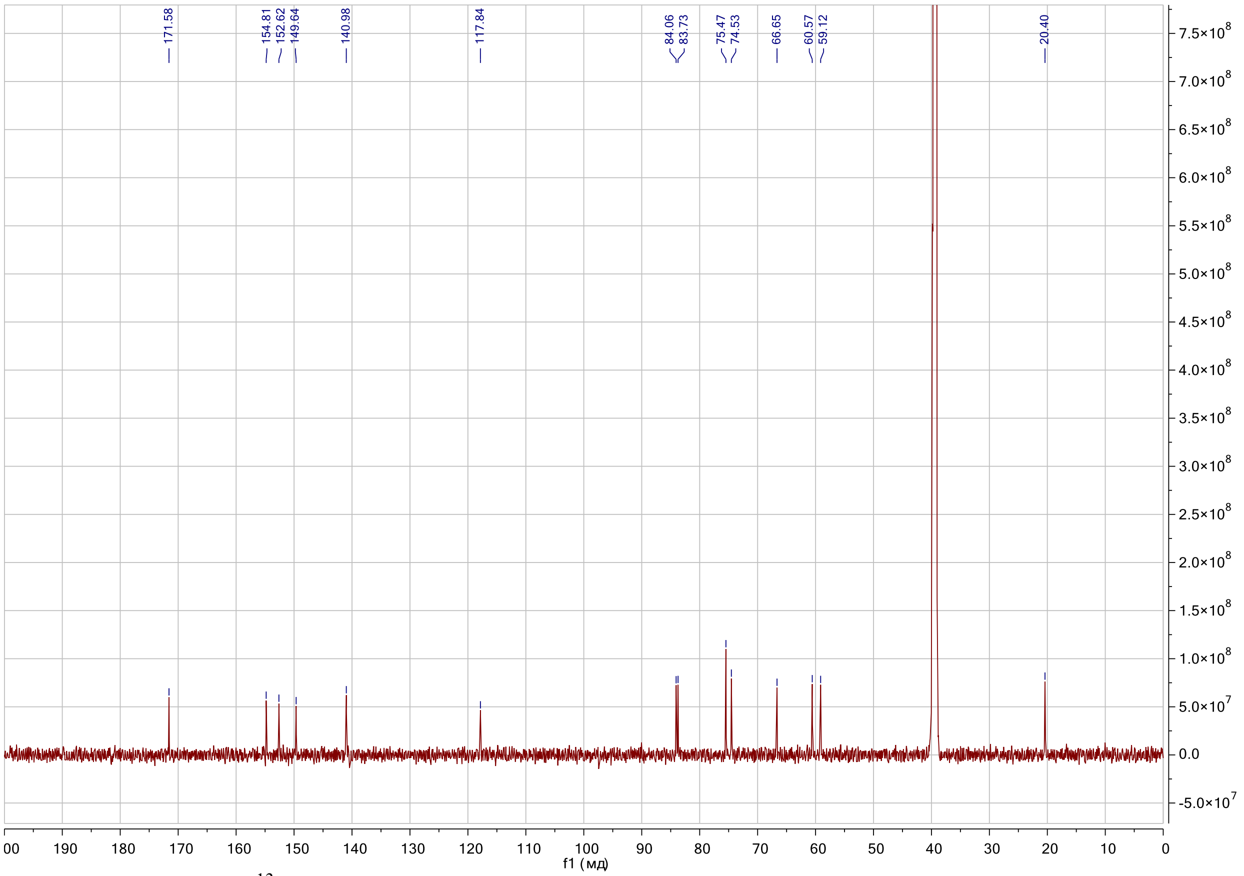

Figure SI-29. The ^{13}C NMR spectrum of 9-*β*-*D*-arabinofuranosyl-2-chloro-6-(N$^{\alpha}$-L-threoninylamido)-purine **(6b)**

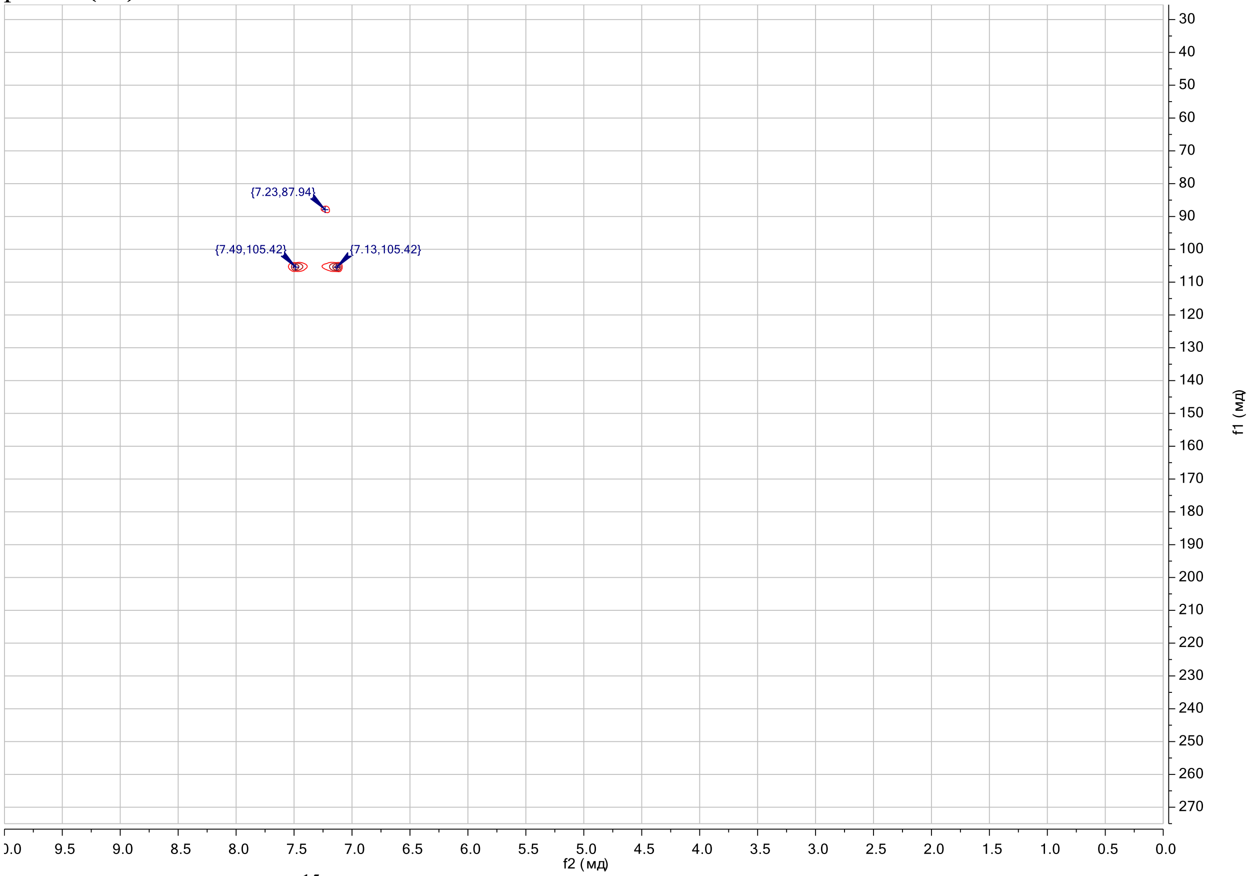

Figure SI-30. The ^{15}N HSQC NMR spectrum of 9-*β*-*D*-arabinofuranosyl-2-chloro-6-(N$^{\alpha}$-L-threoninylamido)-purine **(6b)**

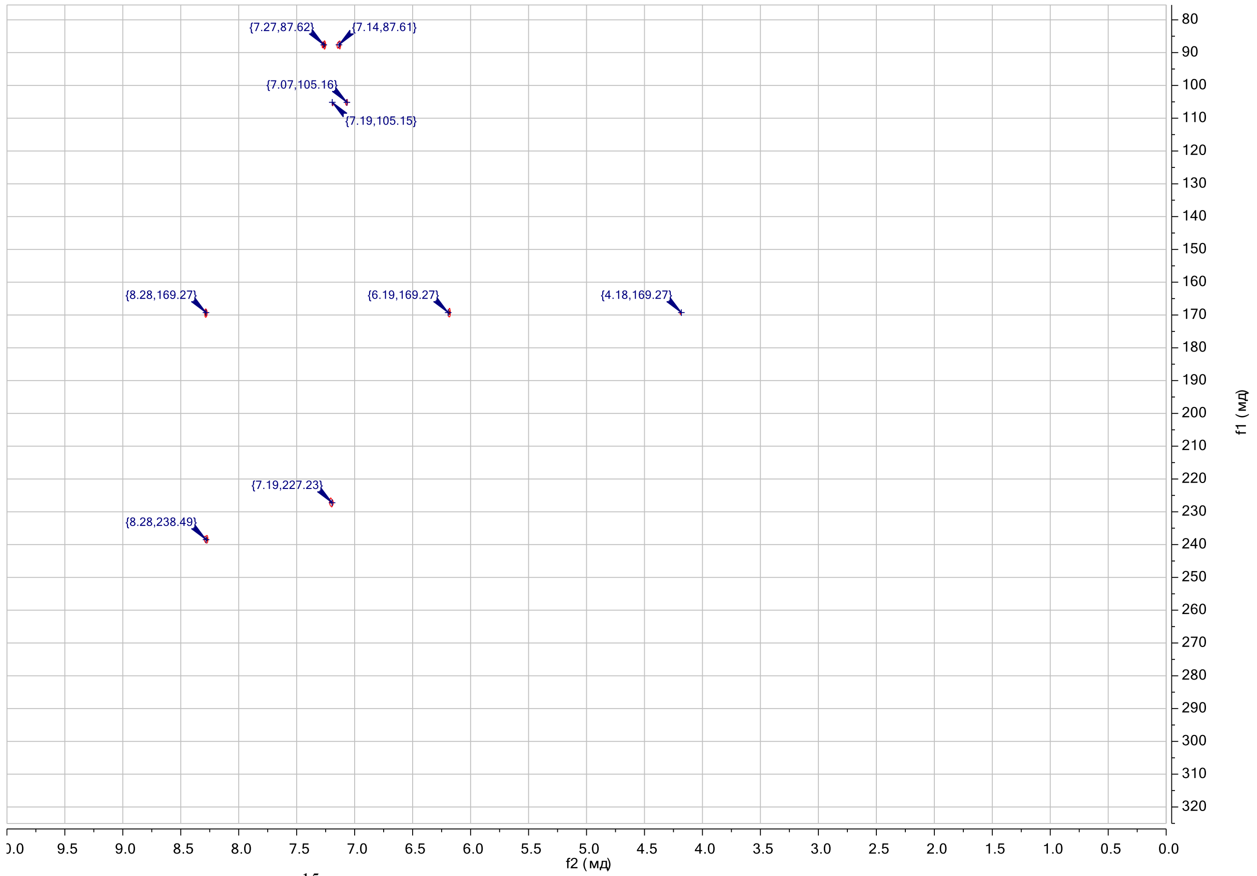

Figure SI-31. The ^{15}N HMBC NMR spectrum of 9-*β*-*D*-arabinofuranosyl-2-chloro-6-(N$^{\alpha}$-L-threoninylamido)-purine **(6b)**

IBCH RAS

Project Name Defaults

Reported by User: Breeze user (Breeze)

SAMPLE INFORMATION

Sample Name:	**6b**	Acquired By:	Breeze
Sample Type:	Unknown	Date Acquired:	05.12.2016 15:06:43 GMT-4
Vial:	1	Acq. Method:	100B_dual_280nm_05ml
Injection #:	1	Date Processed:	15.11.2017 15:55:11 GMT-4
Injection Volume:	10,00 ul	Channel Name:	W2489 ChB
Run Time:	20,00 Minutes	Channel Desc.:	W2489 ChB 280nm
Column Type:		Sample Set Name	

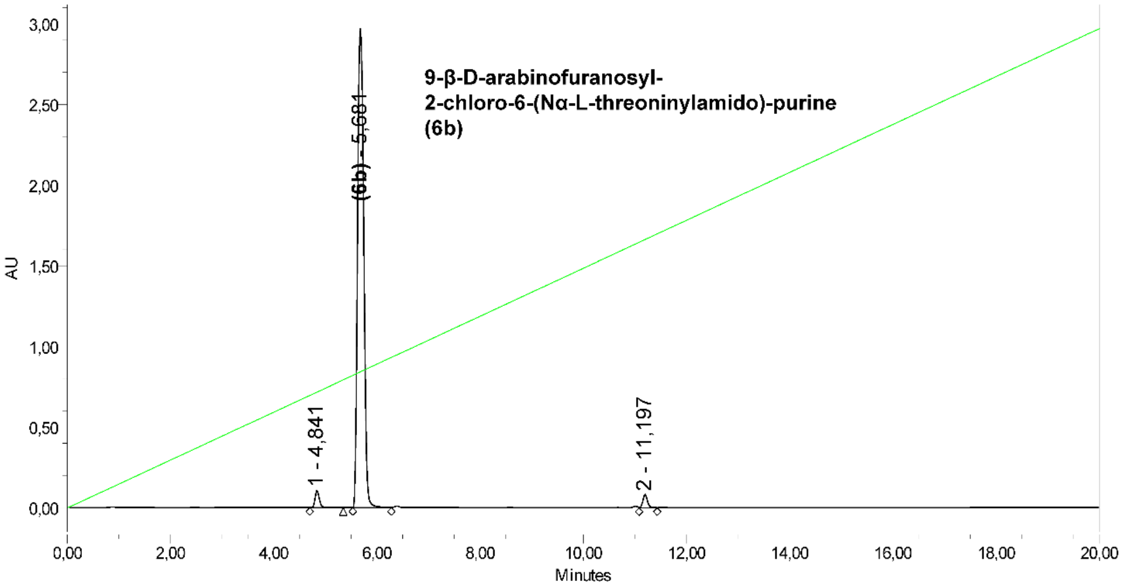

	Peak Name	RT (min)	Area (µV*sec)	% Area	Height (µV)	% Height
1	1	4,841	609045	2,37	104391	3,30
2	**6b**	5,681	24608261	95,73	2974371	94,12
3	2	11,197	487313	1,90	81527	2,58

Report Method: Gradient Overlay Report ASC

Page: 1 of 1

Printed: 15.11.2017 16:06:20 Etc/GMT-4

Figure SI-32. The chromatogram of **6b**

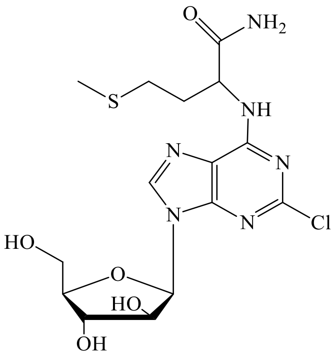

7b

9-*β*-*D*-arabinofuranosyl-2-chloro-6-(N$^{\alpha}$-L-methioninylamido)-purine (7b)

^{1}H NMR (700 MHz, DMSO-d_6, 30 °C): δ= 8.26 (s, 1H, H-8), 8.06 (br.d, 1H *J* = 7.6Hz, C6-NH), 7.45 and 7.42 (2 sign, 0.75H and 0.18H, CO-NH), 7.11 and 7.09 (2 sign, 0.72H and 0.22H, CO-NH), 6.18 (d, *J* =5.17 Hz, 1H, H-1'), 5.63 (br.s, 1H, 2'-OH), 5.51(d, J = 4.47 Hz, 1H, 2'-OH), 5.04 (br.s, 1H, 5'-OH), 4.68 (m, 0.91H, CαH), 4.18 (br.s, 1H, H-2'), 4.13 (m, 1H, H-3'), 3.79 (m, 1H, H-4'), 3.68 (m, 1H, H-5'a), 3.66 (m, 1H, H-5'b), 2.55 (m, 1H, CγHa), 2.51 (m, 1H, CγHb), 2.10 (m, 2H, CβHa and CβHb), 2.05 (m, 3H, CεH) ppm.

^{13}C NMR (176 MHz, DMSO-d_6, 30 °C) δ = 172.88 (CO-NH2), 154.61(C6), 152.56 (C2), 149.70 (C4), 140.72 (C8), 117.80 (C5), 84.06 (C4'), 83.69 (C1'), 75.45 (C2'), 74.58 (C3'), 60.59 (C5'), 53.04 (Cα), 31.26 (Cβ), 29.85 (Cγ), 14.49 (Cε) ppm.

^{15}N NMR (71 MHz, DMSO-d_6, 30 °C) δ= 239.46 (N7), 227.84 (N1), 169.21 (N9), 104.36 (CO-NH2), 94.82 (C6-NH) ppm.

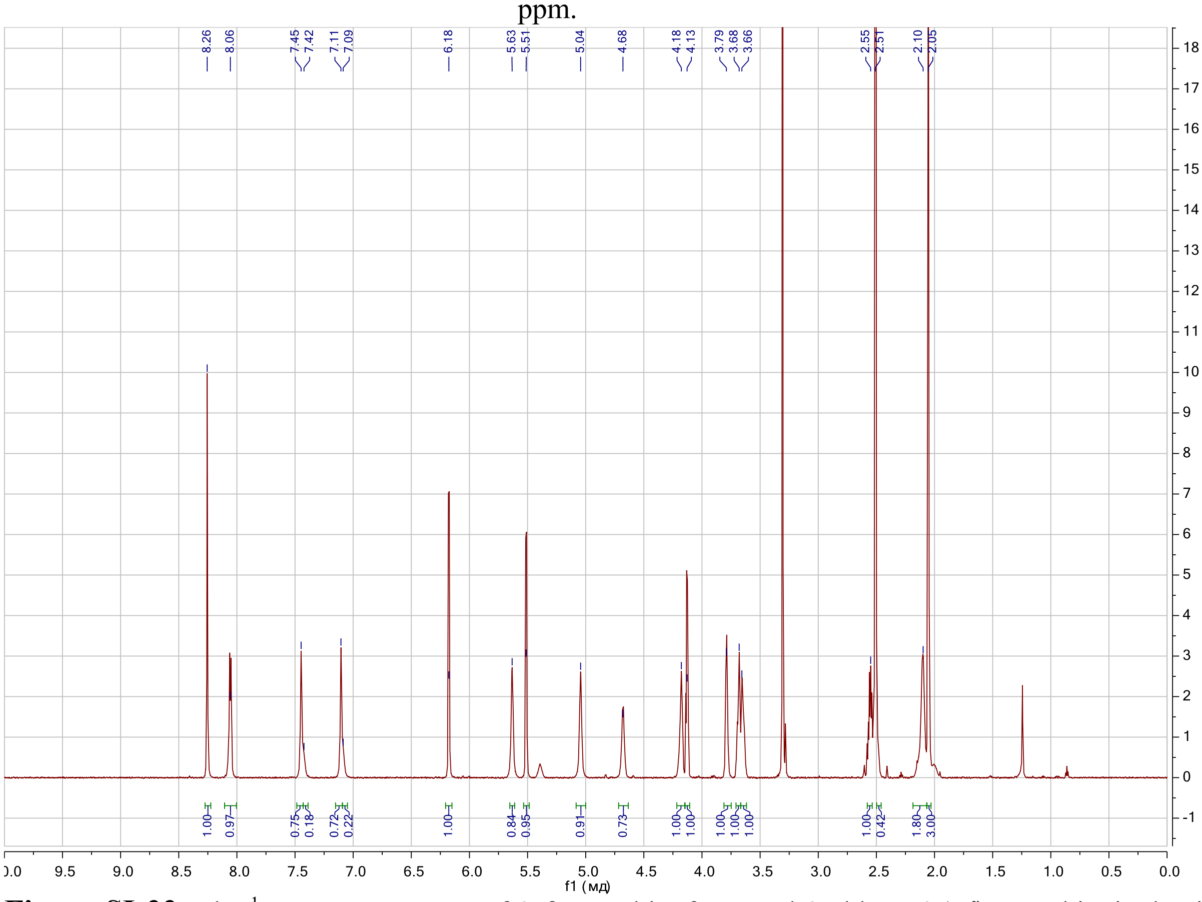

Figure SI-33. The ^{1}H NMR spectrum of 9-*β*-*D*-arabinofuranosyl-2-chloro-6-(N$^{\alpha}$-L-methioninylamido)-purine **(7b)**

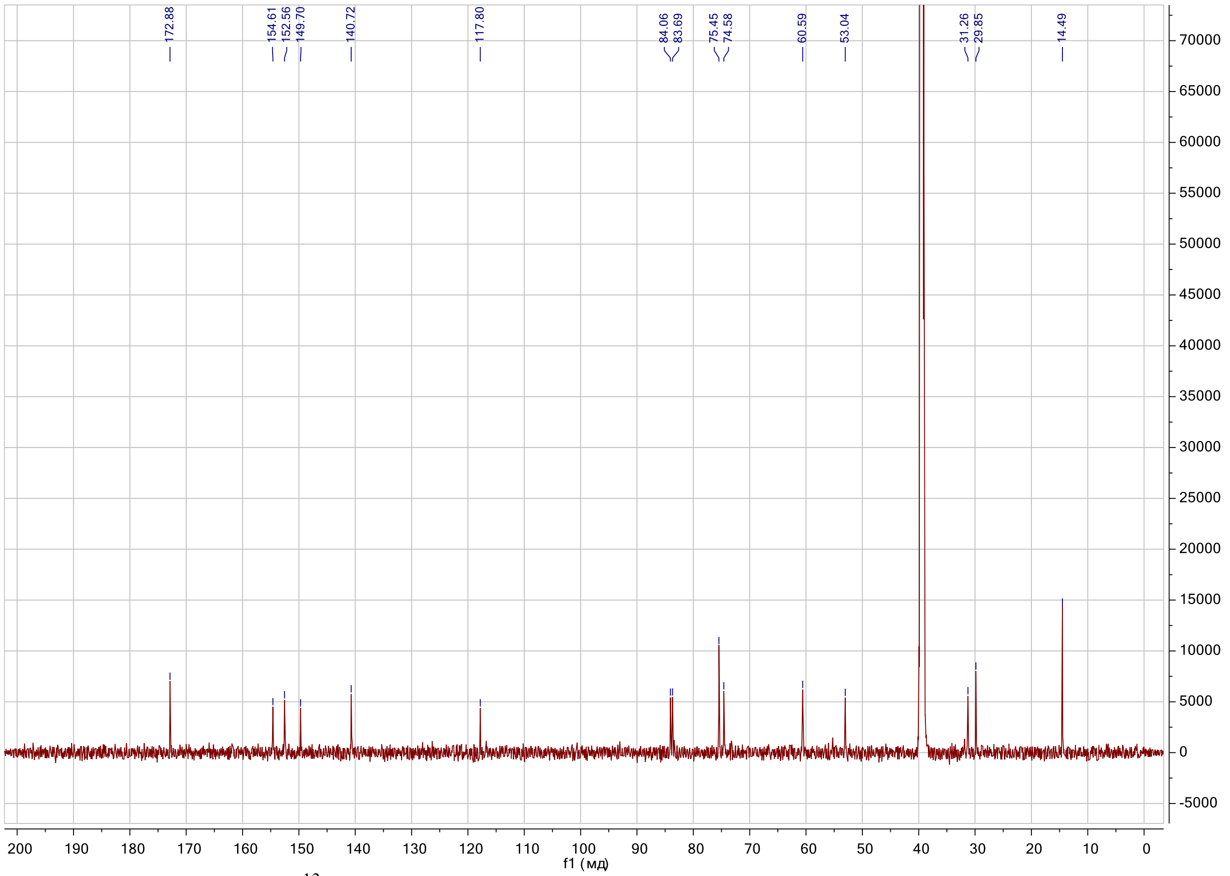

Figure SI-34. The ^{13}C HSQC NMR spectrum of 9-*β*-*D*-arabinofuranosyl-2-chloro-6-(N$^{\alpha}$-L-methioninylamido)-purine **(7b)**

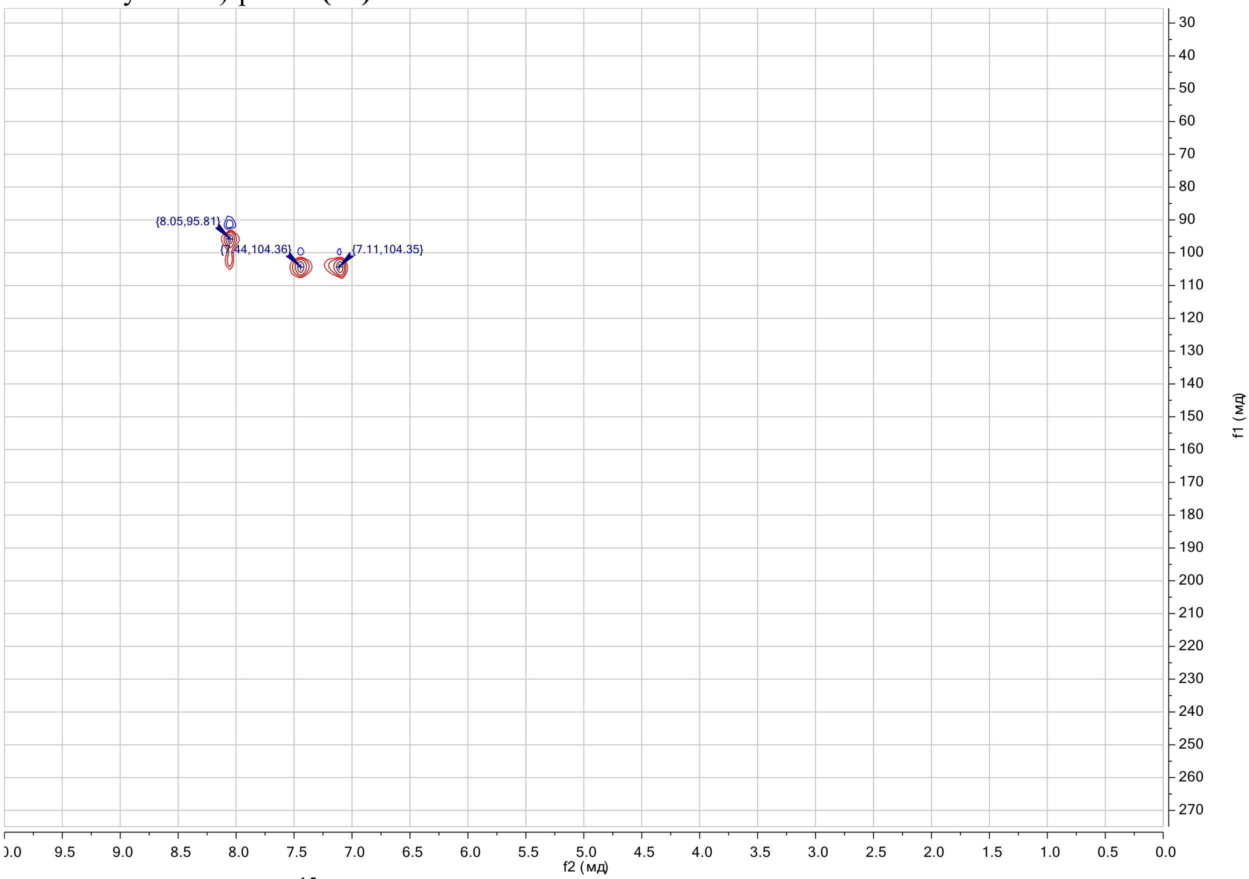

Figure SI-35. The ^{15}N HMBC NMR spectrum of 9-*β*-*D*-arabinofuranosyl-2-chloro-6-(N$^{\alpha}$-L-methioninylamido)-purine **(7b)**

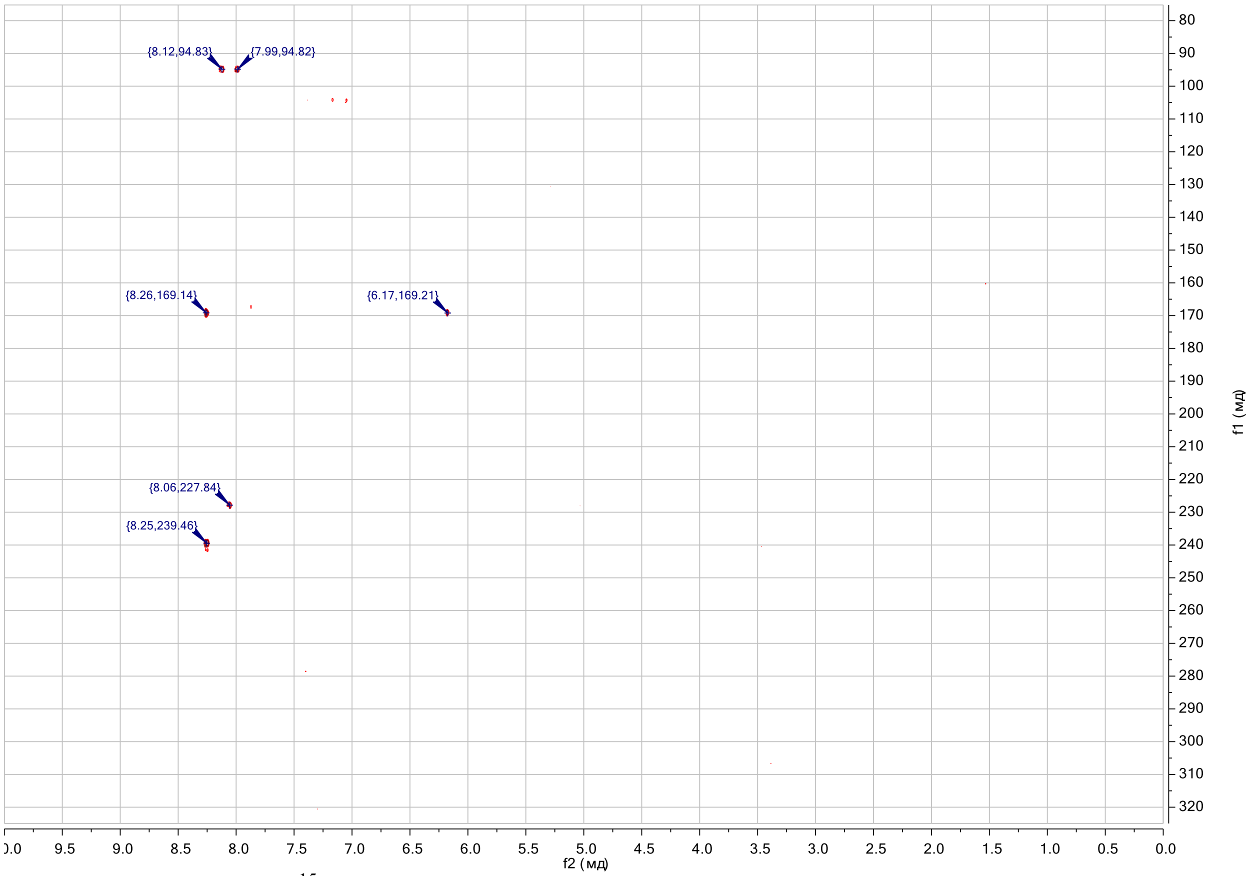

Figure SI-36. The ^{15}N HMBC NMR spectrum of 9-*β*-*D*-arabinofuranosyl-2-chloro-6-(N^{α}-L-methioninylamido)-purine **(7b)**

IBCH RAS

Project Name Defaults

Reported by User: Breeze user (Breeze)

SAMPLE INFORMATION

Sample Name:	**7b**	Acquired By:	Breeze
Sample Type:	Unknown	Date Acquired:	03.06.2016 14:27:08 GMT-4
Vial:	1	Acq. Method:	100B_dual_280nm_05ml
Injection #:	1	Date Processed:	03.06.2016 14:53:37 GMT-4
Injection Volume:	5,00 ul	Channel Name:	W2489 ChA
Run Time:	20,00 Minutes	Channel Desc.:	W2489 ChA 254nm
Column Type:		Sample Set Name	

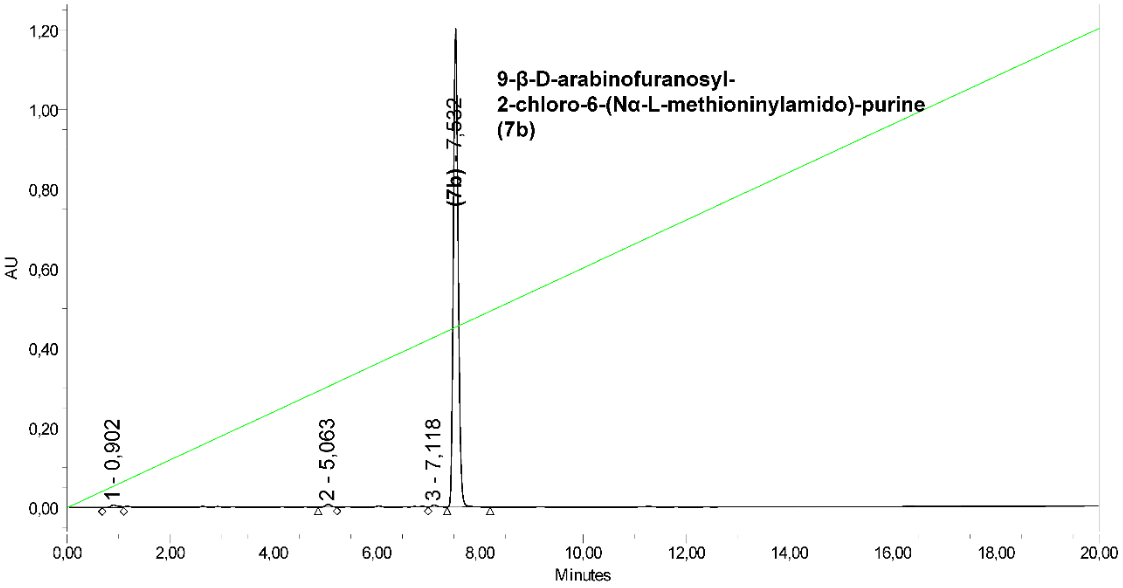

	Peak Name	RT (min)	Area (μV*sec)	% Area	Height (μV)	% Height
1	1	0,902	56890	0,72	6096	0,50
2	2	5,063	49056	0,62	6914	0,57
3	3	7,118	39132	0,49	5544	0,46
4	**7b**	7,532	7779279	98,17	1197225	98,47

Report Method: Gradient Overlay Report ASC

Printed: 03.06.2016 14:54:01 Etc/GMT-4

Page: 1 of 1

Figure SI-37. The chromatogram of **7b**

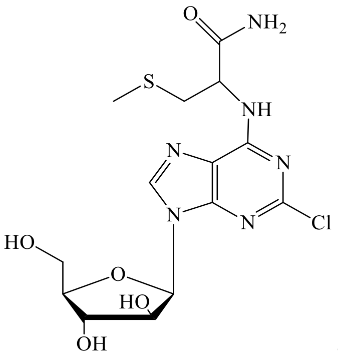

8b

9-*β*-*D*-arabinofuranosyl-2-chloro-6-(N$^{\alpha}$-L-S-methylcysteinylamido)-purine (8b)

^{1}H NMR (700 MHz, DMSO-d_6, 30 C): δ = 8.27 (s, 1H, H-8), 8.05 and 7.94 (2 sign., 0.22H and 0.78H, C6-NH), 7.57 and 7.51 (2 sign., 0.74H and 0.26H, CO-NH), 7.23 (s, 1H, CO-NH), 6.18 (d, *J* = 5.2Hz, 1H, H-1'), 5.65 and 5.60 (2 br.s., 0.70H and 0.30H, 2'-OH), 5.53 (br.s., 1H, 3'-OH), 5.06 (br.s., 1H, 5'-OH), 4.81 (m, 0.8H, NH-C<u>H</u>), 4.19 (br.t., 1H, H-3'), 4.13 (br.t., 1H, H-2'), 3.79 (m, 1H, H-4'), 3.68 (m, 1H, H-5'a), 3.65 (m, 1H, H-5'b), 2.99 and 2.84 (2m, 1.85H and 0.15H,), (m, 0.25H, CH_2), 2.10 and 2.08 (2 s, 2.40H and 0.6H, CH_3) ppm.

^{13}C NMR (176 MHz, DMSO-d_6, 30 C) δ = 171.77 (CO-NH_2), 154.52 (C6), 152.55 (C2), 149.78 (C4), 140.89 (C8), 117.76 (C5), 84.03 (C4'), 83.68 (C1'), 75.47 (C2'), 74.52 (C3'), 60.56, (C5'), 52.90 (NH-CH), 35.75 (CH2-S), 15.00 (S-CH_3) ppm.

^{15}N NMR (71 MHz, DMSO-d_6, 30 C): δ= 238.63 (N7), 227.45 (N1), 169.46 (N9), 105.74 (CO-NH_2), , 95.33 (C6-NH) ppm.

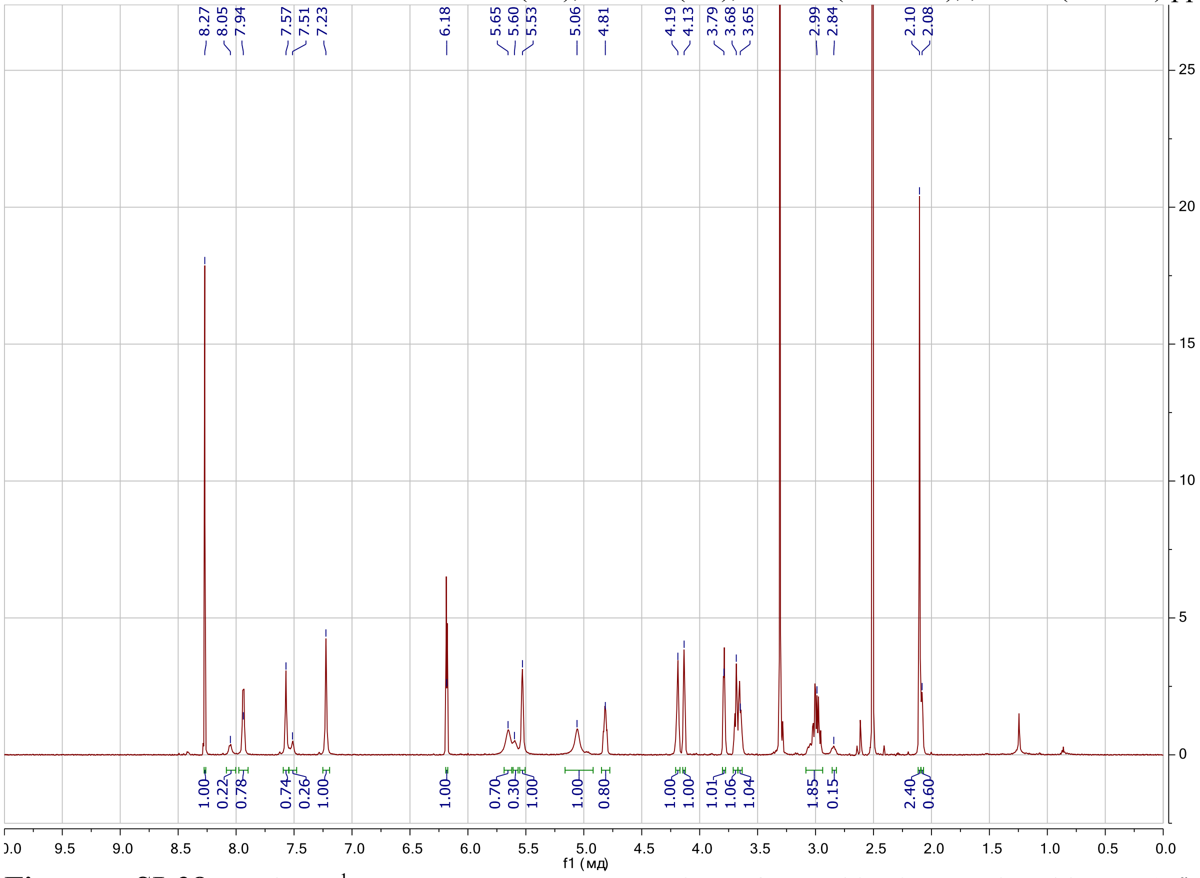

Figure SI-38. The ^{1}H NMR spectrum of 9-*β*-*D*-arabinofuranosyl-2-chloro-6-(N$^{\alpha}$-L-S-methylcysteinylamido)-purine **(8b)**

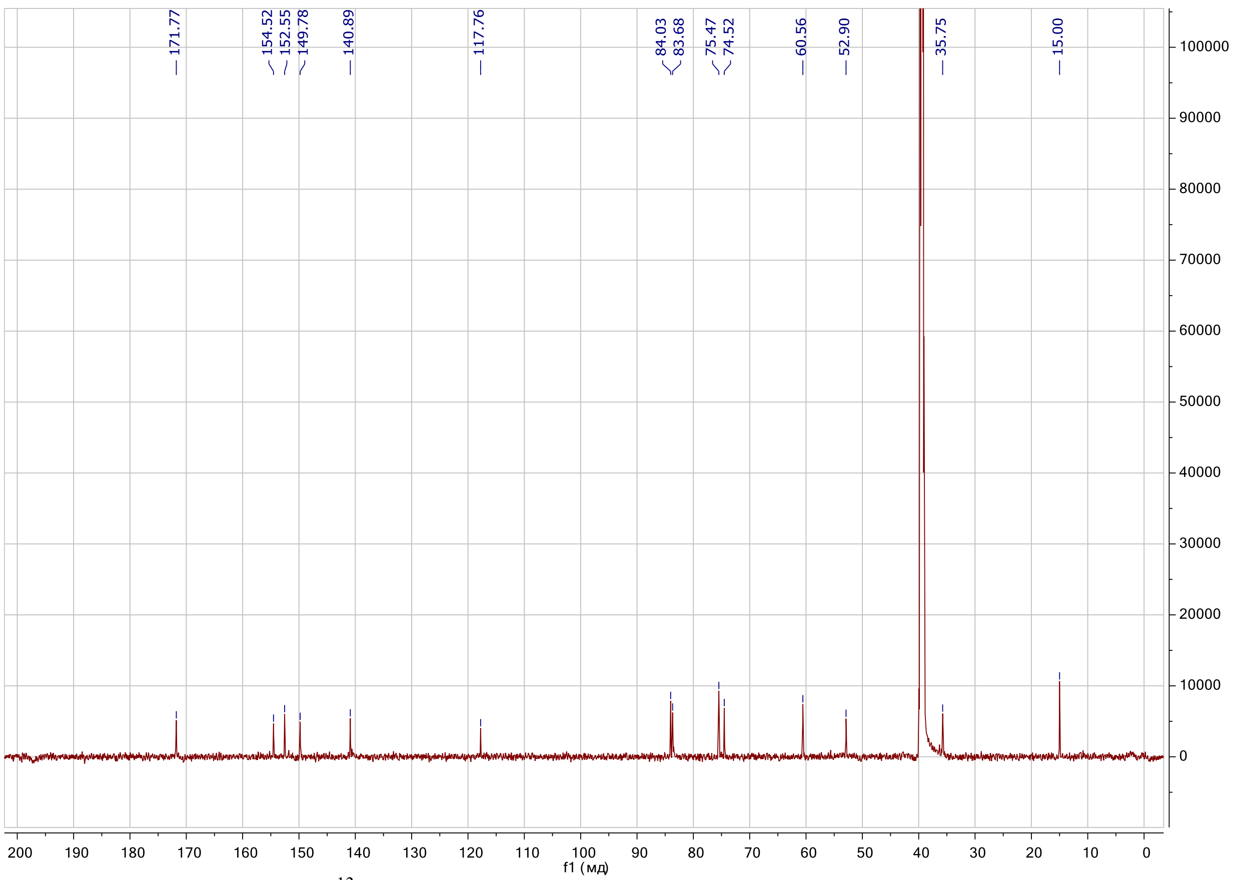

Figure SI-39. The ^{13}C NMR spectrum of 9-*β*-*D*-arabinofuranosyl-2-chloro-6-(N$^{\alpha}$-L-S-methylcysteinylamido)-purine **(8b)**

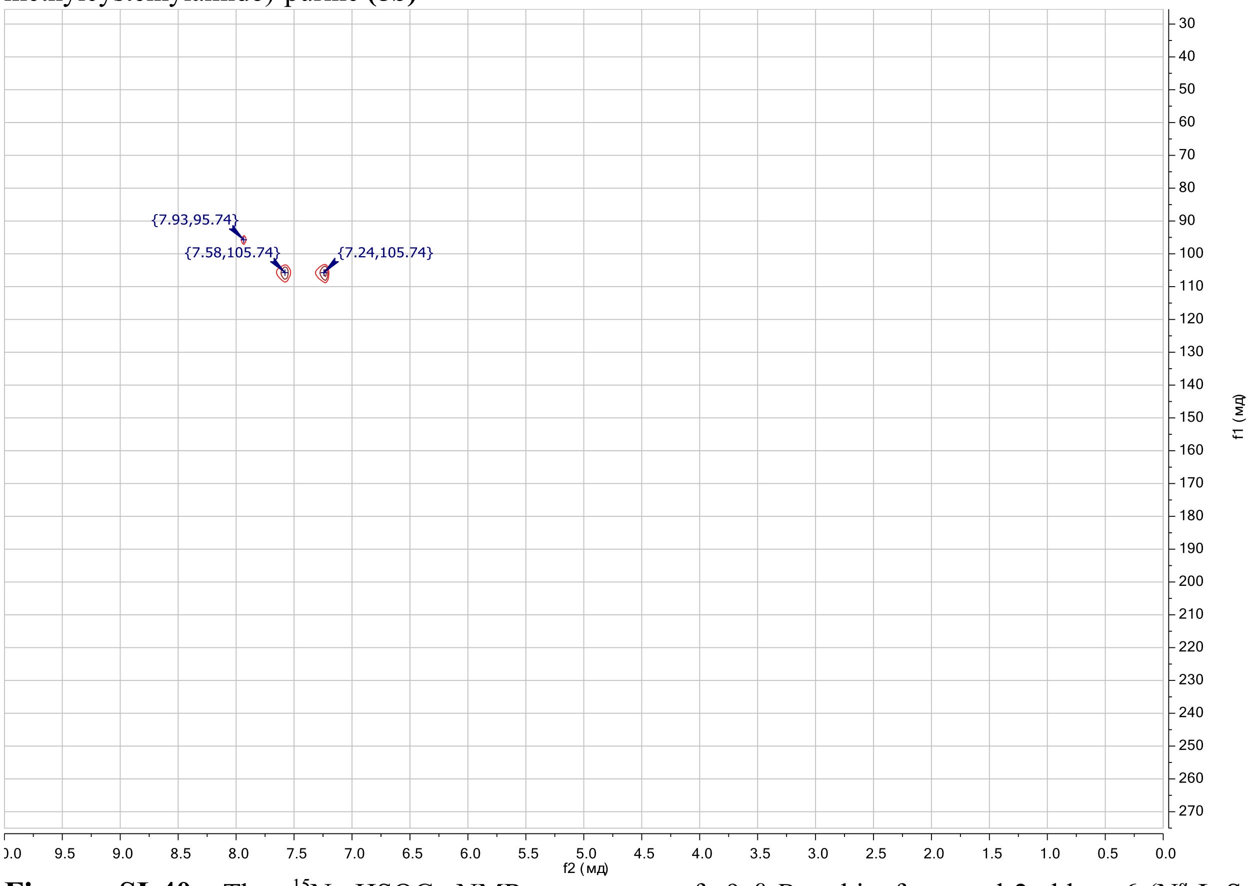

Figure SI-40. The ^{15}N HSQC NMR spectrum of 9-*β*-*D*-arabinofuranosyl-2-chloro-6-(N$^{\alpha}$-L-S-methylcysteinylamido)-purine **(8b)**

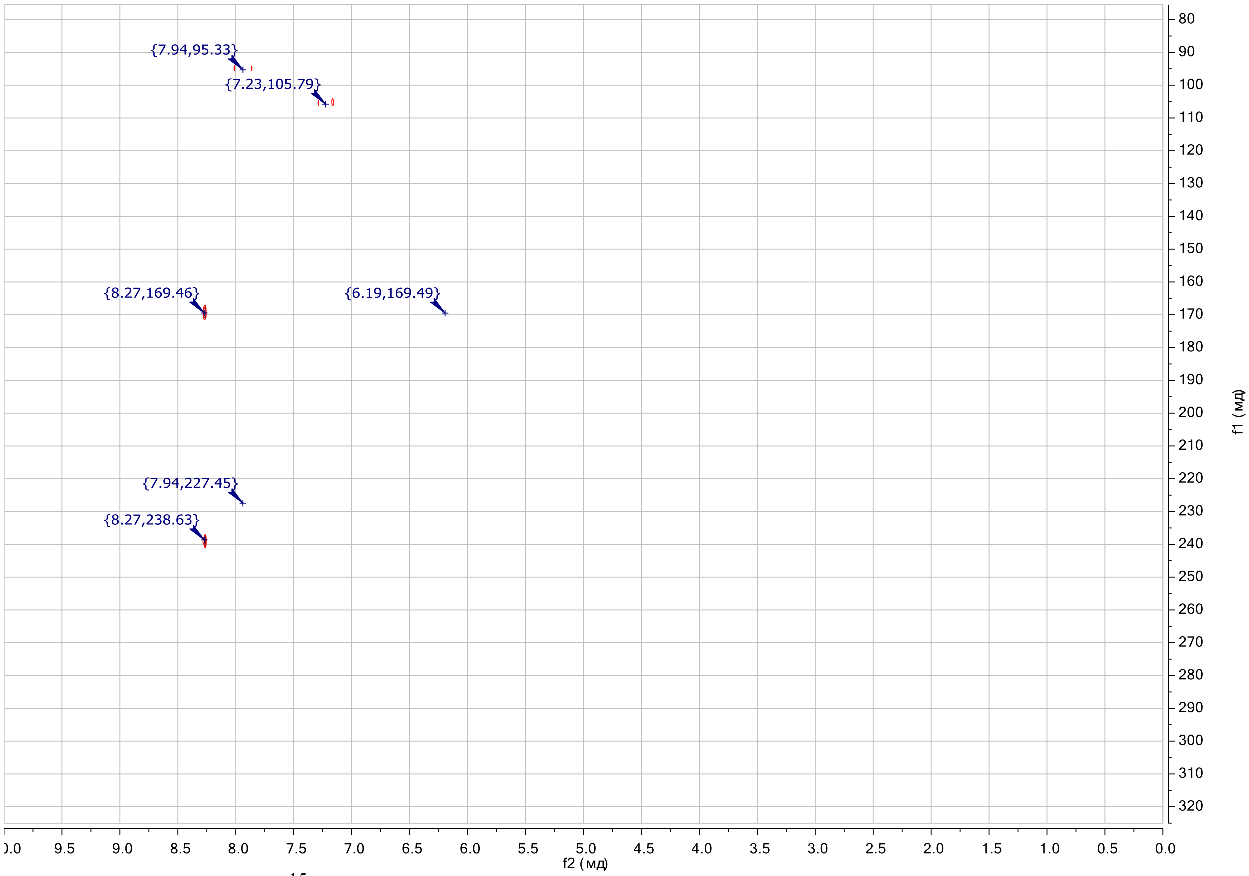

Figure SI-41. The ^{15}N HMBC NMR spectrum of 9-*β*-*D*-arabinofuranosyl-2-chloro-6-(N$^{\alpha}$-L-S-methylcysteinylamido)-purine **(8b)**

IBCH RAS

Project Name Defaults

Reported by User: Breeze user (Breeze)

Breeze 2 HPLC System

SAMPLE INFORMATION

Sample Name:	**8b**	Acquired By:	Breeze
Sample Type:	Unknown	Date Acquired:	03.06.2016 15:25:36 GMT-4
Vial:	1	Acq. Method:	100B_dual_280nm_05ml
Injection #:	3	Date Processed:	03.06.2016 15:47:12 GMT-4
Injection Volume:	5,00 ul	Channel Name:	W2489 ChB
Run Time:	20,00 Minutes	Channel Desc.:	W2489 ChB 280nm
Column Type:		Sample Set Name	

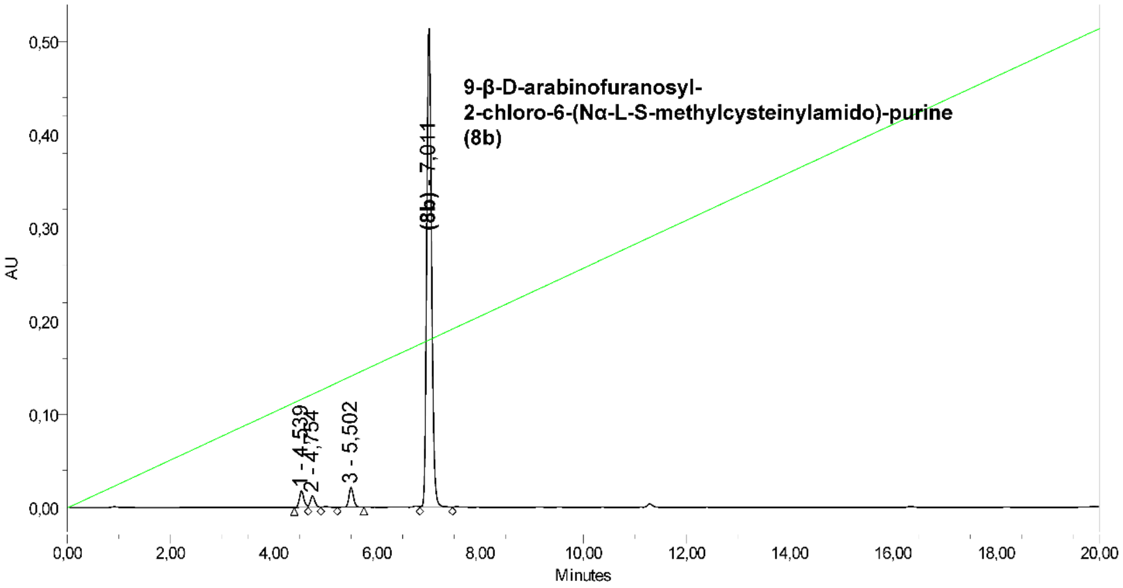

	Peak Name	RT (min)	Area (μV*sec)	% Area	Height (μV)	% Height
1	1	4,539	107862	2,94	17527	3,10
2	2	4,754	76401	2,08	12209	2,16
3	3	5,502	130571	3,56	21083	3,73
4	**8b**	7,011	3356839	91,43	514293	91,01

Report Method: Gradient Overlay Report ASC   Printed: 03.06.2016 15:47:59 Etc/GMT-4

Page: 1 of 1

Figure SI-42. The chromatogram of **8b**

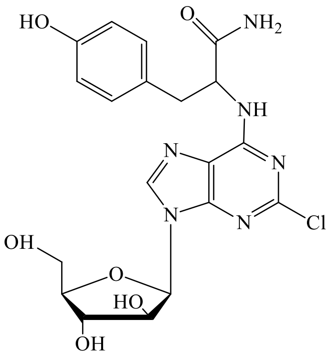

9b

9-*β*-*D*-arabinofuranosyl-2-chloro-6-(N$^{\alpha}$-L-tyrosinylamido)-purine (9b)

^{1}H NMR (700 MHz, DMSO-d_6, 30 C): δ = 9.14 (s, 1H, OH), 8.40 (s, 1H, H-8), 8.04 and 7.88 (br.d, *J* = 9.6 Hz, 0.20H and d, J = 8.0 Hz, 0.71H, CO-NH), 7.57 and 7.50 (2 sign, 0.69H and 0.20H, CO-NH) 7.15 (br.sign, 0.69H, CO-NH) 7.12 and 7.08 (m, J = 8.5 Hz, J = 8.2 Hz, 2H, CδH), 6.62 and 6.61 (m, 2H, CεH), 5.81 (d, J = 6.0 Hz, 1H, H-1'), 5.47 (d, J = 6.14 Hz, 1H, 3'-OH), 5.18 (br.d, J = 5.0 Hz, 1H, 2'-OH), 5.04 (m, 1H, 5'-OH), 4.76 (m, 0.74H, CαH), 4.51 (m, 1H, H-3'), 4.34 (d, J = 4.22 Hz, 0.12H,), 4.13 (m, 1H, H-2') 3.94 (m, 1H, H-4'), 3.65 (m, 1H, H-5'a), 3.55 (m, 1H, H-5'b) 3.07 (m, 0.8H, CβHa) 3.01(m, 1.2H, CβHb) ppm.

^{13}C NMR (176 MHz, DMSO-d_6, 30 C) δ = 172.69 (CO-NH$_2$), 155.66 (Cζ) 154.55 (C6), 152.71 (C2), 149.55 (C4), 140.07 (C8), 129.97 (Cδ), 127.90 (C γ), 118.54 (C5), 114.77 (Cε), 87.36 (C1'), 85.61 (C4'), 73.56 (C3'), 70.26 (C2'), 61.23 (C5'), 55.36 (Cα), 36.27 (Cβ) ppm.

^{15}N NMR (71 MHz, DMSO-d_6, 30 C): δ= 241.15 (N7), 227.65 (N1), 171.60 (N9), 104.48 (CO-NH$_2$), 95.79 (C6-NH) ppm.

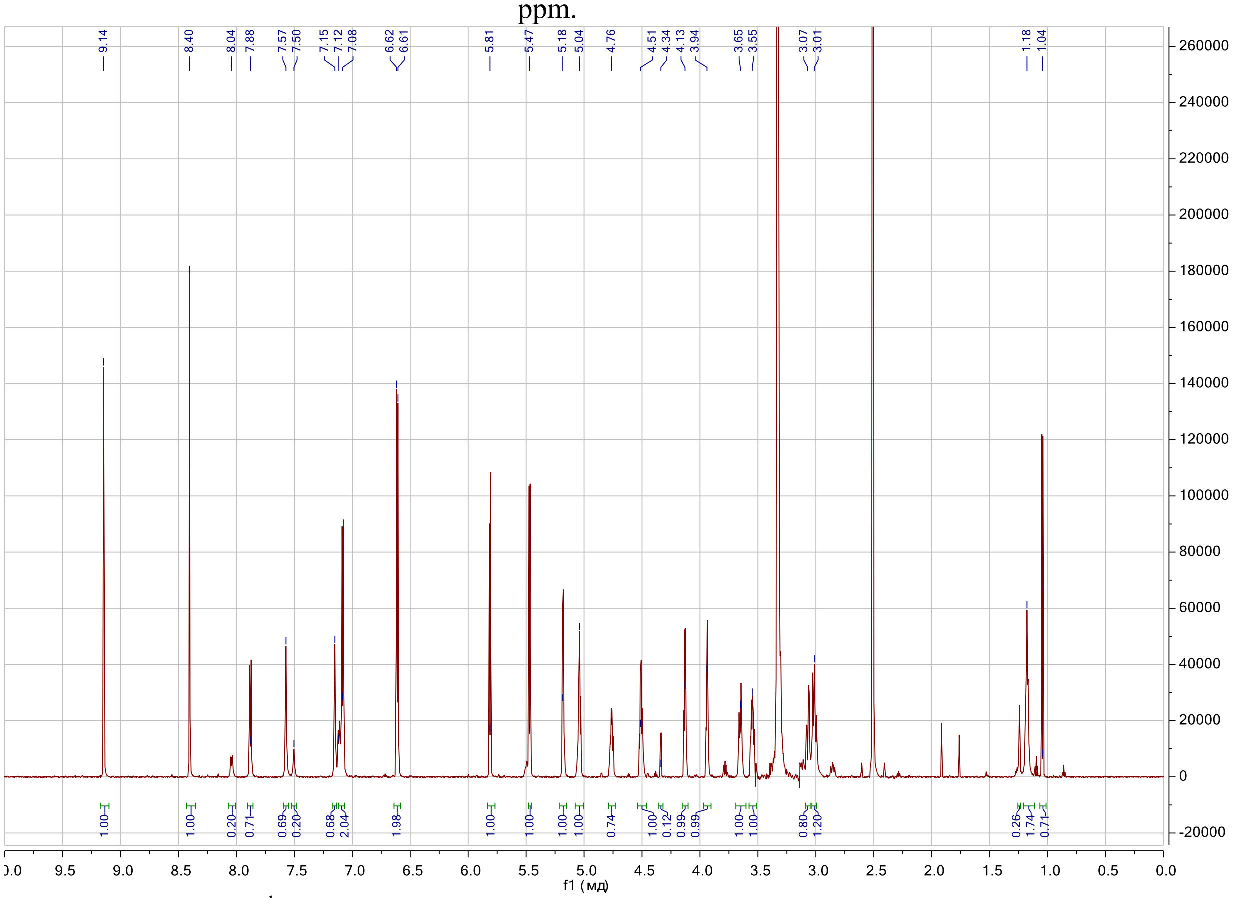

Figure SI-43. The ^{1}H NMR spectrum of 9-*β*-*D*-arabinofuranosyl-2-chloro-6-(N$^{\alpha}$-L-tyrosinylamido)-purine **(9b)**

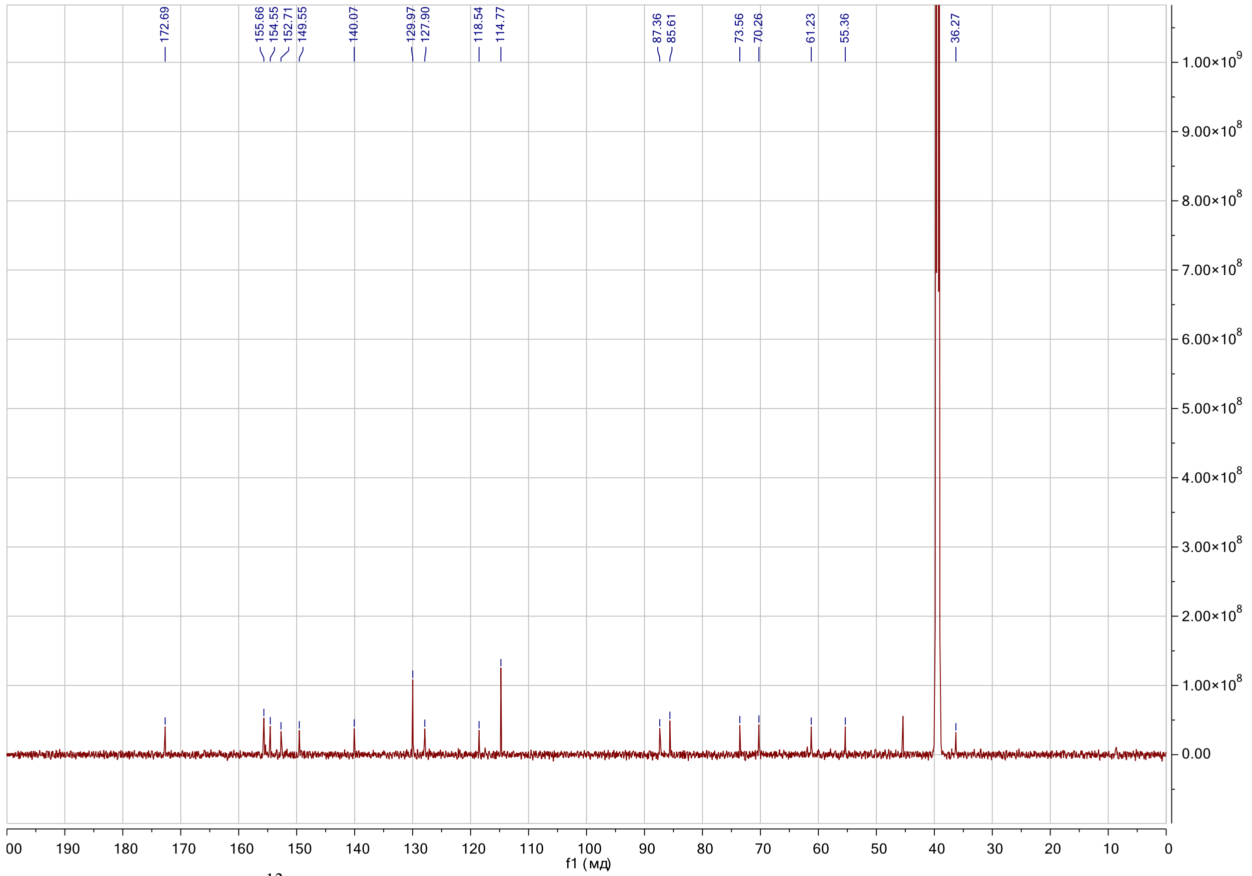

Figure SI-44. The ^{13}C NMR spectrum of 9-*β*-*D*-arabinofuranosyl-2-chloro-6-(N$^{\alpha}$-L-tyrosinylamido)-purine **(9b)**

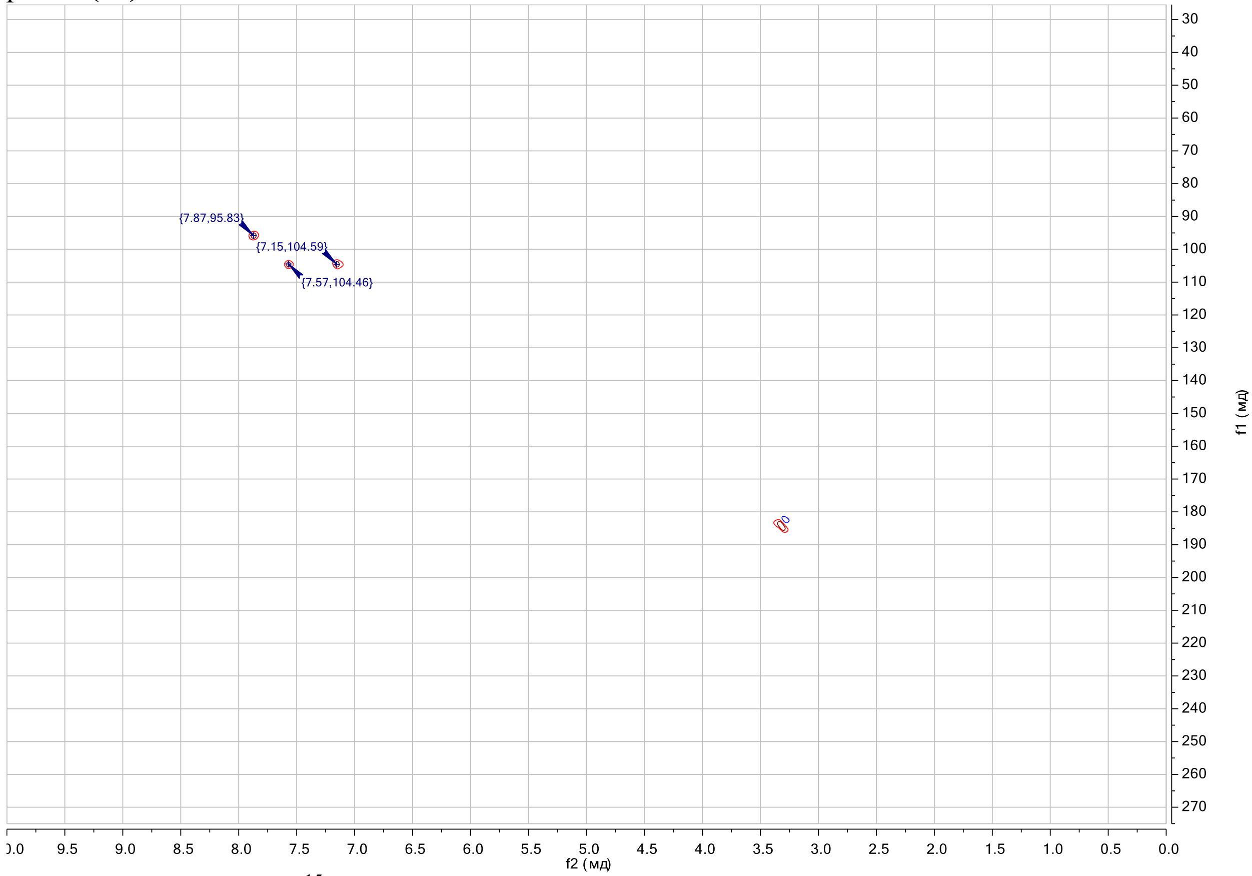

Figure SI-45. The ^{15}N HSQC NMR spectrum of 9-*β*-*D*-arabinofuranosyl-2-chloro-6-(N$^{\alpha}$-L-tyrosinylamido)-purine **(9b)**

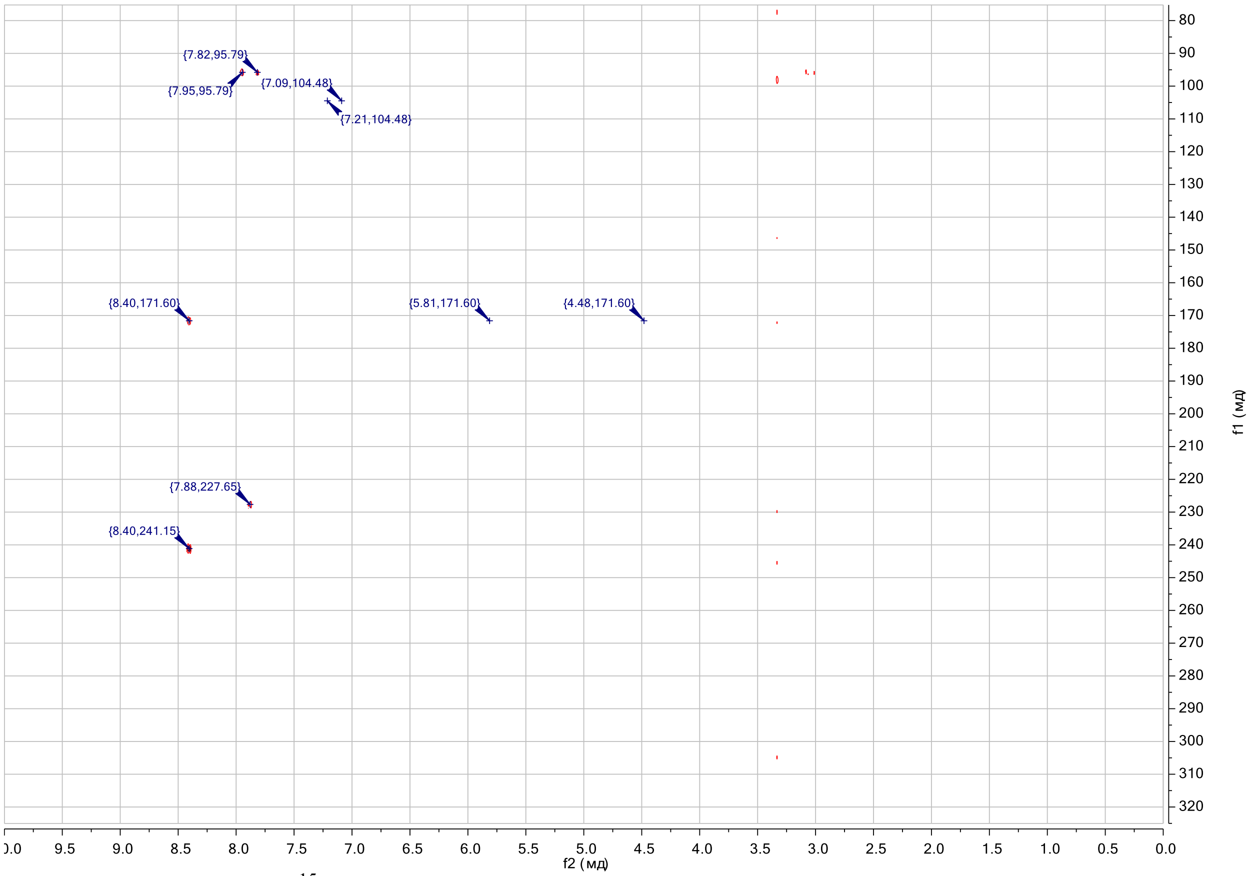

Figure SI-46. The ^{15}N HMBC NMR spectrum of 9-*β*-*D*-arabinofuranosyl-2-chloro-6-(N^{α}-L-tyrosinylamido)-purine **(9b)**

IBCH RAS

Project Name Defaults

Reported by User: Breeze user (Breeze)

SAMPLE INFORMATION

Sample Name:	**9b**	Acquired By:	Breeze
Sample Type:	Unknown	Date Acquired:	19.01.2017 13:21:21 GMT-4
Vial:	1	Acq. Method:	100B_dual_280nm_05ml
Injection #:	1	Date Processed:	15.11.2017 16:09:45 GMT-4
Injection Volume:	10,00 ul	Channel Name:	W2489 ChB
Run Time:	20,00 Minutes	Channel Desc.:	W2489 ChB 280nm
Column Type:		Sample Set Name	

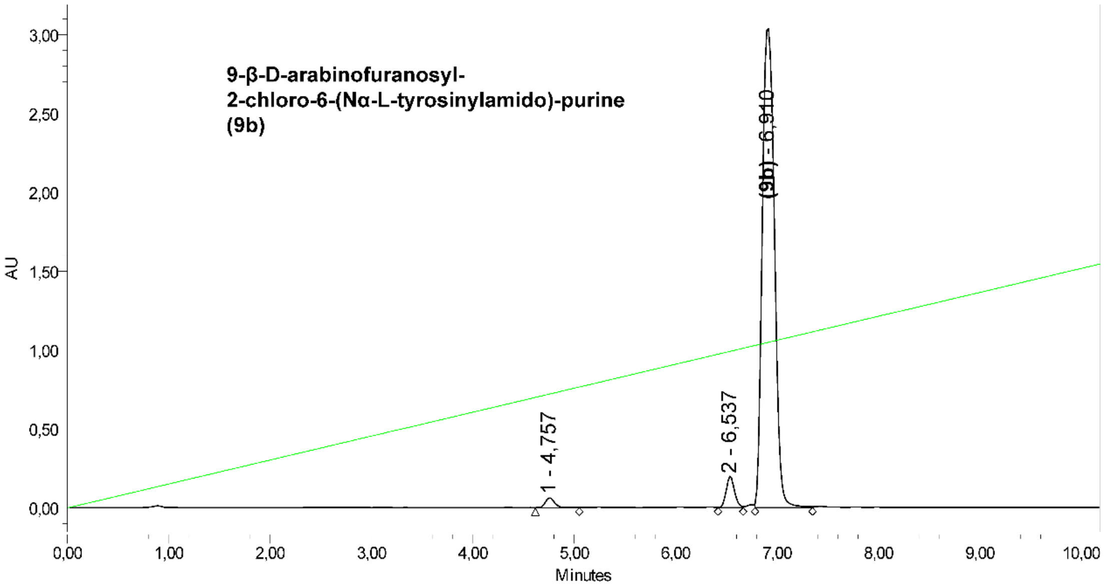

	Peak Name	RT (min)	Area (µV*sec)	% Area	Height (µV)	% Height
1	1	4,757	387879	1,44	61277	1,85
2	2	6,537	1121292	4,17	197253	5,96
3	**9b**	6,910	25367263	94,38	3053608	92,19

Report Method: Gradient Overlay Report ASC Printed: 15.11.2017 16:10:13 Etc/GMT-4

Page: 1 of 1

Figure SI-47. The chromatogram of **9b**

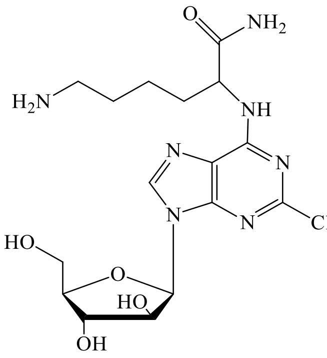

10b

9-*β*-*D*-arabinofuranosyl-2-chloro-6-(N$^{\alpha}$-L-lysinylamido)-purine (10b)

^{1}H NMR (700 MHz, DMSO-d_6, 30 °C): δ= 8.26 (s, 1H, H-8), 7.97 and 7.88 (br.d, 0.18H and d, *J* = 8Hz, 0.72H, C6-NH), 7.61 (br.sign, 2H, Cε-NH_2), 7.46 and 7.39 (2 br.sign, 0.80H and 0.20H, CO-NH), 7.12 and 7.07 (2 br.sign, 0.78H and 0.22H, CO-NH), 6.17 (d, *J* = 5.1Hz, 1H, H-1'), 5.67 and 5.65 (br.sign, 0.2H, and d, *J* = 5.5 Hz, 0.73H, 2'-OH), 5.52 (d, *J* = 4.8Hz, 0.91H, 3'-OH), 5.09 and 5.03 (br.sign, 0.20H, and t, *J* = 5.2Hz, 0.75H, 5'-OH), 4.59 (br.sign, 0.81H, CαH), 4.22 and 4.18 (br.sign, 0.22H and m, 0.78H, H-2'), 4.13 (m, 1H, H-3'), 3.79 (m, 1H, H-4'), 3.69 (m, 1H, H-5'a), 3.65 (m, 1H, H-5'b), 2.81 (m, 2H, Cβ-Ha and Cβ-Hb), 1.85 and 1.74 (2 br.sign, 1.78 H and 0.22H, Cε-Ha and Cε-Hb), 1.56 (m, 2H, Cδ-Ha and Cδ-Hb), 1.40 (m, 2H, Cγ-Ha and Cγ-Hb) ppm.

^{13}C NMR (176 MHz, DMSO-d_6, 30 °C) δ = 173.17 (CO-NH_2), 154.56 (C6), 152.63 (C2), 149.68 (C4), 140.43 (C8), 117.77 (C5), 83.9 (C1'), 83.59 (C4'), 75.24 (C2'), 74.38 (C3'), 60.33 (C5'), 53.14 (Cα), 38.37 (Cβ), 30.90 (Cε), 26.33 (Cδ), 22.08 (Cγ) ppm.

^{15}N NMR (71 MHz, DMSO-d_6, 30 °C): δ= 239.70 (N7), 169.77 (N9), 104.36 (CO-NH_2), 95.87 (C6-NH) ppm.

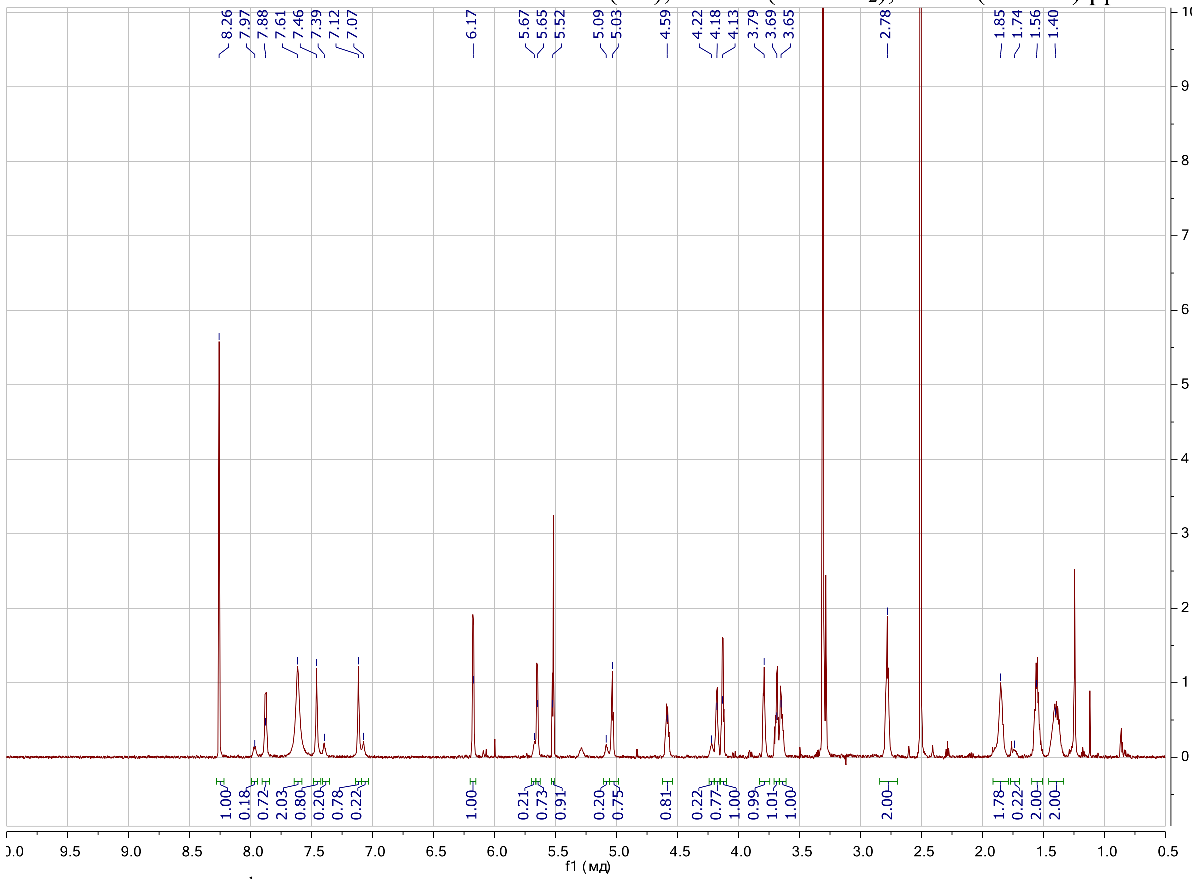

Figure SI-48. The ^{1}H NMR spectrum of 9-*β*-*D*-arabinofuranosyl-2-chloro-6-(N$^{\alpha}$-L-lysinylamido)-purine **(10b)**

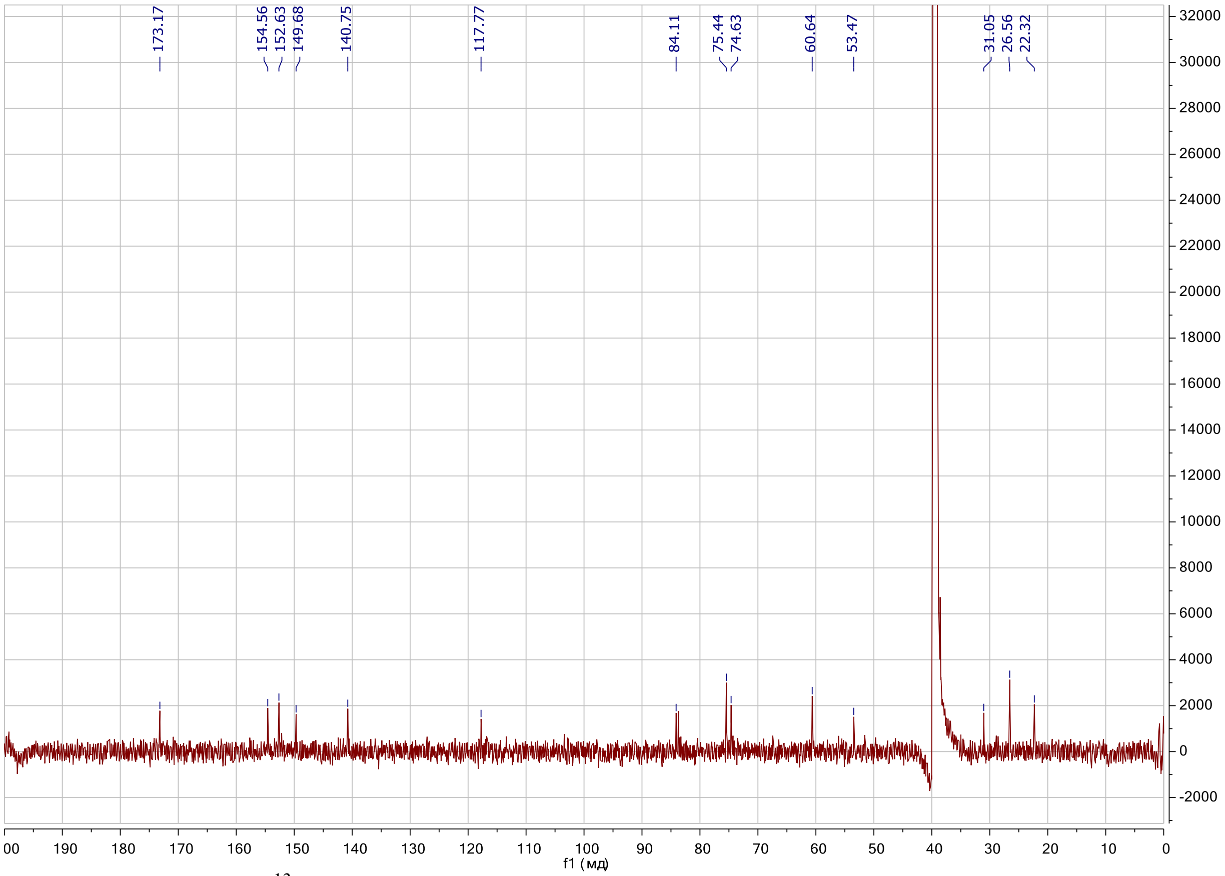

Figure SI-49. The ^{13}C NMR spectrum of 9-*β*-*D*-arabinofuranosyl-2-chloro-6-(N$^{\alpha}$-L-lysinylamido)-purine **(10b)**

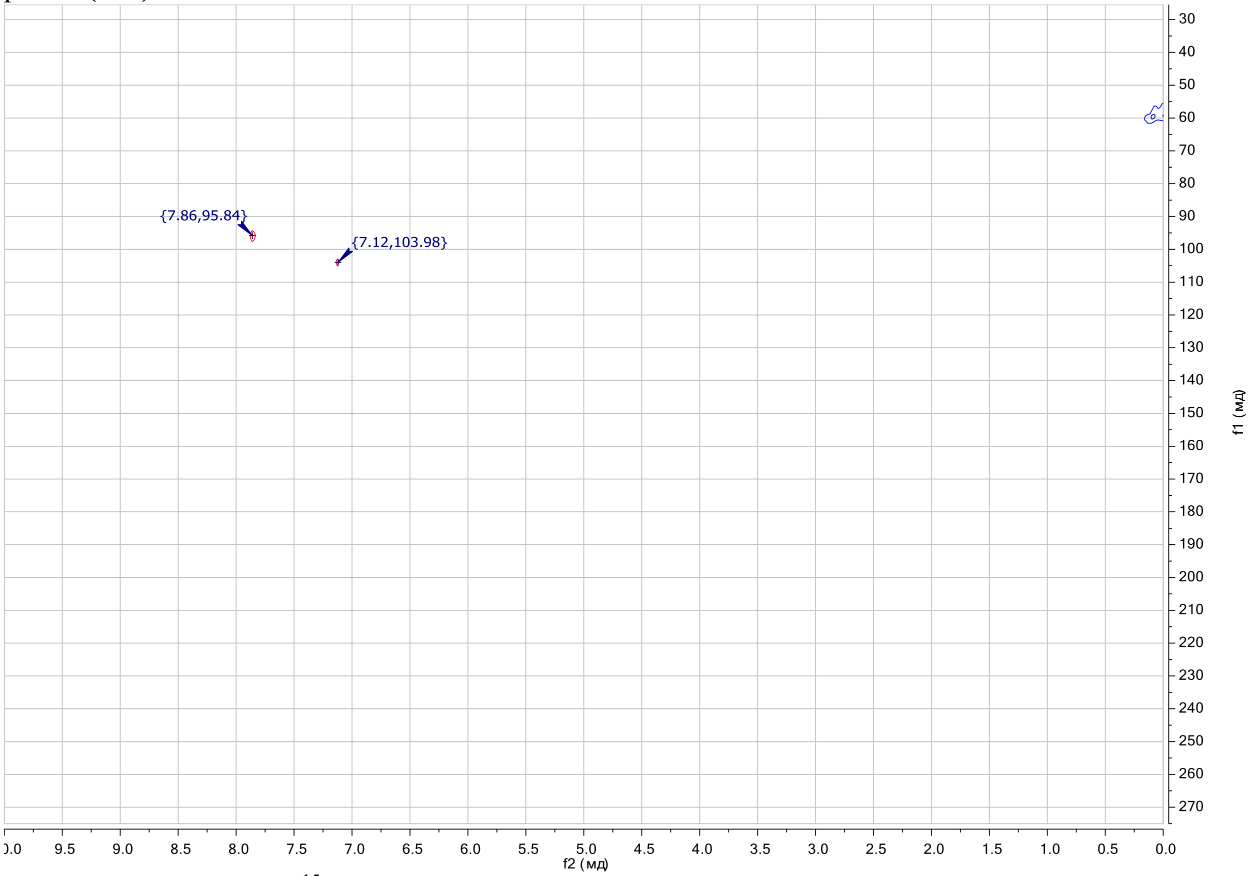

Figure SI-50. The ^{15}N HSQC NMR spectrum of 9-*β*-*D*-arabinofuranosyl-2-chloro-6-(N$^{\alpha}$-L-lysinylamido)-purine **(10b)**

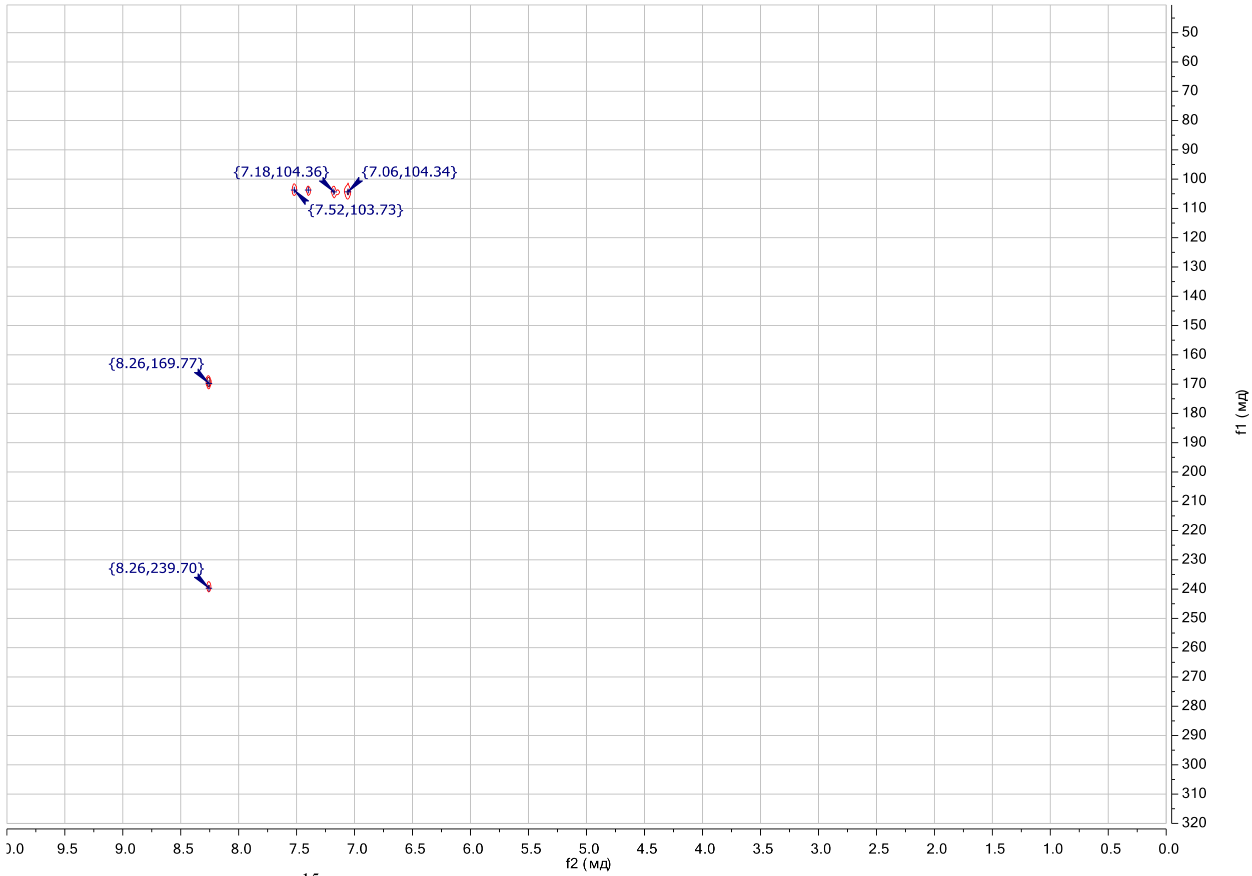

Figure SI-51. The ^{15}N HMBC NMR spectrum of 9-*β*-*D*-arabinofuranosyl-2-chloro-6-(N$^{\alpha}$-L-lysinylamido)-purine **(10b)**

IBCH RAS

Project Name Defaults

Reported by User: Breeze user (Breeze)

S A M P L E I N F O R M A T I O N

Sample Name:	**10b**	Acquired By:	Breeze
Sample Type:	Unknown	Date Acquired:	20.08.2018 15:02:23 GMT-4
Vial:	1	Acq. Method:	100B_dual_280nm_05ml
Injection #:	7	Date Processed:	20.08.2018 15:32:58 GMT-4
Injection Volume:	5,00 ul	Channel Name:	W2489 ChA
Run Time:	20,00 Minutes	Channel Desc.:	W2489 ChA 254nm
Column Type:		Sample Set Name	

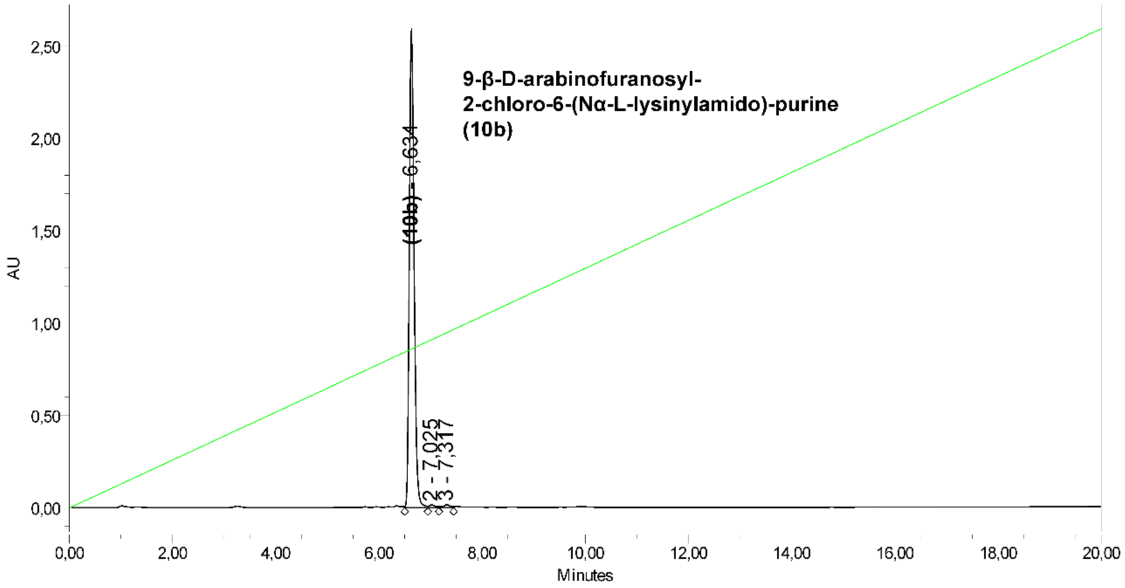

	Peak Name	RT (min)	Area (µV*sec)	% Area	Height (µV)	% Height
1	**10b**	6,634	16403915	98,42	2578532	98,86
2	2	7,025	119931	0,72	14461	0,55
3	3	7,317	142999	0,86	15234	0,58

Report Method: Gradient Overlay Report ASC

Page: 1 of 1

Printed: 20.08.2018 16:36:29 Etc/GMT-4

Figure SI-52. The chromatogram of **10b**

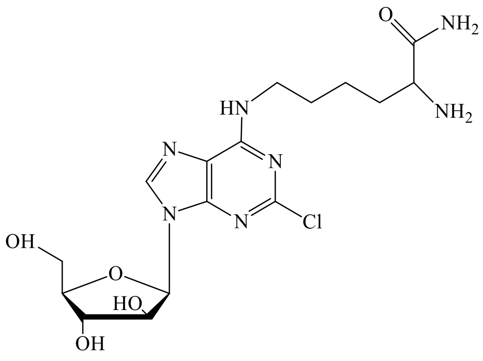

11b

9-*β*-*D*-arabinofuranosyl-2-chloro-6-(N$^{\varepsilon}$-L-lysinylamido)-purine (11b)

^{1}H NMR (700 MHz, DMSO-d_6, 30 °C): δ= 8.27 and 8.15 (br.t, 0.74H, J=5.1Hz, and sign., 0.26H, C6-NH), 8.19 (s, 1H, H-8), 7.24 (s, 1H, CO-NH), 6.84 (s, 1H, CO-NH), 6.16 (d, *J* = 4.6 Hz, 1H, H-1'), 5.52 (br.sign, 0.88H, 2'-OH), 5.62 (br.sign, 1H, 3'-OH), 5.05 (br.sign, 0.8H, 5'-OH), 4.17 (br.sign, 1H, H-2'), 4.11 (br.sign, 1H, H-3'), 3.84 and 3.41 (br.sign, 0.4H, and m, 1.6H, Cε-H), 3.78 (m, 1H, H-4'), 3.68 (m, 1H, H-5'a), 3.64 (m, 1H, H-5'b), 3.29 (sign., 2H, Cα-NH_2), 3.06 (br.t, 1H, Cα-H), 1.58 and 1.38 (m and m, 3H and 3H, Cβ-Ha and Cβ-Hb, Cγ-Ha and Cγ-Hb, Cδ-Ha and Cδ-Hb) ppm.

^{13}C NMR (176 MHz, DMSO-d_6, 30 °C) δ = 177.54 (CO-NH_2), 154.85 (C6), 152.84 (C2), 149.34 (C4), 140.19 (C8), 117.63 (C5), 83.91 (C1'), 83.62 (C4'), 75.49 (C2'), 74.60 (C3'), 60.60 (C5'), 54.49 (Cα), 39.67 (Cβ), 34.89 (Cε), 28.63 (Cδ), 22.71 (Cγ) ppm.

^{15}N NMR (71 MHz, DMSO-d_6, 30 °C): δ= 239.55 (N7), 226.37 (N1) 182.96 (Cα-NH_2) 168.83 (N9), 102.84 (CO-NH_2), 95.28 (C6-NH) ppm.

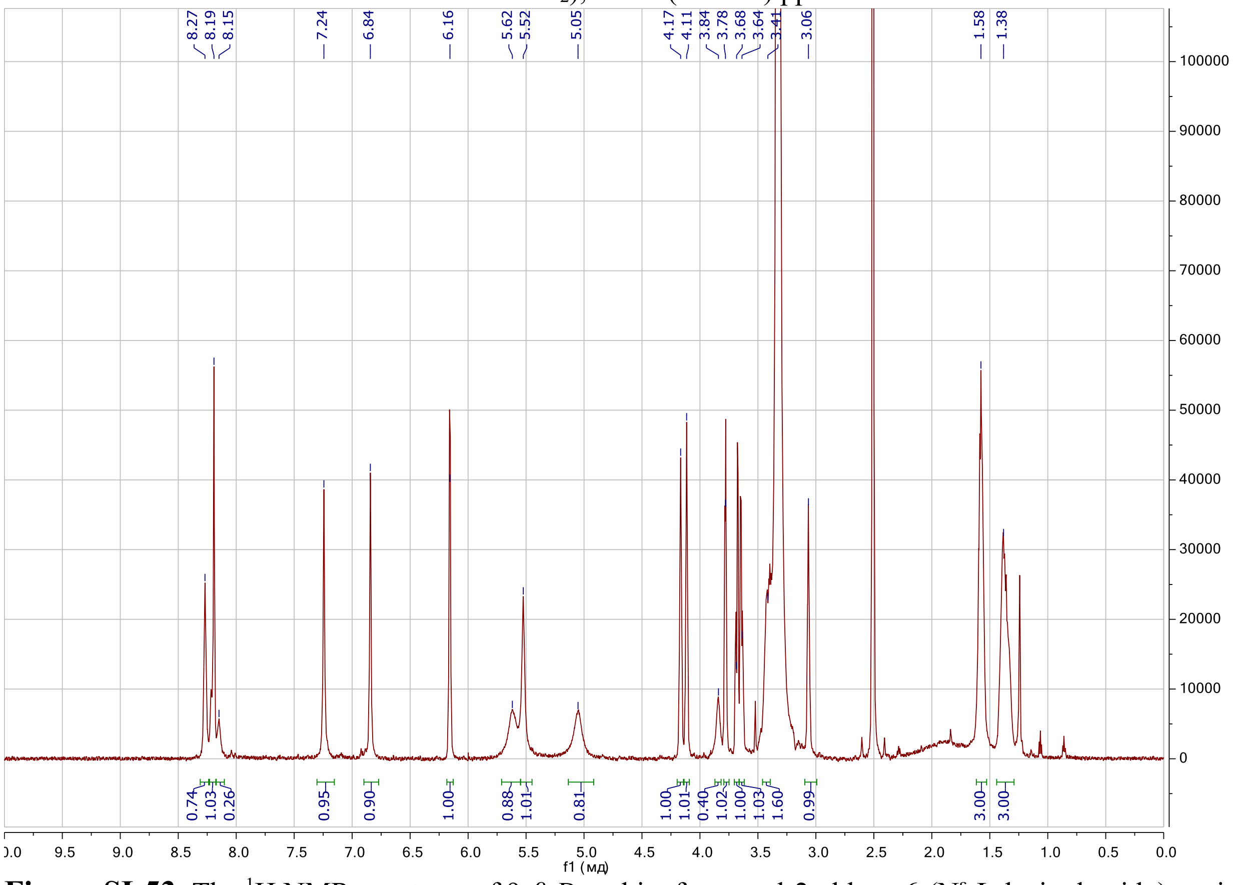

Figure SI-53. The ^{1}H NMR spectrum of 9-*β*-*D*-arabinofuranosyl-2-chloro-6-(N$^{\varepsilon}$-L-lysinylamido)-purine **(11b)**

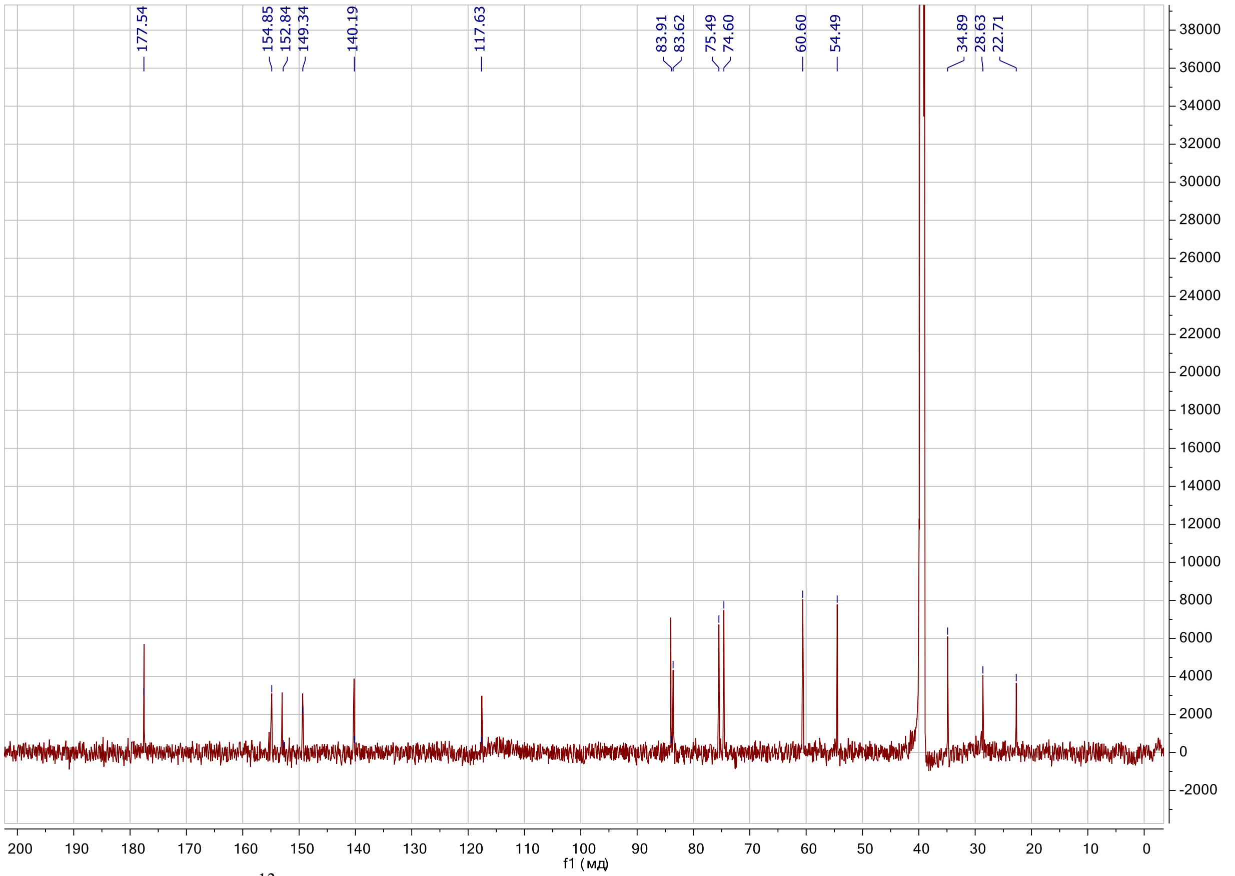

Figure SI-54. The ^{13}C NMR spectrum 9-*β*-*D*-arabinofuranosyl-2-chloro-6-(N^{ε}-L-lysinylamido)-purine **(11b)**

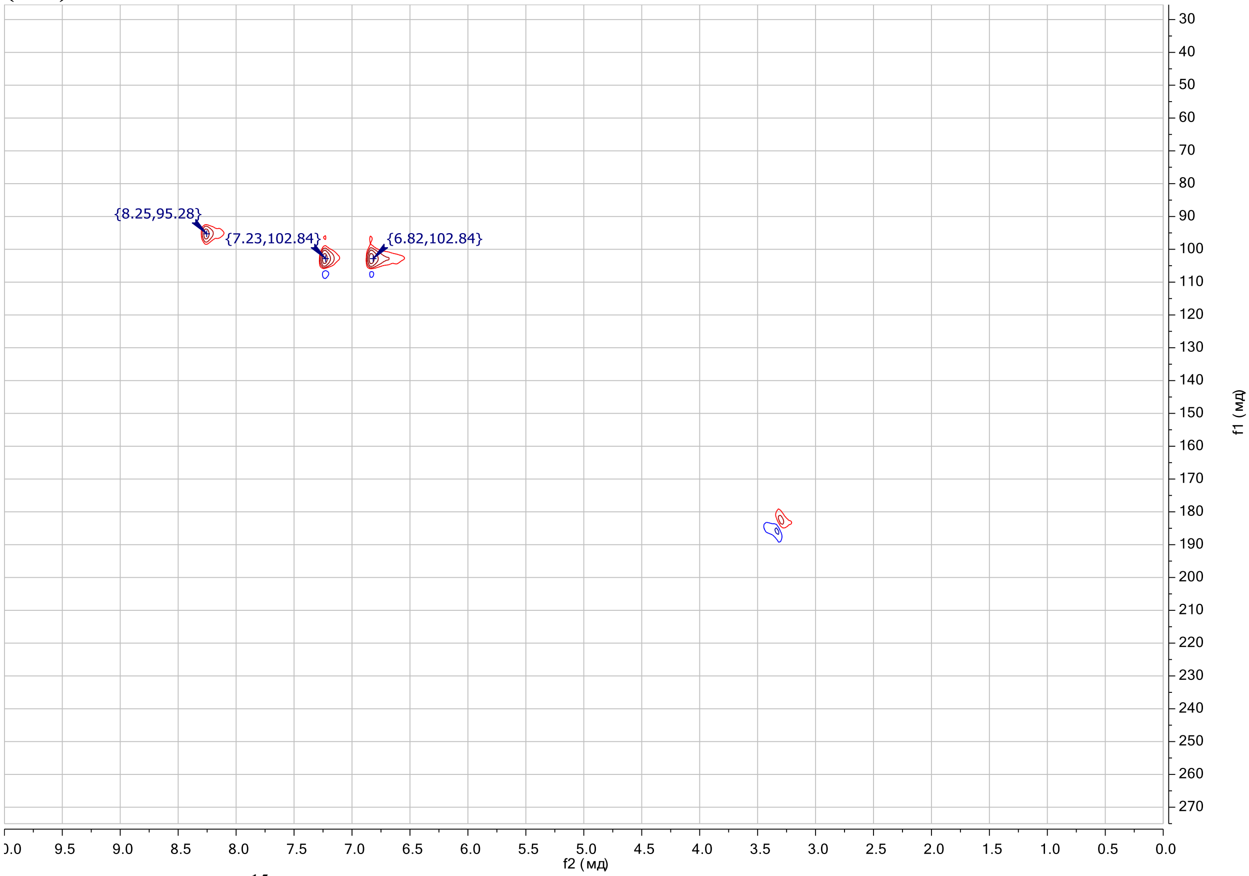

Figure SI-55. The ^{15}N HSQC NMR spectrum 9-*β*-*D*-arabinofuranosyl-2-chloro-6-(N^{ε}-L-lysinylamido)-purine **(11b)**

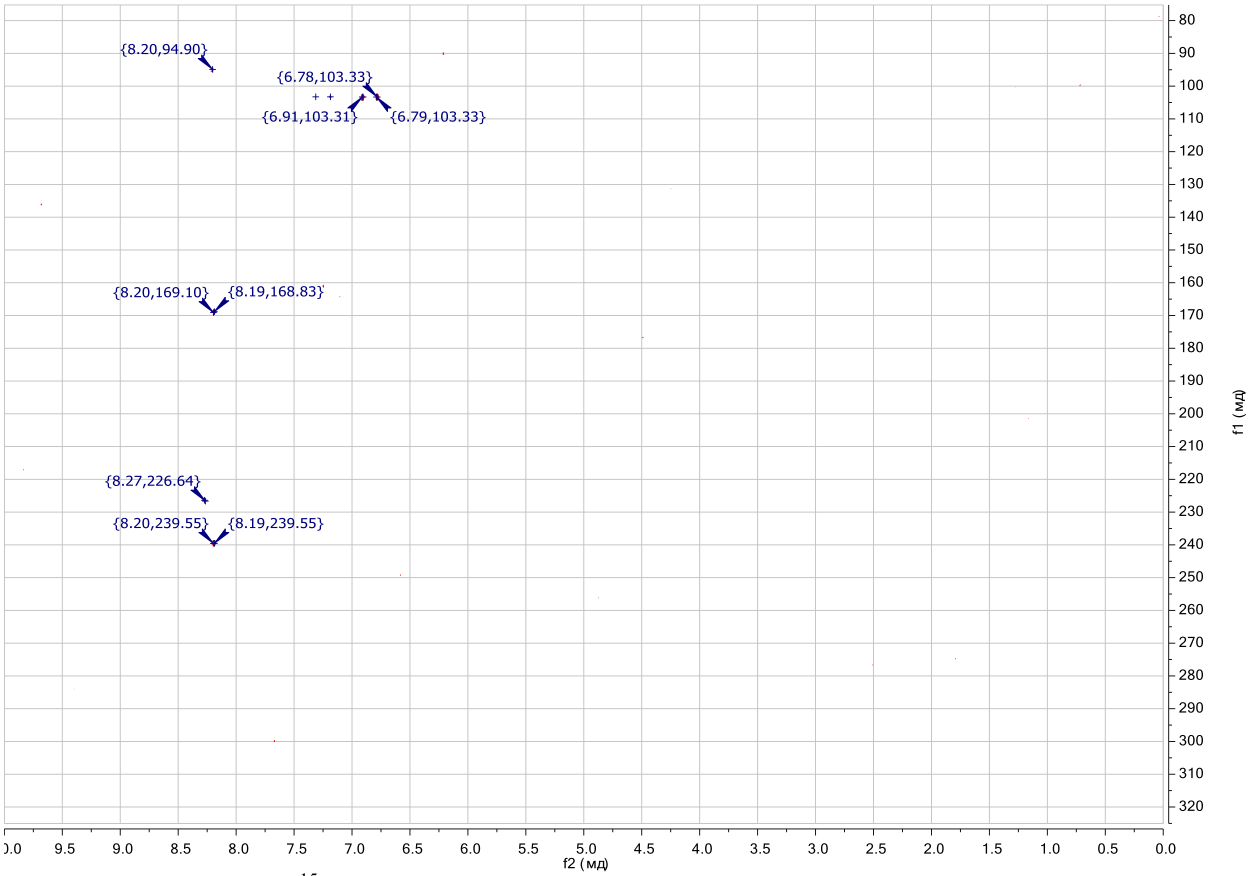

Figure SI-56. The ^{15}N HMBC NMR spectrum of 9-*β*-*D*-arabinofuranosyl-2-chloro-6-(N^{ε}-L-lysinylamido)-purine **(11b)**

IBCH RAS

Project Name Defaults

Reported by User: Breeze user (Breeze)

Breeze 2 HPLC System

SAMPLE INFORMATION

Sample Name:	**11b**	Acquired By:	Breeze
Sample Type:	Unknown	Date Acquired:	03.06.2016 14:55:46 GMT-4
Vial:	1	Acq. Method:	100B_dual_280nm_05ml
Injection #:	2	Date Processed:	03.06.2016 15:19:11 GMT-4
Injection Volume:	2,50 ul	Channel Name:	W2489 ChA
Run Time:	20,00 Minutes	Channel Desc.:	W2489 ChA 254nm
Column Type:		Sample Set Name	

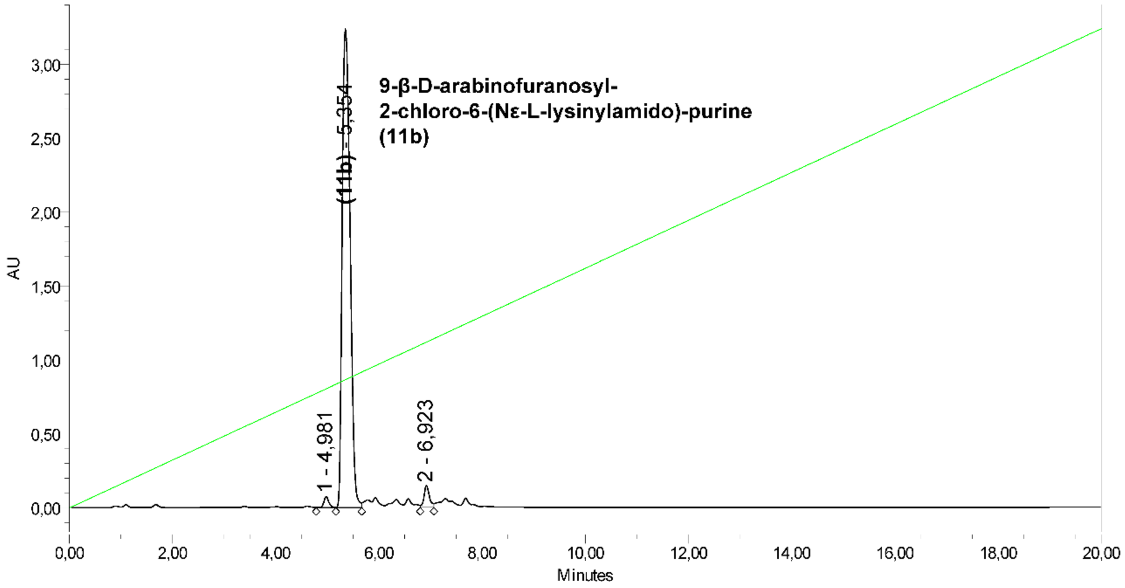

	Peak Name	RT (min)	Area (µV*sec)	% Area	Height (µV)	% Height
1	1	4,981	497034	1,37	73755	2,13
2	**11b**	5,354	34567547	95,62	3247593	93,59
3	2	6,923	1086716	3,01	148787	4,29

Report Method: Gradient Overlay Report ASC

Printed: 03.06.2016 15:19:28 Etc/GMT-4

Page: 1 of 1

Figure SI-57. The chromatogram of **11b**

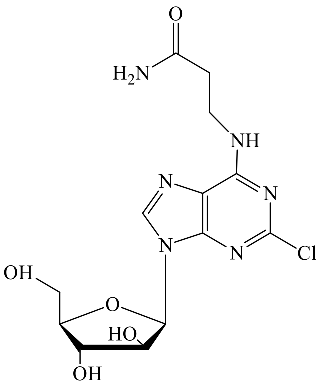

12b

9-β-D-arabinofuranosyl-2-chloro-6-(N-(3-amino-3-oxopropyl))-purine (12b)

^{1}H NMR (700 MHz, DMSO-d_6, 30 °C): δ= 8.24 and 8.21 (2 sign., 0.18H and 0.82, H-8), 8.14 and 8.04 (br.t, 0.8H, and m, 0.15H, C6-NH), 7.35 and 7.31(2 sign., 0.80H and 0.20H, H-8, CO-NH), 6.84 (s, 1H, CO-NH), 6.16 (br.d, 1H, H-1'), 5.62 (br.d., 1H, 2'-OH), 5.52 (br.d., 1H, 3'-OH), 5.05 (br.sign, 1H, 5'-OH), 4.17 (m, 1H, H-2'), 4.12 (m, 1H, H-3'), 4.04 (m, 0.39H, CαH-a) 3.78 (m, 1H, H-4'), 3.69 (m, 1H, H-5'a), 3.65 (m, 1H, H-5'b), 3.63 (m, 1.54H, CαH-b) 2.44. (t, J = 7.1 Hz, 2H, CβH) ppm.

^{13}C NMR (176 MHz, DMSO-d_6, 30 °C): δ= 172.43 (CO-NH_2), 154.71(C6), 152.93 (C2), 149.40 (C4), 140.44 (C8), 117.65 (C5), 84.01 (C4'), 83.62 (C1'), 75.48 (C2'), 74.55 (C3'), 60.58 (C5'), 36.57 (Cα), 34.27 (Cβ) ppm.

^{15}N NMR (71 MHz, DMSO-d_6, 30 °C): δ= 109.5 (CO-NH_2) ppm.

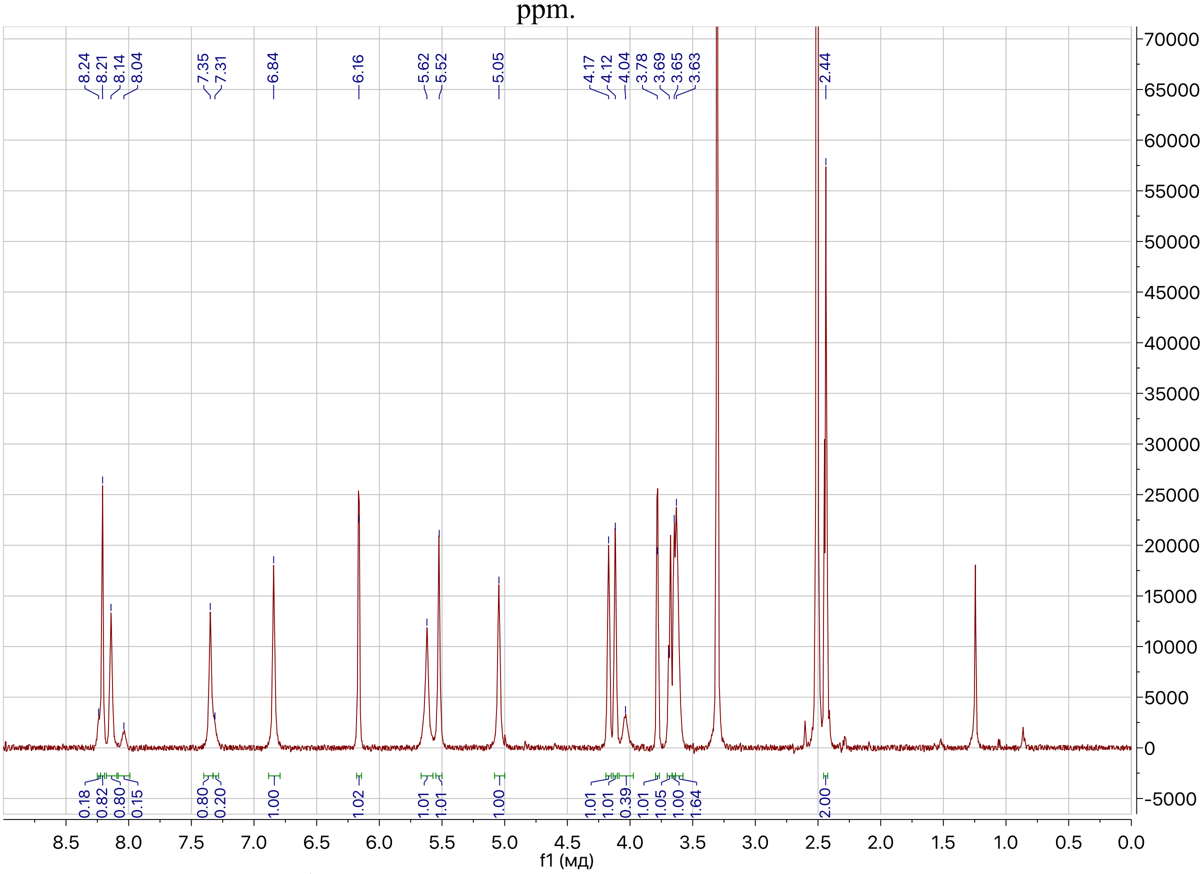

Figure SI-58. The ^{1}H NMR spectrum of 9-*β*-*D*-arabinofuranosyl-2-chloro-6-(*N*-(3-amino-3-oxopropyl))-purine **(12b)**

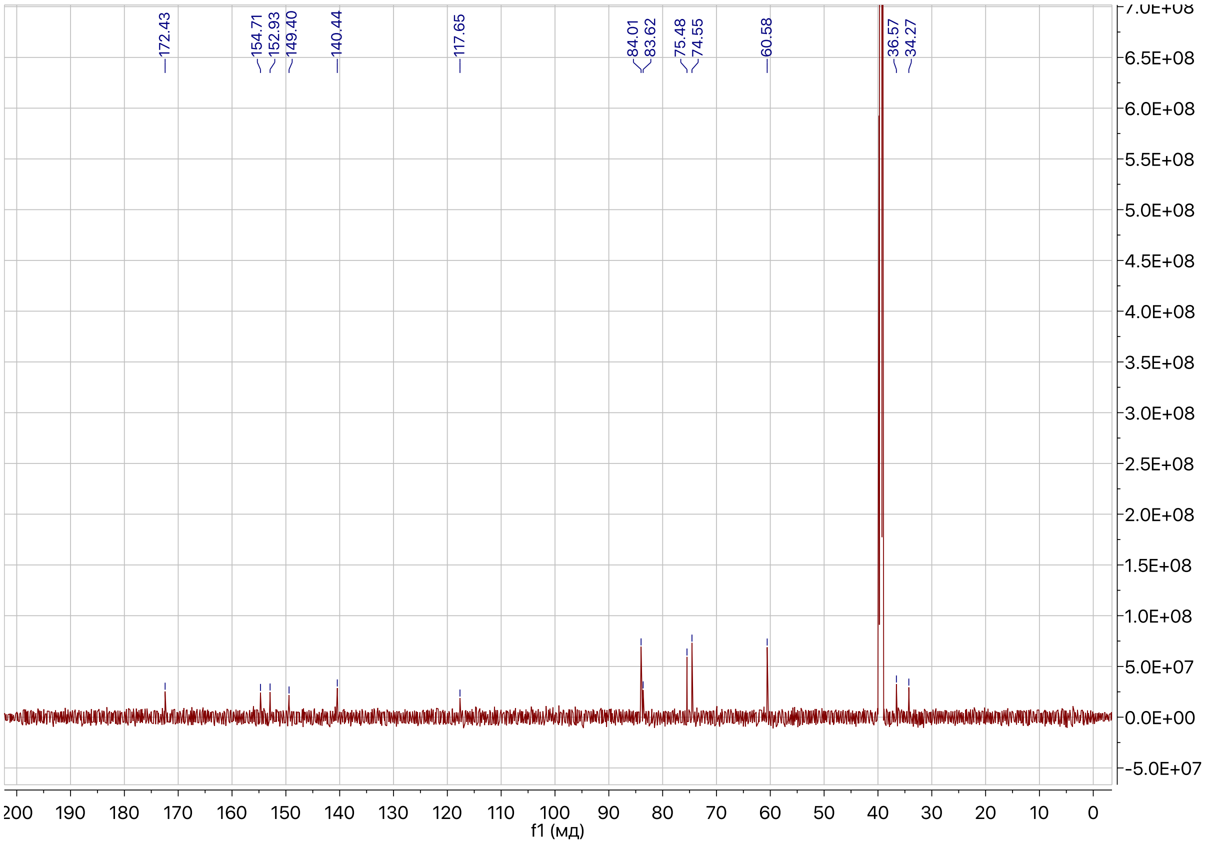

Figure SI-59. The ^{13}C NMR spectrum of 9-*β*-*D*-arabinofuranosyl-2-chloro-6-(*N*-(3-amino-3-oxopropyl))-purine **(12b)**

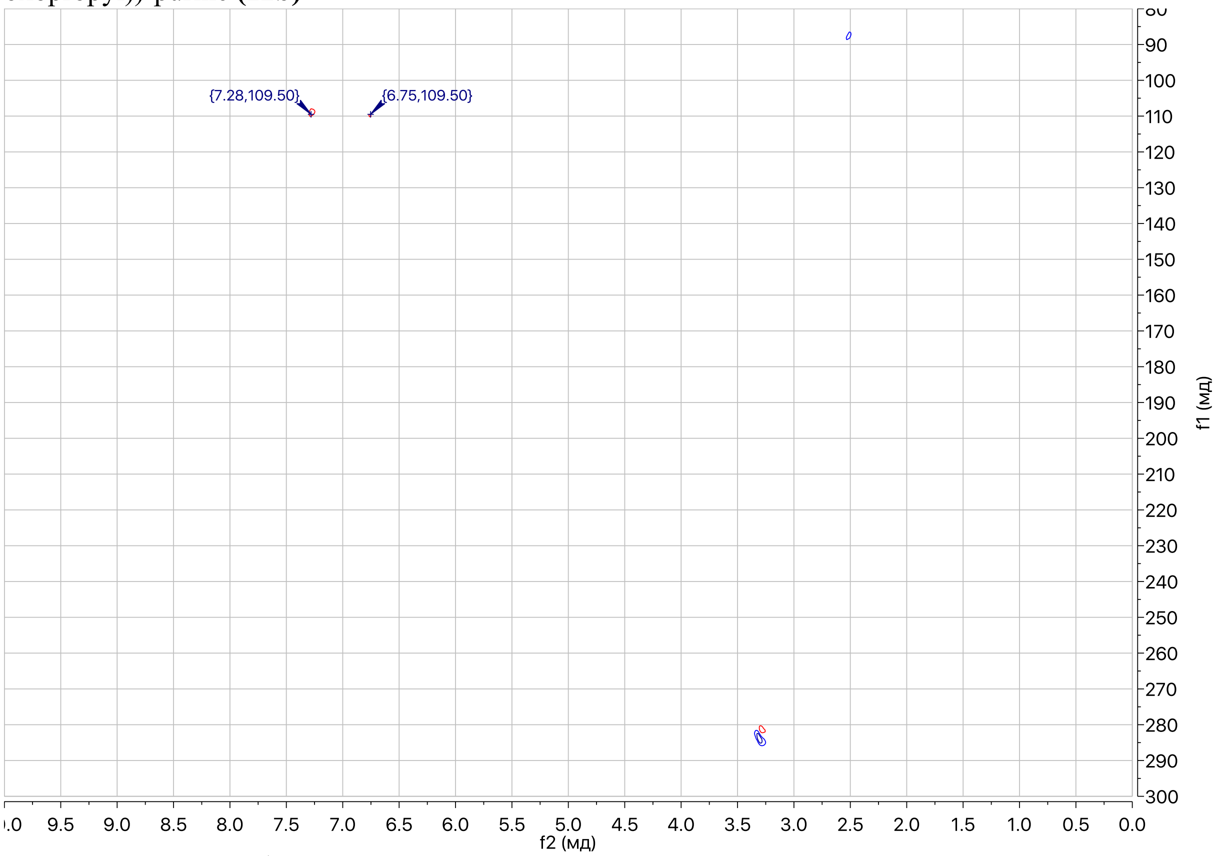

Figure SI-60. The ^{15}N HSQC NMR spectrum of 9-*β*-*D*-arabinofuranosyl-2-chloro-6-(*N*-(3-amino-3-oxopropyl))-purine **(12b)**

IBCH

Project Name: TESTLast

Reported by User: System

SAMPLE INFORMATION

Sample Name:	**12b**	Acquired By:	System
Sample Type:	Unknown	Date Acquired:	13.05.2013 18:59:08
Vial:	1	Acq. Method:	100B_dual_280
Injection #:	4	Date Processed:	13.05.2013 19:23:49
Injection Volume:	15,00 ul	Channel Name:	2487Channel 2
Run Time:	20,00 Minutes	Sample Set Name:	
Column Type:			

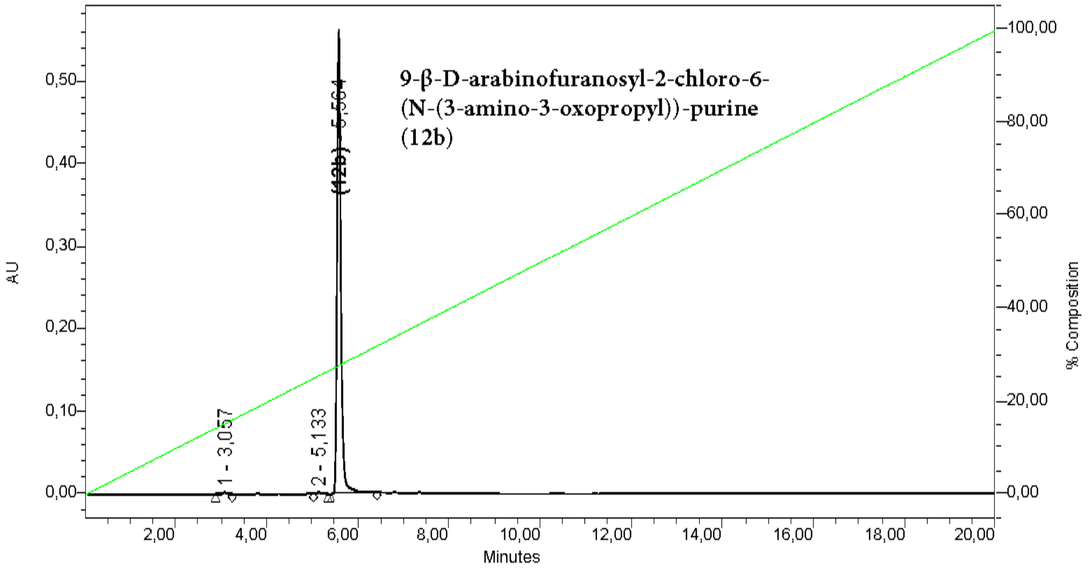

	Peak Name	RT (min)	Area (µV*sec)	% Area	Height (µV)	% Height
1	1	3,057	14808	0,45	2615	0,47
2	2	5,133	13713	0,42	1964	0,35
3	**12b**	5,564	3248284	99,13	556922	99,18

Report Method: Gradient Overlay Report ASC Printed 19:24:19 13.05.2013 Page: 1 of 1

Figure SI-61. The chromatogram of **12b**

Synthesis and NMR spectra of compounds 15 and 16

2-Chloro-9-(beta-D-ribofuranosyl)adenine (**15**)

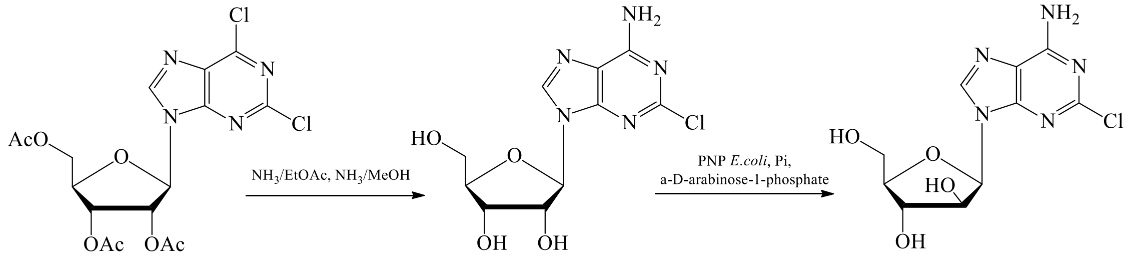

Scheme SI-1.

The method for synthesis of 2,6-dichloro-9-(2',3',5'-tri-*O*-acetyl-*β*-*D*-ribofuranosyl)purine was described earlier[1]. 2-chloradenosine was obtained from 2,6-dichloro-9-(2',3',5'-tri-*O*-acetyl-*β*-*D*-ribofuranosyl)purine according to a known method.

40 g (89.44 mmol) of 2,6-dichloro-9-(2',3',5'-tri-*O*-acetyl-*β*-*D*-ribofuranosyl)purine was dissolved in 1.2 L of anhydrous ethyl acetate and saturated with ammonia at 0 °C. The mixture was kept at 0°C for 3 days, then the residue was filtered off. Mixture of di-O-acetyl derivatives of 2-chloroadenosine was obtained (29.6 g, 86%) (LC-MS data, not shown). The residue was dissolved in 1.2 L of anhydrous methanol, and the solution was saturated with ammonia while stirring at room temperature, then cooled to 0 °C and kept for 5 days. The reaction mixture was evaporated, the residue was refluxed in 200 ml of a mixture of chloroform-methanol 3:1 and cooled. The precipitate was filtered off. 2-Chloradenosine (22.88 g, 85%) was obtained as white solid with a purity of 99.73% (HPLC data on a Nova-Pak column C18 4.6×150 mm, 5 µm, gradient 0-30% B over 20 minutes; m.p. 188-189 °C; λ_{max} 263.8 nm, extinction coefficient 13800. M/z: $[M+H]^+$: 302.0651 calcd, 302.0658 found. ^{1}H NMR (700 MHz, DMSO-*d*6, 30 °C): δ 8.39 (s, 1H, H-8), 7.83 (br.s, 2H, NH_2), 5.82 (d, J = 5.99 Hz, 1H, $H_{1'}$), 5.46 (d, J = 6.22 Hz, 1H, $OH_{2'}$), 5.18 (d, J = 4.96 Hz, 1H, $OH_{3'}$), 5.05 (t, J = 5.66 Hz, 1H, $OH_{5'}$), 4.53 (m, 1H, $H_{2'}$), 4.13 (m, 1H, $H_{3'}$), 3.95 (m, 1H, $H_{4'}$), 3.67 (m, 1H, $H_{5'a}$), 3.56 ppm (m, 1H, $H_{5'a}$).

1. Berzina, M.Y., et al., Synthesis of 2-chloropurine ribosides with chiral amino acid amides at C6 and their evaluation as A1 adenosine receptor agonists. Bioorganic Chemistry, 2022. **126**: p. 105878.

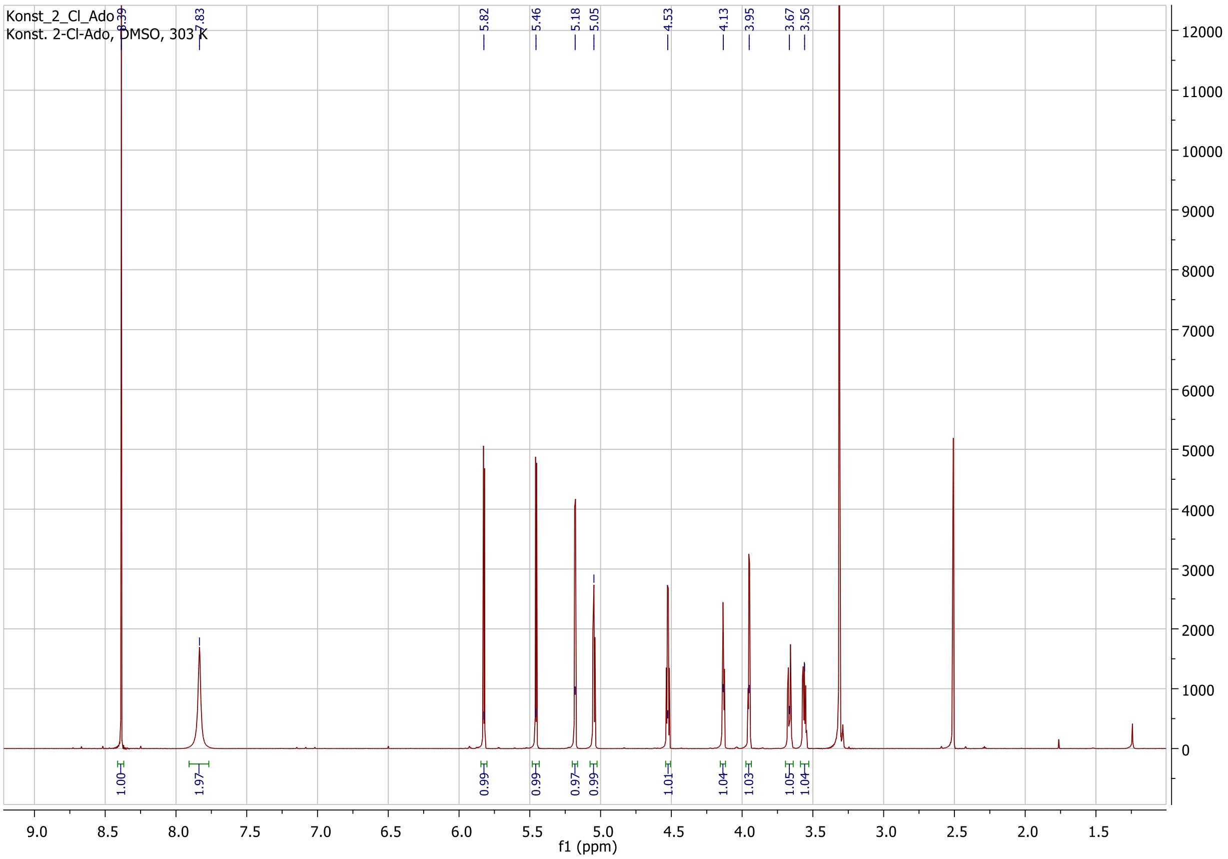

Figure SI-62. The ^{1}H NMR spectrum of 2-Cl-Ado

BT

Project Name: TESTLast_1

Reported by User: System

Breeze

SAMPLE INFORMATION

Sample Name:	2Cl-Ado-1	Acquired By:	System
Sample Type:	Unknown	Date Acquired:	16.11.2009 18:40:36
Vial:	1	Acq. Method:	30B_dual225_254
Injection #:	3	Date Processed:	16.11.2009 19:02:02
Injection Volume:	20,00 ul	Channel Name:	2487Channel 1
Run Time:	25,00 Minutes	Sample Set Name:	
Column Type:			

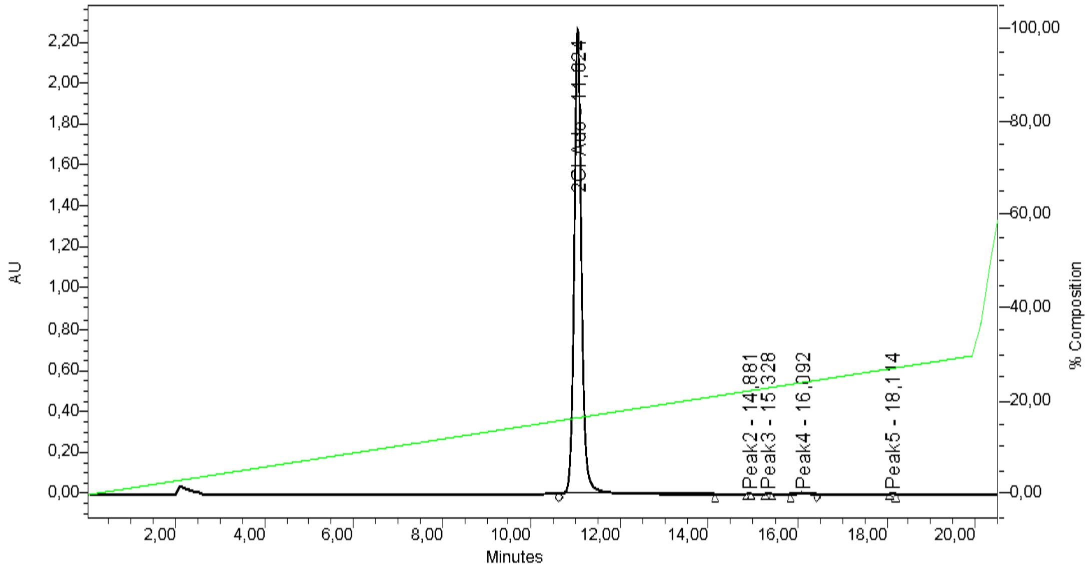

	Peak Name	RT (min)	Area (□V*sec)	% Area	Height (□V)	% Height
1	2Cl-Ado	11,024	26439137	99,73	2270864	99,70
2	Peak2	14,881	1428	0,01	375	0,02
3	Peak3	15,328	3676	0,01	650	0,03
4	Peak4	16,092	62072	0,23	5021	0,22
5	Peak5	18,114	4248	0,02	879	0,04

Report Method: Gradient Overlay Report ASC Printed 13:52:00 30.08.2022 Page: 1 of 1

Figure SI-63. The chromatogram of 2-Cl-Ado

The reaction conditions of 6-Amino-2-chloro-9-β-D-arabinofuranosyl-9H-purine (**16**) synthesis
0.2 g (0.65 mol) 2-Cl-Ado riboside, 0.15 g (1.1 mol) KH_2PO_4 and 1.922 g (1.6 mol) α-D-arabinose-1-phosphate were dissolved in 220 ml water 40-50°C and pH was adjusted up to 7.0. The enzyme PNP *E.coli* (166 e.u.) was added and the reaction mixtures were incubated at 50°C. Reactions were monitored by HPLC. When conversion reached the highest value 98.5%, the reaction was stopped by cooling to room temperature. The formed precipitate was filtered off on a glass filter, washed with cold water and dried. The target arabinoside was obtained in 65% yield (0.130 g, 0.42 mol) and 99.8% purity (Rt = 9.44 min, isocratic flow 7% eluent B 20 min, column Nova Pak C18, 4.6×150 mm, 5 mkm, flow rate 1 ml/min, Eluent A: water + 0.1% TFA. Eluent B: 70% acetonitrile: 30% water + 0.1% TFA). λ_{max} 262, 210 nm. ^{1}H NMR (700 MHz, DMSO-d_6, 30 °C): δ= 8.20 (s, 1H, H-8), 7.75 (s, 2H, NH_2), 6.16 (br.s., 1H, $H_{1'}$), 5.61 (br.s., 1H, $OH_{2'}$), 5.51 (br.s., 1H, $OH_{3'}$), 5.04 (br.s., 1H, $OH_{5'}$), 4.17 (br.s., 1H, $H_{2'}$), 4.12 (br.s., 1H, $H_{3'}$), 3.78 (br.s., 1H, $H_{4'}$), 3.68 and 3.65 ppm (2 br.s., 2H, $H_{5'}$).

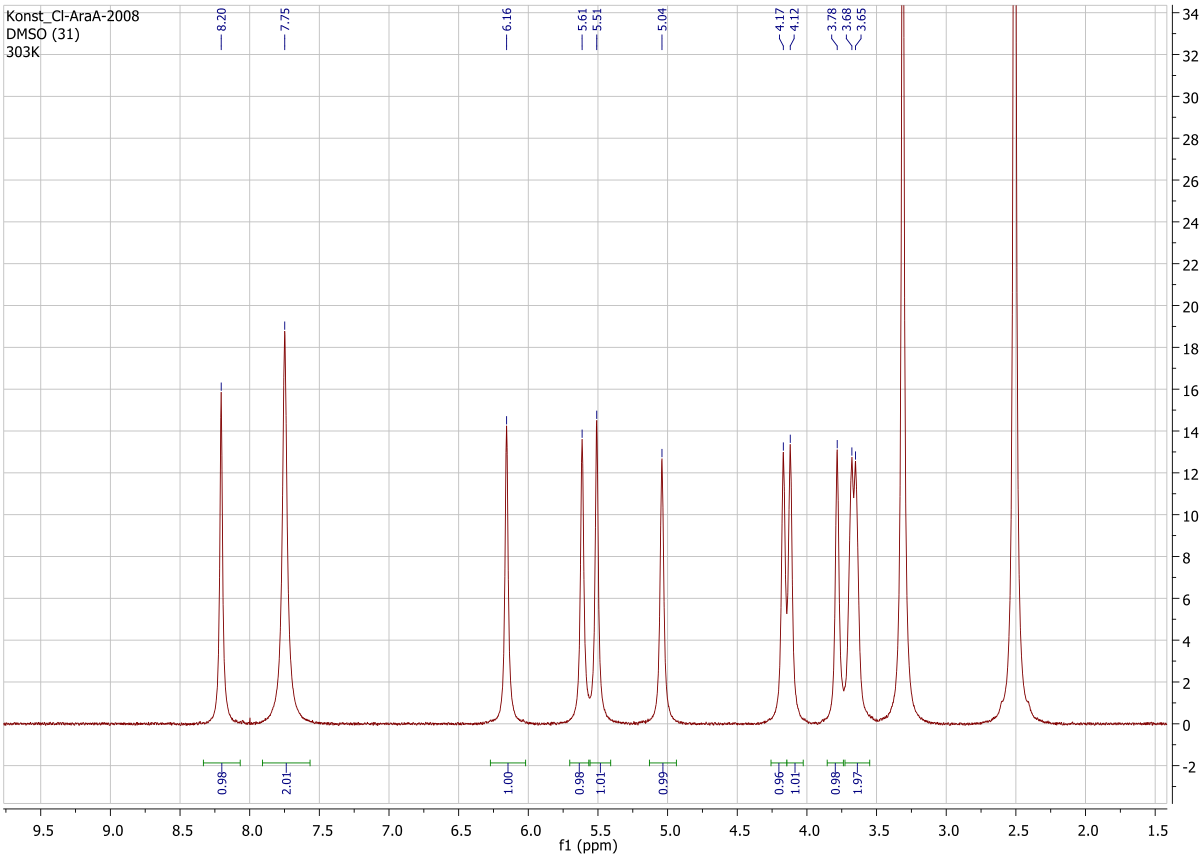

Figure SI-64. The ^{1}H NMR spectrum 6-Amino-2-chloro-9-β-D-arabinofuranosyl-9H-purine (2-Cl-AraA)

BT

Project Name: TESTLast_1
Reported by User: System

SAMPLE INFORMATION

Sample Name:	**2-Cl-AraA**	Acquired By:	System
Sample Type:	Unknown	Date Acquired:	13.10.2008 17:04:34
Vial:	1	Acq. Method:	iso ~7B%
Injection #:	1	Date Processed:	30.03.2009 14:33:40
Injection Volume:	15,00 ul	Channel Name:	2487Channel 1
Run Time:	18,00 Minutes	Sample Set Name:	
Column Type:			

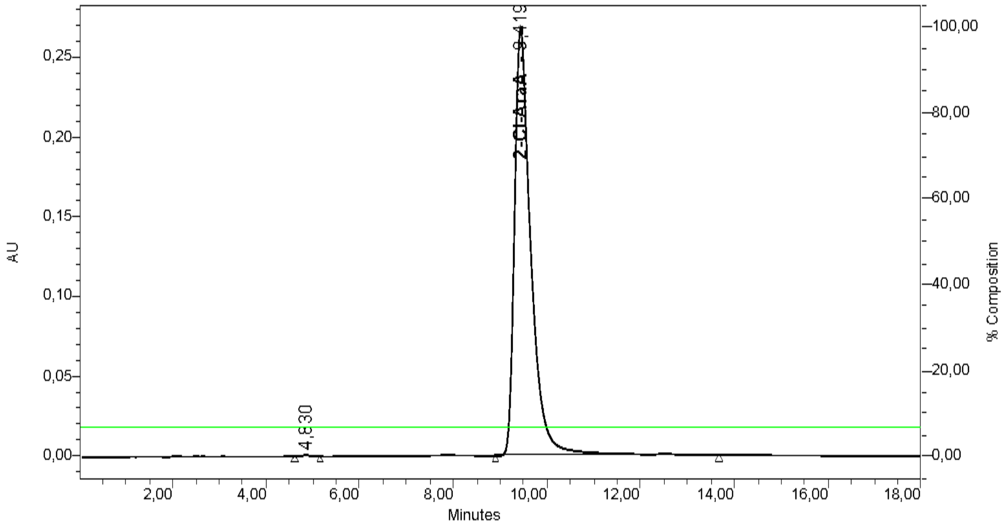

	Peak Name	RT (min)	Area (µV*sec)	% Area	Height (µV)	% Height
1		4,830	10816	0,16	895	0,33
2	**2-Cl-AraA**	9,419	6589178	99,84	268245	99,67

Report Method: Gradient Overlay Report ASC   Printed 17:10:22   08.06.2022   Page: 1 of 1

Figure SI-65. The chromatogram of 2-Cl-AraA

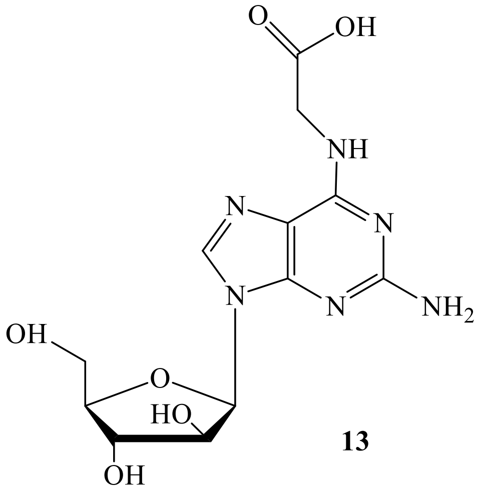 **13**	**2-amino-9-β-D-arabinofuranosyl-6-(N^{α}-glycinyl)-purine (13)** 100 mg N-(2-amino-9H-purin-6-yl)-glycine (Method for the synthesis heterobase was described in (Krasnov, Vigorov et al. 2015, Gruzdev, Vigorov et al. 2016), (0.4 mmol), 352 mg AraU (1.44 mol) was dissolved in 300 ml KH_2PO_4 buffer solution (326 mg, pH = 7.0). The enzyme 840 e.u. PNP *E.coli*, 816 e.u. UP *E.coli* was added and the reaction mixtures for 196 h. After removing the solvent from the reaction mixture in a vacuum (15 mm Hg), separation of the components was carried out on a Toyopearl DEAE-650C ion-exchange resin. The mobile phase: aqueous solution of NH_4HCO_3 (1.0 M, pH 7.5). Elution was carried out in two stages: with distilled water until the final release of trace amounts of Ura and AraU. Then, gradually increasing the concentration from 0.1 to 1.0 mmol, with a solution of ammonium bicarbonate. Fractions containing the reaction product were concentrated and dried in vacuo over phosphorus pentoxide. The target arabinoside was obtained in 58% yield (0.094 g, 0.276 mmol) and 99.01% purity (Rt = 4.9 min, gradient flow 0-70% B over 20 minutes, column Nova Pak C18, 4.6×150 mm, 5 mkm, flow rate 1 ml/min, Eluent A: water + 0.1% TFA. Eluent B: 70% acetonitrile: 30% water + 0.1% TFA). $[\alpha]_D^{25}$ = 12.40 º (c 0.5, H_2O). M.p. 248-250 °C; λ_{max} 281nm, extinction coefficient 7770. M/z $C_{12}H_{16}N_6O_6$: $[M+H]^+$: 341.1210 calcd, 341.1204 found.<br><br>^1H NMR (700 MHz, DMSO-d_6, 30 °C): δ= 12.43 (br.sign., 1H, COOH), 7.79 (s., 1H, H8), 7.31 (s., 1H, NH-CH2), 6.08 (br.sign., 1H, H1'), 5.87 (br.sign., 2H, NH2), 5.60 (br.sign., 1H, OH-2'), 5.45 (br.sign., 1H, OH-3'), 5.05 (br.sign., 1H, OH-5'), 4.08 (br.sign., 1H, H-3'), 4.07 (s., 2H, NH-CH2), 4.02 (s., 1H, H-2'), 3.74 (s., 1H, H-4'), 3.64 (br.sign., 1H, H-5'), 3.61 (br.sign., 1H, H-5') ppm.<br>^{13}C NMR (176 MHz, DMSO-d_6, 30 °C) δ= 151.5 (C4), 137.46 (C8), 113.2 (C5), 83.74 (C-1'), 75.88 (C-2'), 75.99 (C-3'), 41.61 (NH-CH2), 84.57 (C-4'), 61.48 (C-5') ppm.<br>^{15}N NMR (71 MHz, DMSO-d_6, 30 °C) δ= 239.02 (N-7), 162.89 (N-9), 78.52 (NH2) ppm.

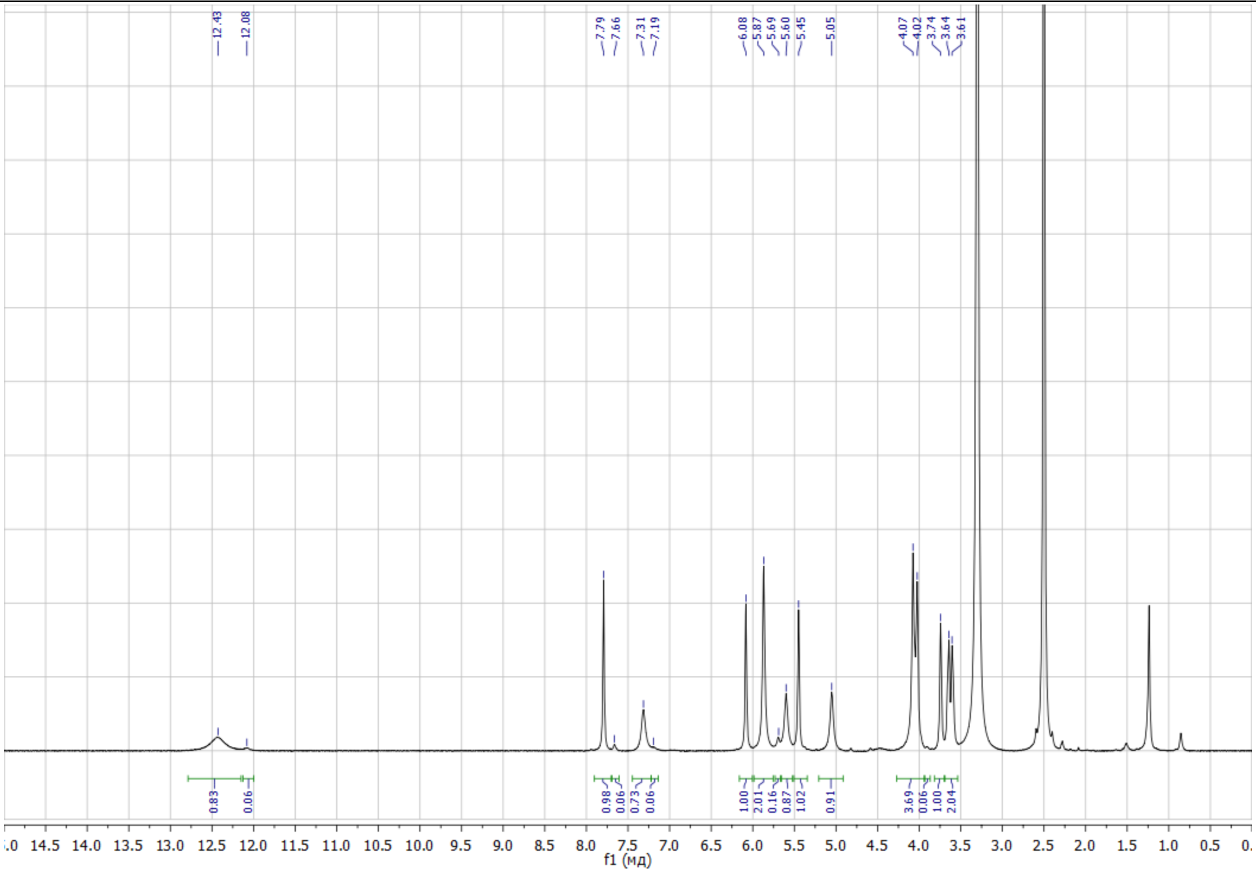

Figure SI-66. The ^{1}H NMR spectrum **13**

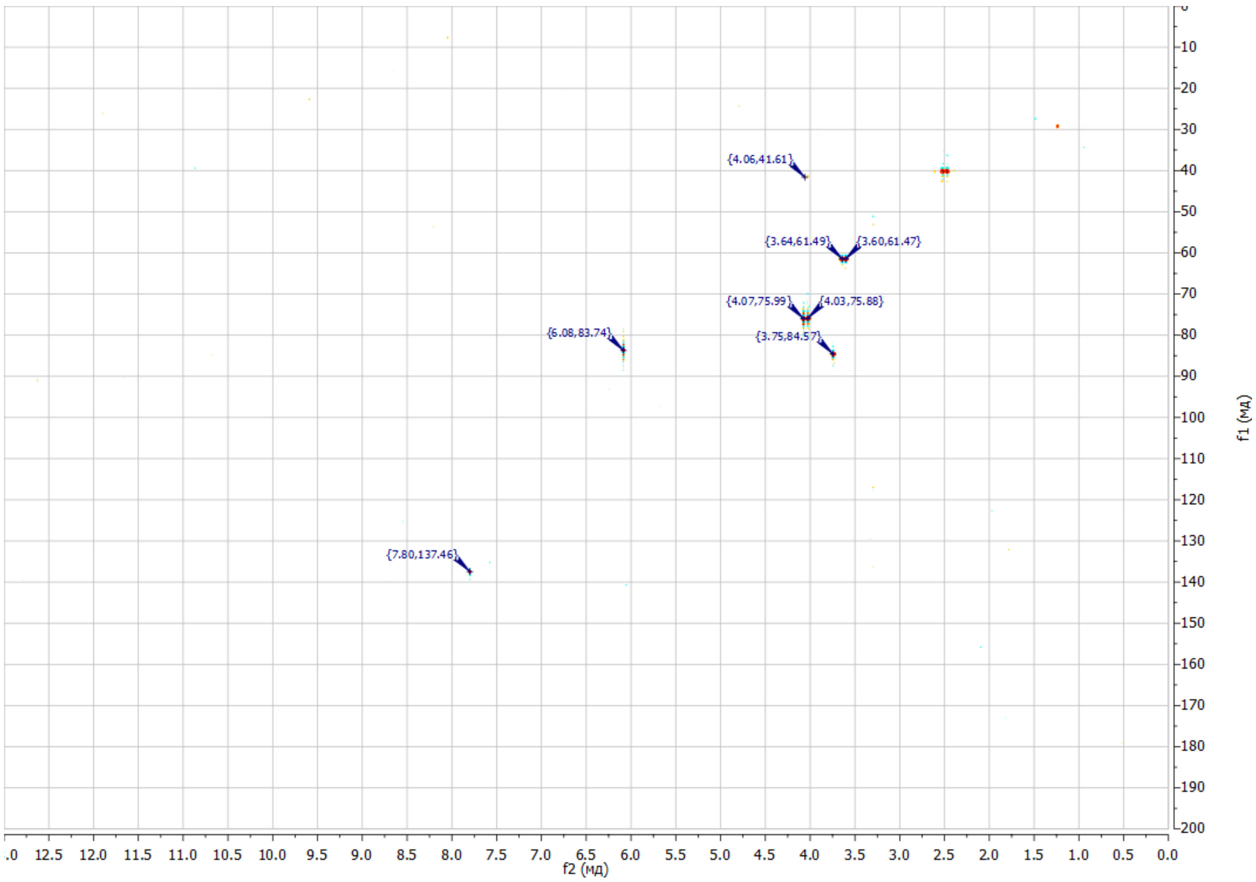

Figure SI-67. The ^{13}C HSQC NMR spectrum **13**

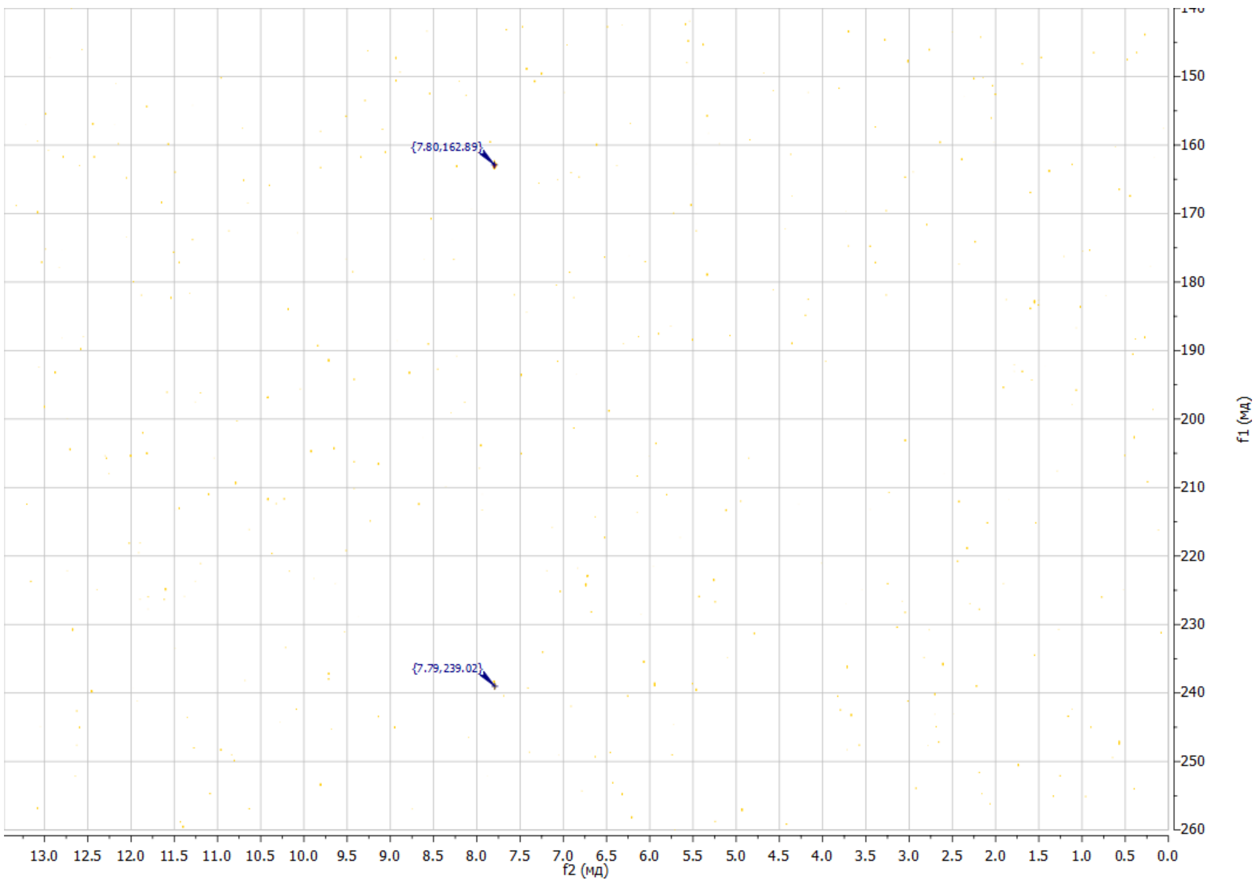

Figure SI-68. The ^{15}N HMBS NMR spectrum of **13**

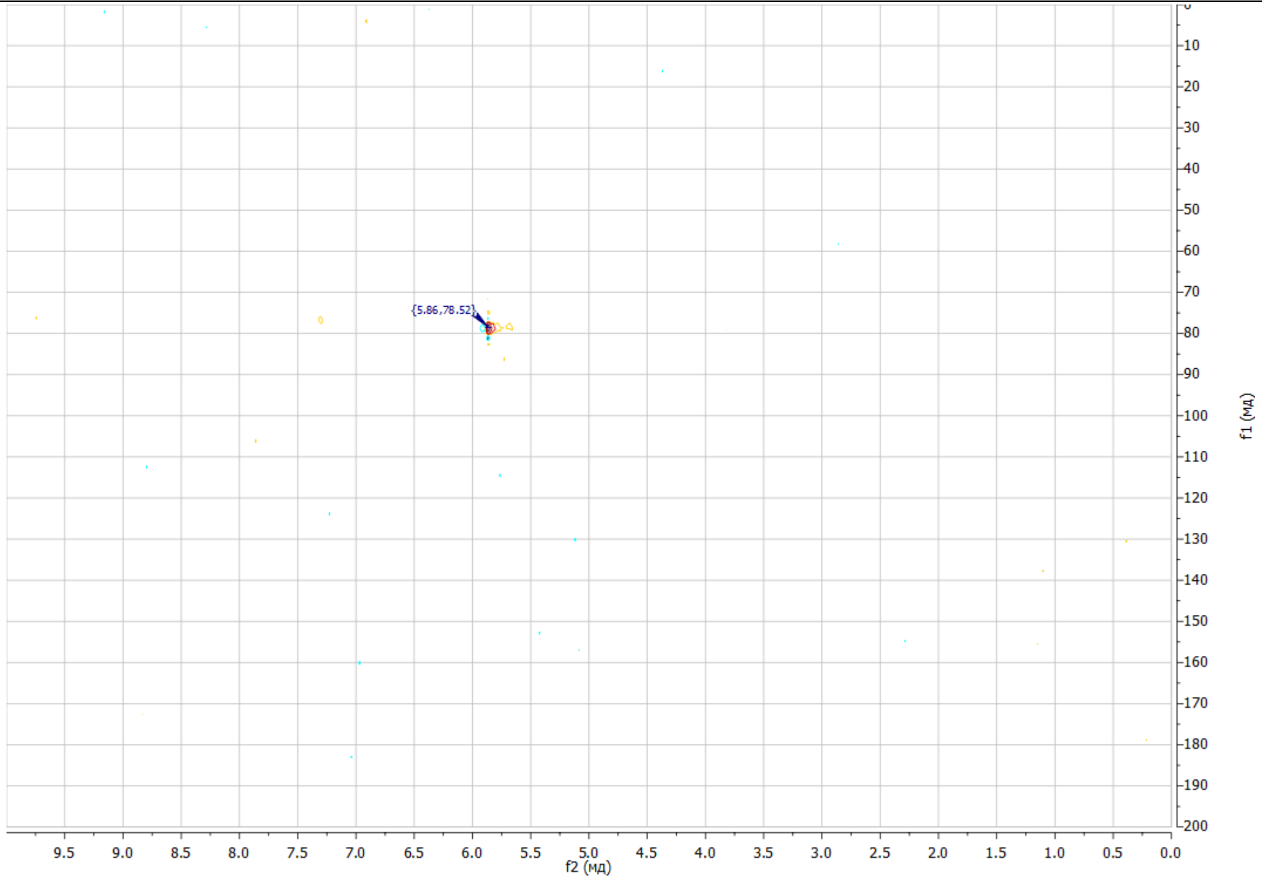

Figure SI-69. The ^{15}N HSQC NMR spectrum **13**

IBCH
Project Name: TESTLast
Reported by User: System

SAMPLE INFORMATION

Sample Name:		Acquired By:	System
Sample Type:	Unknown	Date Acquired:	24.06.2013 12:20:09
Vial:	1	Acq. Method:	70B_dual_280nm
Injection #:	1	Date Processed:	24.06.2013 12:30:38
Injection Volume:	10,00 ul	Channel Name:	2487Channel 1
Run Time:	15,00 Minutes	Sample Set Name:	
Column Type:			

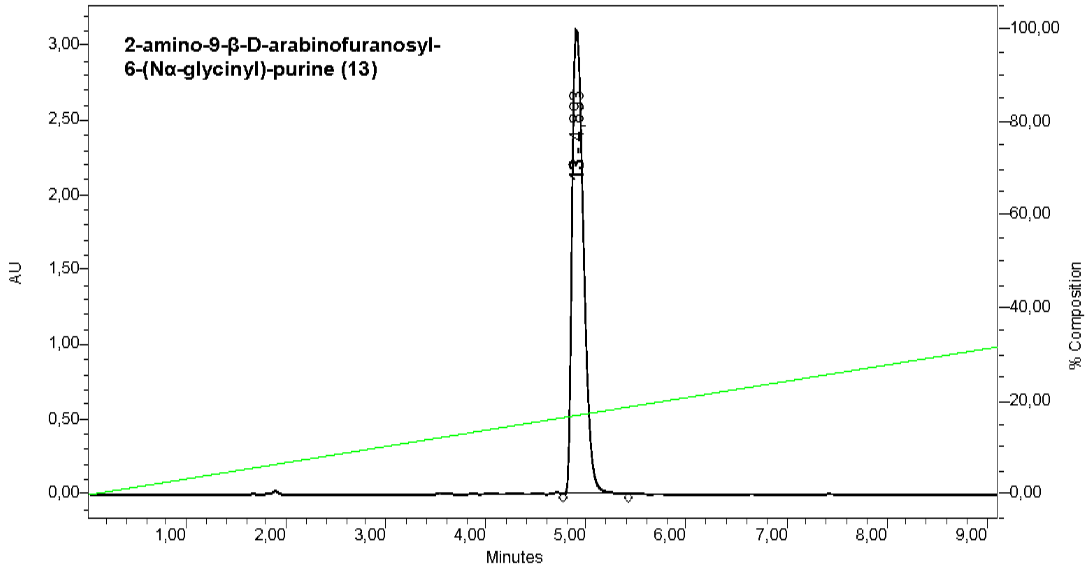

	Peak Name	RT (min)	Area (µV*sec)	% Area	Height (µV)	% Height
1	**13**	4,893	25103877	100,00	3172925	100,00

Report Method: Gradient Overlay Report ASC Printed 12:31:11 24.06.2013 Page: 1 of 1

Figure SI-70. The chromatogram

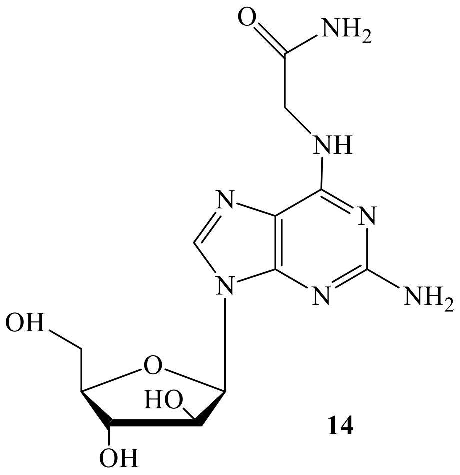 **14**	**2-amino-9-β-D-arabinofuranosyl-6-(N^{α}-glycinylamido)-purine (14)** A mixture of 35 mg (0.166 mmol) N-(2-amino-9H-purin-6-yl)-glycineamide (Method for the synthesis heterobase was described in (Krasnov, Vigorov et al. 2015, Gruzdev, Vigorov et al. 2016), 62 mg (0.25 mmol) AraU) was dissolved in 50 ml (10mM) KH_2PO_4 buffer solution (68 mg, pH = 7.0) was thermostated at 55°C for 196 h. The enzyme 140 e.u. PNP *E.coli*, 85 e.u. UP *E.coli* was added and the reaction mixtures for 196 h. After removing the solvent from the reaction mixture in a vacuum (15 mm Hg) separation of the components was carried out on a reverse phase sorbent Octadecyl=Si 100polyol (0.03 mm). The target compound was eluted with a gradient of 1-7% acetonitrile in water. Fractions containing the product were combined, the solvent was removed in vacuum (15 mm Hg). The product was dried in vacuum (5 mm Hg) over phosphorus pentoxide. The target arabinoside was obtained in 91% yield (0.052 g, 0.276 mmol) and 96.75% purity (Rt = 4.4 min, gradient flow 0-100% B over 20 minutes, column Nova Pak C18, 4.6×150 mm, 5 mkm, flow rate 1 ml/min, Eluent A: water + 0.1% TFA. Eluent B: 70% acetonitrile: 30% water + 0.1% TFA). $[\alpha]_D^{25}$ = 33.6 (c 0.125, H2O/DMSO 1:1). M.p. 168 -171°C; λ_{max} 281nm, extinction coefficient 8891. M/z $C_{12}H_{16}N_6O_6$: $[M+H]^+$: 340.1369 calcd, 340.1565 found.<br><br>^{1}H NMR (700 MHz, DMSO-d_6, 30 °C): δ= 7.79 (s, 1H, H-8), 8.12 and 8.01 (br.s., 0.65H, C6-NH), 7.27 (s, 1H, CO-NH), 7.01 (s, 1H, CO-NH), 6.08 (d, *J* = 4.6 Hz, 1H, H-1'), 5.87 (s., 2H, 2-NH_2), 5.61 (br.d, *J* ~3 Hz, 1H, 2'-OH), 5.46 (d, *J* = 3.8 Hz, 1H, 3'-OH), 5.06 (br.sign., 1H, 5'-OH), 4.08 (m, 1H, H-2'), 4.02 (m, 1H, H-3'), 3.98 (br.sign., 2H, NH-C<u>H</u>$_2$), 3.74 (m, 1H, H-4'), 3.65 (m, 1H, H-5'a), 3.60 (m, 1H, H-5'b) ppm.<br>^{13}C NMR (176 MHz, DMSO-d_6, 30 °C) δ= 160.48 (C4), 137.51 (C8), 113.26 (C5), 84.65 (C4'), 83.70

(C1’), 76.07 (C2’), 76.00 (C3’), 61.61 (C5’), 43.2 (NH-C̲H$_2$) ppm.

^{15}N NMR (71 MHz, DMSO-d_6, 30 °C) δ= 239.13 (N7), 197.54 (N1), 179.39 (N3), 163.11 (N9), 103.59 (CO-NH2), 78.96(NH2) ppm.

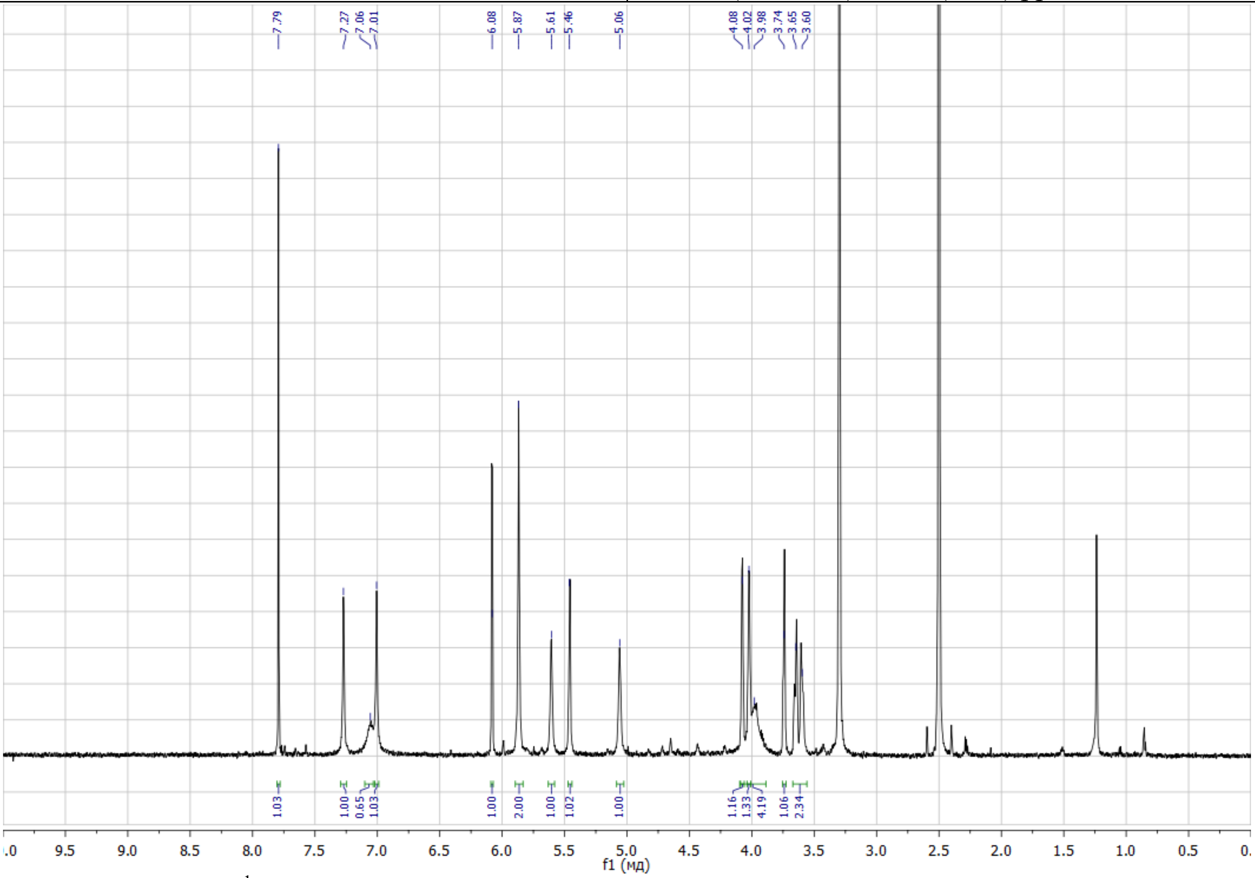

Figure SI-71. The ^{1}H NMR spectrum

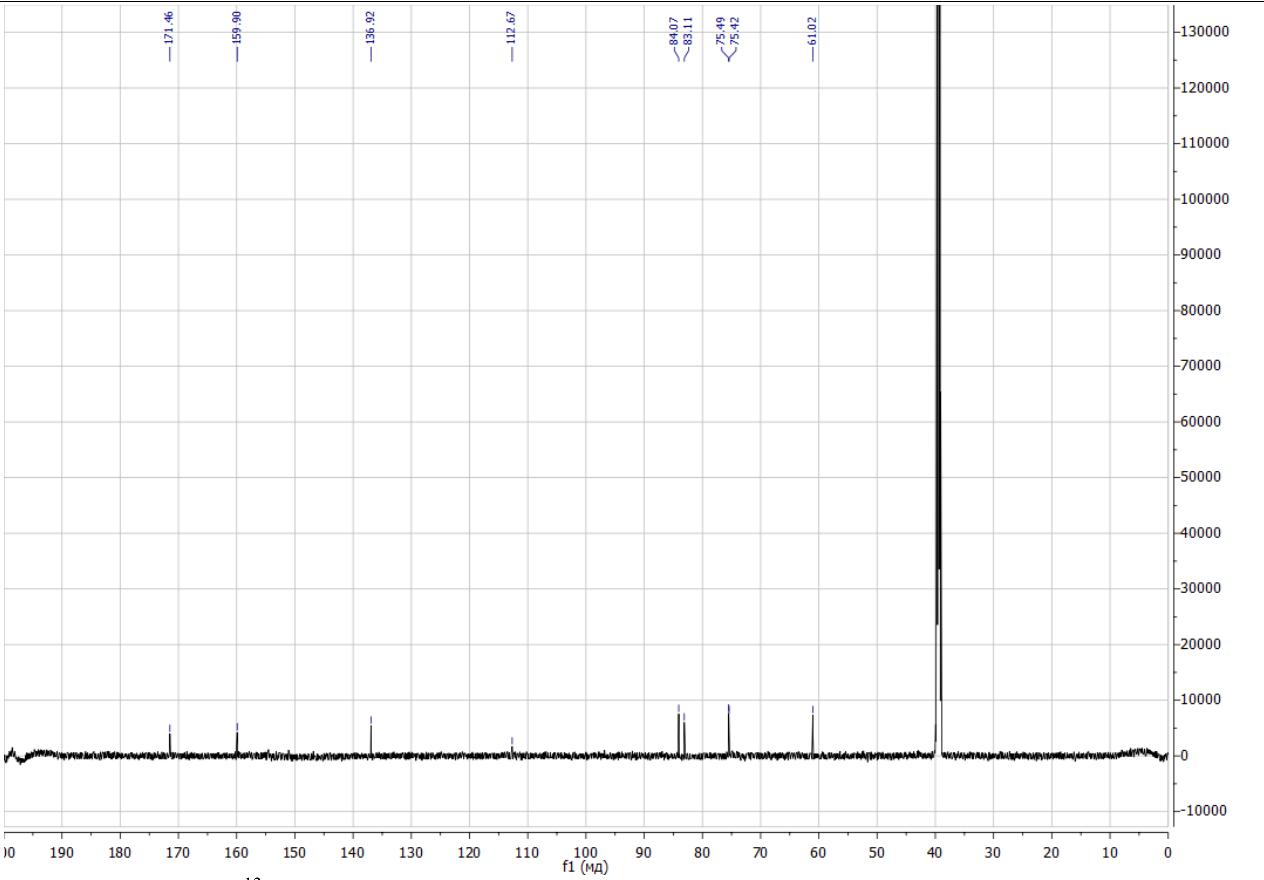

Figure SI-72. The ^{13}C NMR spectrum

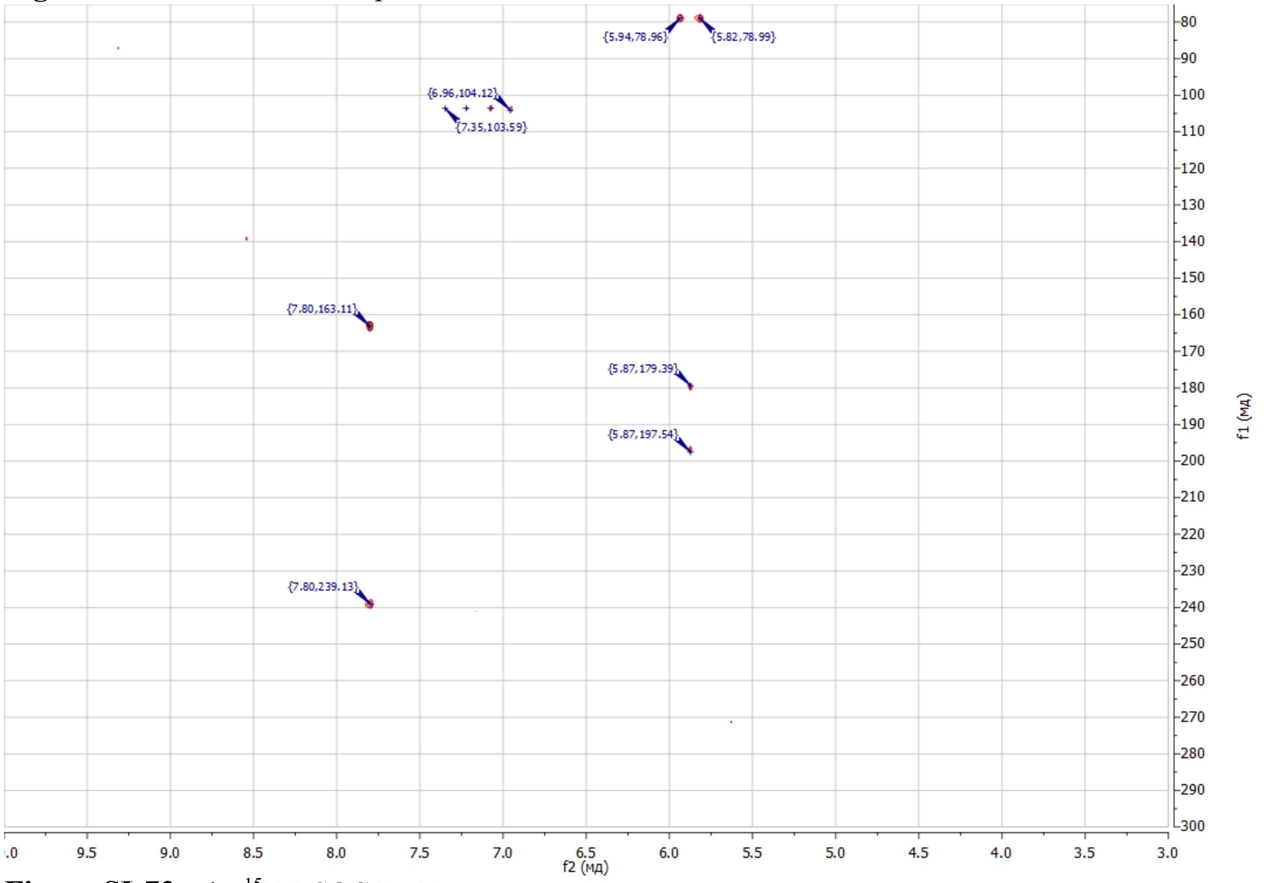

Figure SI-73. The ^{15}N HSQC NMR spectrum

BT

Project Name: TESTLast_1

Reported by User: System

S A M P L E   I N F O R M A T I O N

Sample Name:		Acquired By:	System
Sample Type:	Unknown	Date Acquired:	12.05.2014 11:33:59
Vial:	1	Acq. Method:	100B_dual_280
Injection #:	2	Date Processed:	19.05.2014 12:45:52
Injection Volume:	5,00 ul	Channel Name:	2487Channel 2
Run Time:	20,00 Minutes	Sample Set Name:	
Column Type:			

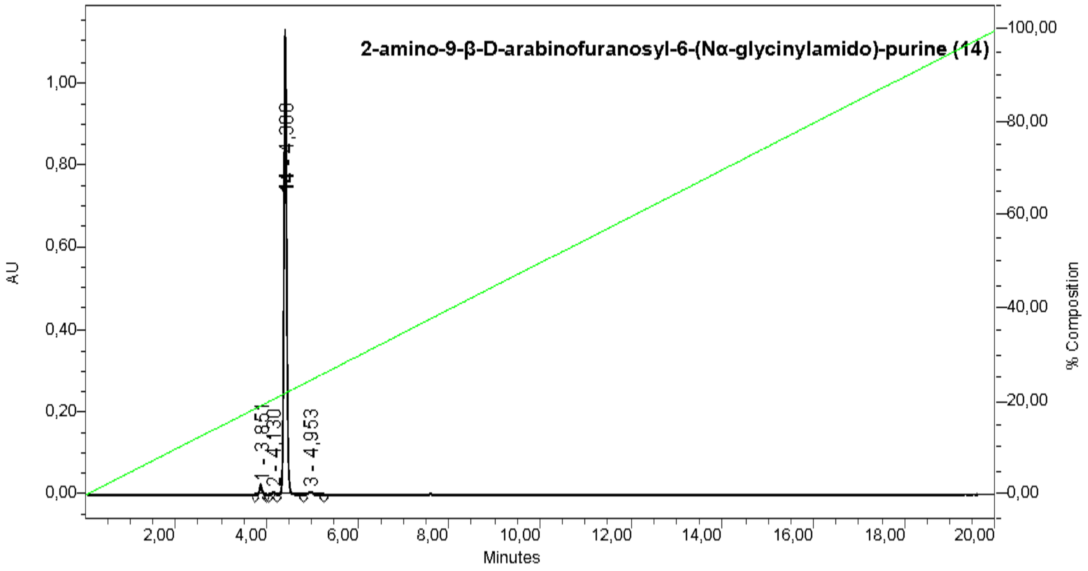

	Peak Name	RT (min)	Area (µV*sec)	% Area	Height (µV)	% Height
1	1	3,851	96489	1,99	22800	2,01
2	2	4,130	30208	0,62	6564	0,58
3	**14**	4,388	4659473	96,20	1097548	96,75
4	3	4,953	57231	1,18	7467	0,66

Report Method: Gradient Overlay Report ASC   Printed 12:45:59   19.05.2014   Page: 1 of 1

Figure SI-74. The chromatogram

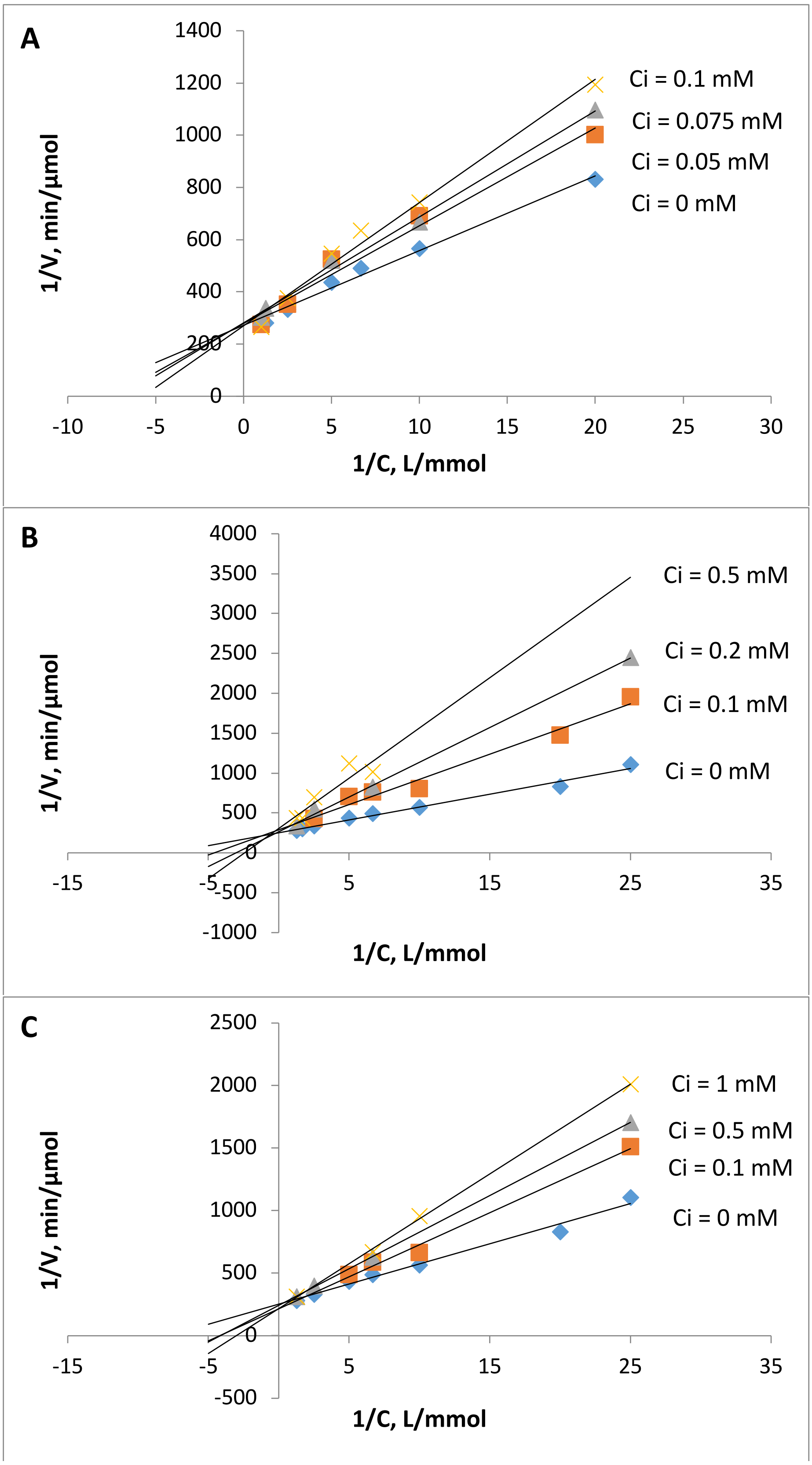

Figure SI-75. Lineweaver–Burk plots for the adenosine deamination at various concentrations of 2-chloroadenosine (**A**), 2-chloro-arabinoadenosine (**B**) and **4b** (**C**).
